# Frustrated Lewis Pair Mediated 1,2‐Hydrocarbation of Alkynes

**DOI:** 10.1002/anie.201705100

**Published:** 2017-07-04

**Authors:** Valerio Fasano, Liam D. Curless, James E. Radcliffe, Michael J. Ingleson

**Affiliations:** ^1^ School of Chemistry University of Manchester Oxford Road Manchester M13 9PL UK

**Keywords:** alkynes, carbocations, frustrated Lewis pairs, hydride transfer, 1,2-hydrocarbation

## Abstract

Frustrated Lewis pair (FLP) chemistry enables a rare example of alkyne 1,2‐hydrocarbation with N‐methylacridinium salts as the carbon Lewis acid. This 1,2‐hydrocarbation process does not proceed through a concerted mechanism as in alkyne syn‐hydroboration, or through an intramolecular 1,3‐hydride migration as operates in the only other reported alkyne 1,2‐hydrocarbation reaction. Instead, in this study, alkyne 1,2‐hydrocarbation proceeds by a novel mechanism involving alkyne dehydrocarbation with a carbon Lewis acid based FLP to form the new C−C bond. Subsequently, intermolecular hydride transfer occurs, with the Lewis acid component of the FLP acting as a hydride shuttle that enables alkyne 1,2‐hydrocarbation.

The functionalization of alkynes with borane Lewis acids is ubiquitous and best exemplified by the alkyne *syn*‐1,2‐hydroboration reaction (route A, Scheme 1).^1^ More recent studies have led to the development of other metal‐free alkyne hydroboration reactions (e.g. 1,1‐hydroboration, route B, and *trans*‐1,2‐hydroboration, route C).^2^, ^3^, ^4^, ^5^

**Scheme 1:**
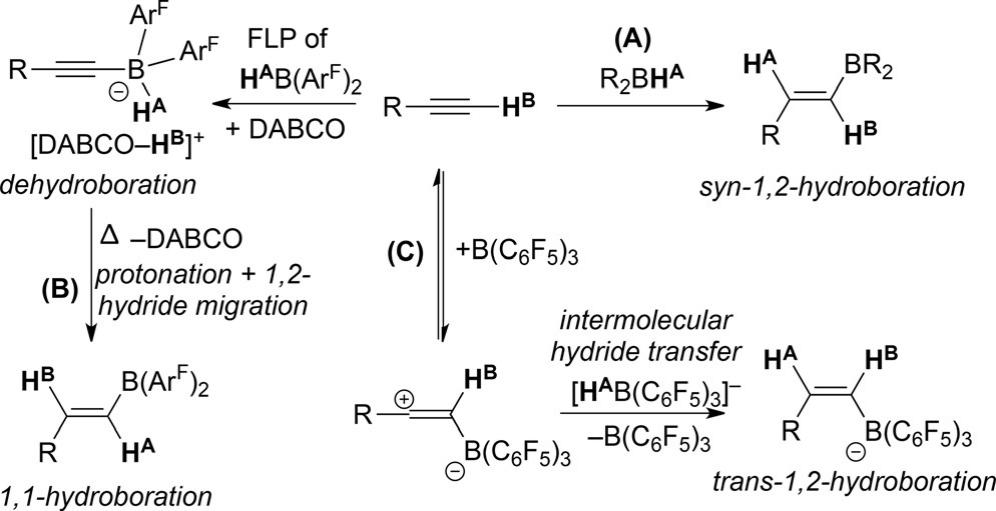
Metal‐free alkyne hydroboration reactions. Ar^F^=2,4,6‐(CF_3_)_3_C_6_H_2_; DABCO=1,4‐diazabicyclo[2.2.2]octane.

Carbenium ions are isoelectronic to boranes; however, the metal‐catalyst‐free hydrocarbation of alkynes remains extremely rare, in contrast to alkyne hydroboration, for reasons discussed in detail by Mayr and co‐workers.^6^ 1,2‐Hydrocarbation involves the addition of a C−H group of a [R_2_CH]^+^ electrophile across a triple bond and thus is distinct from the more common Lewis acid activation of an alkyne for a subsequent S_E_Ar reaction.^7^ To the best of our knowledge there is only one previous report of alkyne 1,2‐hydrocarbation, specifically, the addition of benzhydrylium cations to ynamides (Scheme 2).^6^ In this previous study, it was found that: a) alkyne 1,2‐hydrocarbation is a stepwise process, b) only highly nucleophilic alkynes were amenable, and c) hydrocarbation only occurs when the carbocation formed following 1,3‐hydride transfer is significantly stabilized. Therefore, alkyne hydrocarbation reactions are currently limited, and new mechanistic pathways for this conversion are highly desirable, as breakthroughs in this area will aid the development of new ways of creating C−C and C−H bonds in one step.

**Scheme 2:**
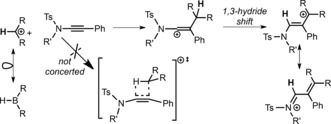
Stepwise 1,2‐hydrocarbation of ynamides by Mayr and co‐workers. Ts=*p*‐toluenesulfonyl.

One approach to access novel products from alkynes and Lewis acids is to treat alkynes with Lewis acid/Lewis base mixtures that form frustrated Lewis pairs (FLPs).^8^ This approach has led to alkyne dehydroelementation (e.g. dehydroboration, Scheme 1, top left) and 1,2‐*trans*‐addition of the FLP components to the alkyne (e.g. Scheme 3, top).^9^, ^10^, ^11^, ^12^ Whereas most FLPs contain boron‐based Lewis acids, cationic carbon Lewis acids can be effective in FLPs.^13^ Of specific relevance to this study is the reaction of alkynes with [Ph_3_C][BF_4_] in a FLP with (*o*‐tolyl)_3_P that resulted in either intramolecular Friedel–Crafts reactivity or 1,2‐addition to the alkyne (Scheme 3).^14^ In these cases, the absence of a C−H functionality at the electrophilic carbon center precludes hydrocarbation reactivity. As part of our studies on carbon Lewis acid based FLPs,^15^ we were interested in determining the reaction outcome(s) from combining the carbon Lewis acid *N*‐methylacridinium ([**1**]^+^) with alkynes, with and without additional Lewis bases. These experiments led to the discovery of a new FLP‐mediated route for the 1,2‐hydrocarbation of alkynes. The mechanism of this reaction is distinct from that reported by Mayr and co‐workers and those established for alkyne hydroboration reactions.

**Scheme 3:**
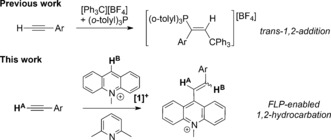
1,2‐Addition versus 1,2‐hydrocarbation with carbon Lewis acids.

For alkyne 1,2‐hydrocarbation by an analogous stepwise route to that reported by Mayr and co‐workers to be feasible, the cation formed after the 1,3‐hydride shift (the hydrocarbation product, [**2**]^+^) has to be more stable than the vinyl cation initially formed from the interaction of the carbon Lewis acid and the alkyne. Calculations at the M06‐2X/6‐311G(d,p) level (with PCM (CH_2_Cl_2_)) confirmed that this relationship was true for the combination of the *N*‐methylacridinium cation ([**1**]^+^) with 4‐ethynylanisole, with the 1,2‐hydrocarbation product, [**2**]^+^, being energetically favored over the vinyl cation (Scheme 4).

**Scheme 4:**
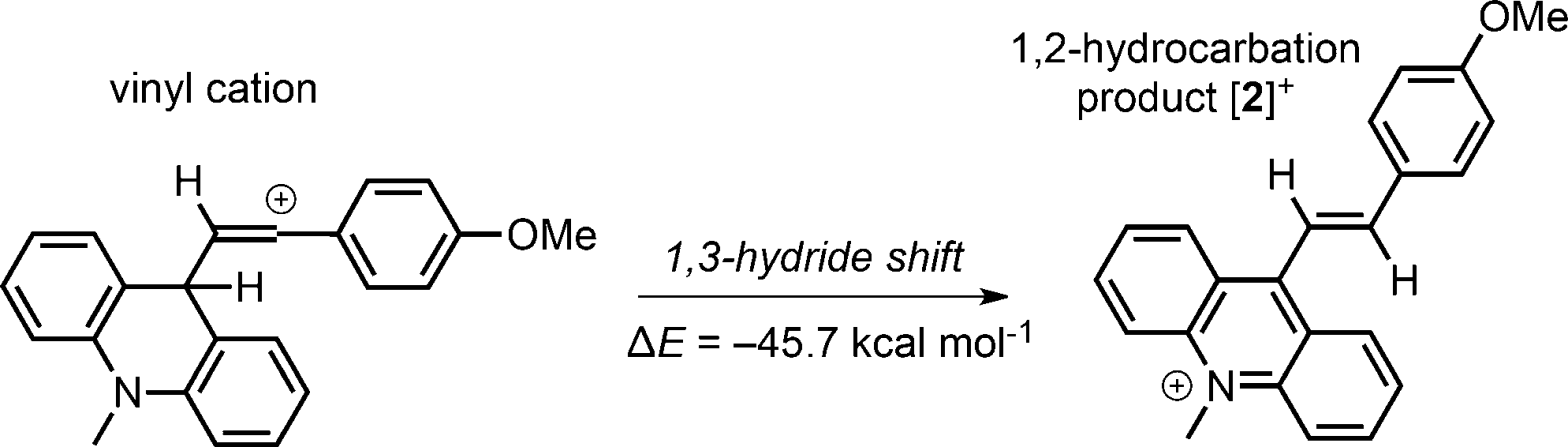
Energy difference between the vinyl cation (from the reaction of [**1**]^+^ with 4‐ethynylanisole) and the 1,2‐hydrocarbation product [**2**]^+^.

However, the combination of [**1**][BArCl] ([BArCl]=[B(3,5‐Cl_2_C_6_H_3_)_4_]^−^; this anion was chosen as it was an effective counterion in previous FLP chemistry with [**1**]^+^)^15^ and 4‐ethynylanisole led to no reaction in dichloromethane (DCM) at 20 °C or on heating at 60 °C (in a sealed tube). This result is in contrast to the reactivity of benzhydrylium salts and ynamides (which react at 20 °C) and is presumably due to the lower electrophilicity of [**1**]^+^ (relative to benzhydrylium salts) and the lower nucleophilicity of 4‐ethynylanisole (relative to ynamides), thus resulting in a larger kinetic barrier, which prevents hydride migration in this case despite the favorable thermodynamics.^16^ Furthermore, an attempt at *trans*‐hydrocarbation with mixtures of [**1**]^+^, *N*‐methylacridane (as a hydride source), and 4‐ethynylanisole (analogous to pathway C, Scheme 1) led to complex mixtures with minimal hydrocarbation product observed. In contrast, an equimolar mixture of [**1**][BArCl], 2,6‐lutidine (which forms an FLP),15a and 4‐ethynylanisole resulted in a slow reaction. After 48 h at 20 °C in DCM, only partial consumption of [**1**][BArCl] had occurred; nevertheless, crystallization (by layering the sample with pentane) and X‐ray diffraction studies revealed formation of the *Z* 1,2‐hydrocarbation product [**3**][BArCl] (Scheme 5, left). The reaction was accelerated at higher temperature (60 °C, 72 h) but led to the observation of a different product in the ^1^H NMR spectrum, consistent with the *trans* isomer, [**2**]^+^ (which has two diagnostic vinylic doublets with ^3^ *J*_H,H_=16 Hz). The formation of the *E* 1,2‐hydrocarbation product [**2**][BArCl] was confirmed by NMR spectroscopy, mass spectrometry, and X‐ray diffraction studies. Identical outcomes were observed when the reactions were performed in the dark, and during these reactions *N*‐methylacridane (**1‐H**) and a new acridinium species were observed as intermediates.

**Scheme 5:**
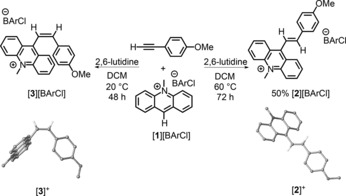
Synthesis of the alkyne 1,2‐hydrocarbation products [**2**][BArCl] and [**3**][BArCl], and solid‐state structures of [**2**]^+^ and [**3**]^+^.

As no hydrocarbation reaction proceeded in the absence of 2,6‐lutidine, a concerted mechanism (analogous to *syn*‐1,2‐hydroboration of alkynes with R_2_BH) and a stepwise mechanism (analogous to the 1,2‐hydrocarbation of ynamides with benzhydrylium salts) are precluded. The FLP of [**1**]^+^/2,6‐lutidine could conceivably react with alkynes by a number of pathways, including dehydrocarbation or 1,2‐addition. To rule out the 1,2‐addition pathway, a non‐nucleophilic Lewis base was used instead of 2,6‐lutidine. On replacing 2,6‐lutidine with the hindered base 2,4,6‐tri‐*tert*‐butylpyridine (TBP), [**2**][BArCl] was again formed in situ as the major product from [**1**][BArCl]/4‐ethynylanisole (after 72 h at 60 °C). The formation of [**2**][BArCl] with TBP indicates that a deprotonation (dehydrocarbation) pathway is most likely, as alkyne 1,2‐addition products are precluded with this extremely hindered base. The most probable proton source is the Lewis acid activated alkyne (the vinyl cation, Scheme 4, left), which on deprotonation would yield the dehydrocarbation product **4** (Scheme 6) containing a new C−C bond, along with an equivalent of protonated base. Direct deprotonation of the alkyne by these two pyridine derivatives is not feasible owing to the large difference in the p*K*_a_ values of the pyridinium ions and the terminal alkyne (ca. 6 and ca. 25 in water, respectively).

**Scheme 6:**
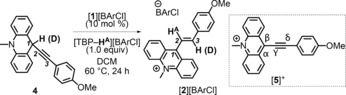
Synthesis of the 1,2‐hydrocarbation product [**2**][BArCl] from **4**.

To determine whether **4** was indeed a possible intermediate in the hydrocarbation pathway, an equimolar mixture of independently synthesized **4** and [TBP−H][BArCl] was dissolved in DCM, both in the presence and in the absence of [**1**][BArCl] (10 mol %). The latter variable was explored as the FLP‐mediated production of [**2**]^+^/[**3**]^+^ is slow; therefore, any intermediates formed during alkyne hydrocarbation (such as **4**) will exist in solution in the presence of [**1**]^+^. Upon mixing **4,** [TBP−H][BArCl], and [**1**][BArCl] (10 mol %), an immediate color change was noted, with [**1**][BArCl] completely converted into **1‐H** along with approximately 10 mol % formation of a new *N*‐methylacridinium species, proposed to be [**5**]^+^ (Scheme 6, inset), which was confirmed by subsequent independent synthesis. Upon heating this reaction mixture at 60 °C, [**2**][BArCl] was detected as the major product. When the reaction was repeated in the absence of [**1**][BArCl], conversion into [**2**][BArCl] still proceeded, although it was notably slower. These results are consistent with the 1,2‐hydrocarbation reaction proceeding through initial dehydrocarbation of the terminal alkyne by the FLP combination of [**1**]^+^/pyridyl base to form **4**. Compound **4** is then converted by reaction with the hydridophilic Lewis acid [**1**]^+^, ultimately to form [**2**]^+^. To preclude any hydride‐transfer processes mediated by Lewis acidic boranes derived from decomposition of [BArCl],^17^ we utilized a different anion. An equimolar mixture of **4** and [TBP−H][AlCl_4_] also led to the formation of the hydrocarbation product [**2**][AlCl_4_] upon heating at 60 °C, thus confirming that these conversions are not borane‐mediated.

Having identified **4** as a viable intermediate in the hydrocarbation pathway, we focused our attention on the details of the hydride‐transfer/protonation steps that convert **4** into [**2**]^+^. The formation of [**2**]^+^ could proceed from **4** by concerted protonation/1,2‐hydride transfer (analogous to pathway B, Scheme 1). However, when the migrating hydrogen atom in **4** was replaced with deuterium (Scheme 6), the deuterium atom was transferred from position 1 to position 3, thus precluding a 1,2‐hydride‐transfer mechanism. Furthermore, a related mechanism involving protonation at position 2 and an intramolecular 1,3‐hydride shift is disfavored, as it would proceed via the original vinyl cation formed on the combination of [**1**]^+^ and the alkyne (Scheme 4, left) and thus would be expected to proceed in the absence of the pyridyl base, which was not observed. Compound **4** was not observed in situ on treating 4‐ethynylanisole with the FLP 2,6‐lutidine/[**1**]^+^, thus indicating that it is rapidly consumed, presumably by reaction with [**1**]^+^ as discussed above. This reaction would generate an equivalent of [**5**]^+^ and **1‐H**. The chemical structure of [**5**]^+^ contains an α,β,γ,δ‐unsaturated system and is a Michael acceptor. Therefore, it is feasible that **1‐H** would transfer a hydride to the δ position of [**5**]^+^, which would be consistent with the deuterium‐labeling studies and represent an intermolecular hydride‐shuttle route for the 1,3‐hydride migration. To gain insight into this hypothesis, the hydride‐ion affinity (HIA) of [**5**]^+^ (relative to Et_3_B) was computationally determined (at the M06‐2X/6‐311G(d,p) level (DCM solvation (PCM)). The HIA of [**5**]^+^ at the β position (−47 kcal mol^−1^) is lower than that of [**1**]^+^ (−53 kcal mol^−1^), thus confirming that intermolecular hydride transfer from **4** to [**1**]^+^ is favored, consistent with the formation of **1‐H** observed during the FLP reaction. More notably, the HIA of [**5**]^+^ at the δ position is higher than that at the β position and higher than that of [**1**]^+^. This result indicates that the hydride may be transferred from **4** to the δ position of **5** (to yield allene tautomer **6**, Scheme 7, inset) in a process mediated by [**1**]^+^ (as the direct conversion of **4** into **6** does not proceed). An analogous transformation was reported for the acid‐catalyzed rearrangement of tertiary propargyl alcohols to allenenols (known as the Meyer–Schuster rearrangement).^18^ Protonation of **6** would then lead to the observed product, [**2**]^+^.

**Scheme 7:**
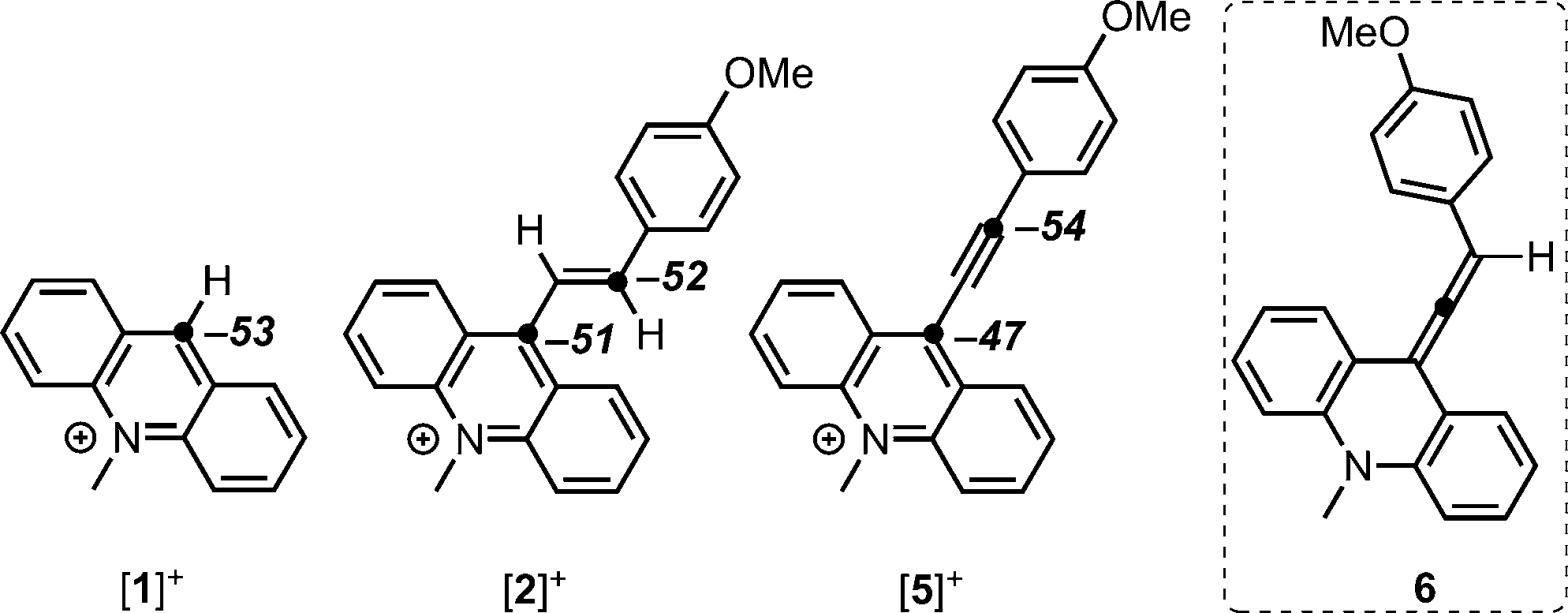
Hydride‐ion affinities of [**1**]^+^, [**2**]^+^, and [**5**]^+^, and structure of compound **6**.

To assess experimentally the reactivity of [**5**]^+^, we heated equimolar amounts of [**5**][I] and *N*‐methylacridane (**1‐H**), both independently synthesized, in solution at 60 °C. Interestingly, no formation of the expected product **6** from hydride transfer was detected. Instead, after 30 h, the major product observed was the alkene [**7**]^+^ (confirmed crystallographically), which was presumably formed by the reaction of allene **6** with the by‐product [**1**]^+^ from hydride transfer (Scheme 8). Characterization of [**7**]^+^ enabled the minor species observed in the formation of [**2**]^+^/[**3**]^+^ from the reaction of [**1**]^+^/2,6‐lutidine/4‐ethynylanisole to be identified as [**7**]^+^. This result supports the intermediacy of allene **6** in the FLP reaction to form [**2**]^+^/[**3**]^+^ and also indicates that the protonation of **6** is favored over its alkylation. The latter hypothesis was confirmed by the addition of one equivalent of [2,6‐lutidinium][AlCl_4_] to [**7**][I], which led to the hydrocarbation product [**2**]^+^ and [**1**]^+^. The conversion of [**7**]^+^ into [**2**]^+^ also suggests the reversibility of C−C bond formation involving allene **6** and [**1**]^+^.

**Scheme 8:**
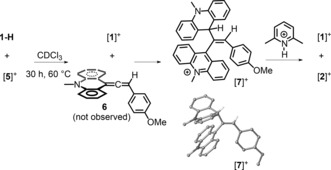
Synthesis of the 1,2‐hydrocarbation product [**7**][BArCl], and solid‐state structure of [**7**]^+^.

The hydrocarbation product [**2**]^+^ is also a Michael acceptor, so could undergo further reduction initiated by hydride transfer from **1‐H**. However, this reactivity was not observed, potentially because [**1**]^+^ has a higher HIA than [**3**]^+^ (HIA=−51 kcal mol^−1^ in the β and −52 kcal mol^−1^ in the δ position). The lower electrophilicity in the δ position of [**2**]^+^ relative to that in [**5**]^+^ is attributed to the reduced conjugation between the γ,δ system and the acridinyl moiety (in the solid‐state structure of [**2**]^+^, the torsion angle Cα,Cβ,Cγ,C*δ* is 46.0°, whereas it would be 0° in [**5**]^+^). Combined, the data indicate that this 1,2‐hydrocarbation reaction is the result of an initial FLP‐type process: Lewis acid activation of the alkyne/deprotonation (dehydrocarbation) to form **4**, followed by stepwise intermolecular hydride transfer/protonation steps to yield the final product [**2**]^+^ or [**3**]^+^, with [**1**]^+^ acting as an exogenous Lewis acid to facilitate hydride shuttling (Scheme 9). The computationally optimized structure of allene **6** reveals that protonation at the least hindered face of the allene would lead to the *Z* isomer [**3**]^+^, which is consistent with the observation that [**3**]^+^ is the kinetic product and is formed at 20 °C. We hypothesize that owing to greater unfavorable steric interactions of the substituents in the *Z* isomer [**3**]^+^, heating is sufficient to form predominately the thermodynamic *E* isomer [**2**]^+^ (possibly by reversible protonation/deprotonation of [**3**]^+^). Similar reactivity has been observed for the protonation of allenolates, with the *Z* isomer being the kinetic product and the *E* isomer the thermodynamic product.^19^

**Scheme 9:**
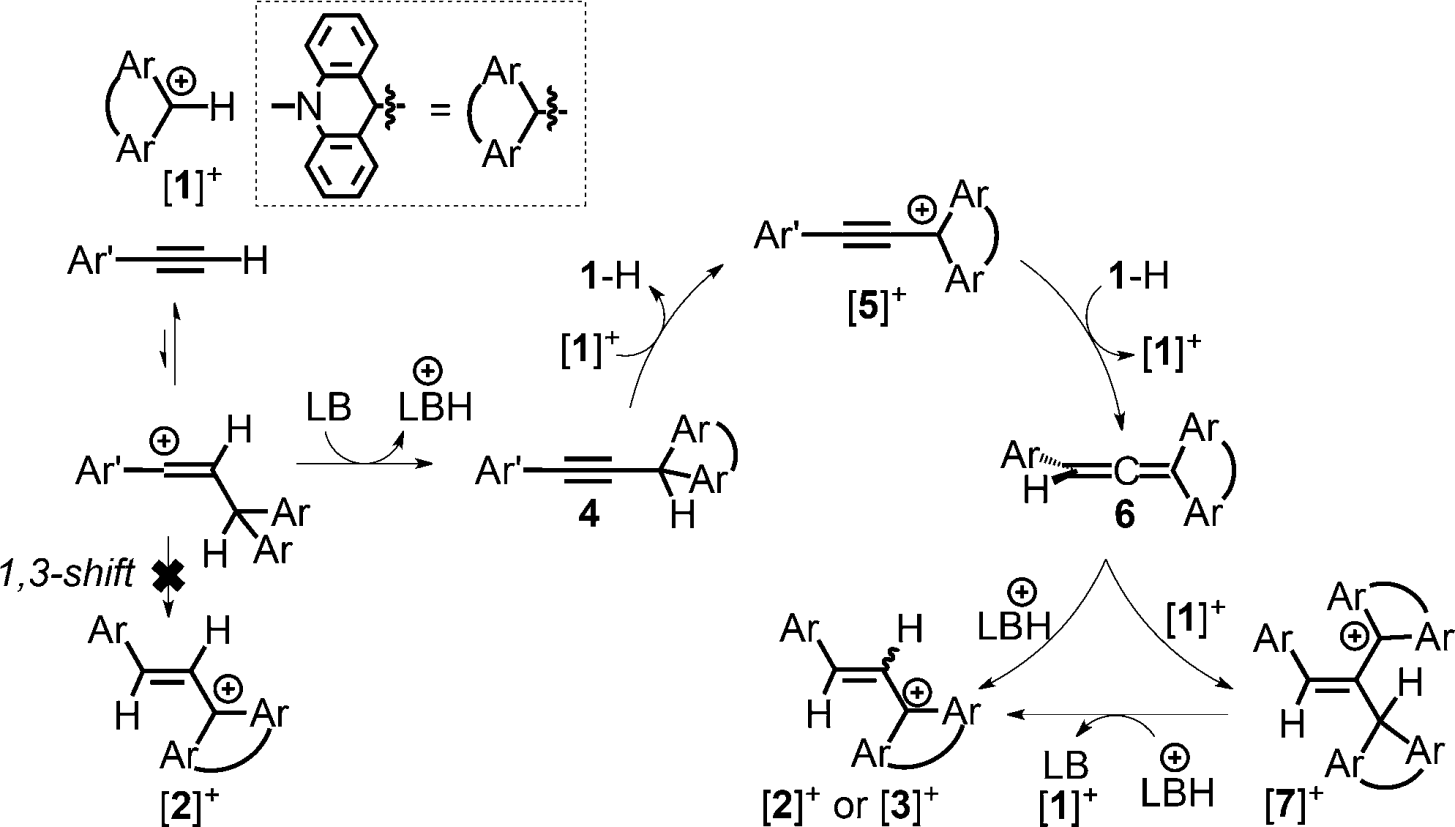
Proposed mechanism for the formation of the 1,2‐hydrocarbation products [**2**]^+^/[**3**]^+^/[**7**]^+^.

The rate‐determining step of the overall reaction appears to be the dehydrocarbation of the alkyne with the Lewis acid/Lewis base FLP. This hypothesis was supported by the observation of minimal reactivity of the carbon Lewis acid based FLP combination after 72 h at 60 °C when 4‐ethynylanisole was replaced with the less nucleophilic alkyne 4‐ethynyltoluene (Scheme 10, left). However, when independently synthesized **8** was heated in the presence of the 2,6‐lutidinium cation and [**1**]^+^ (10 mol %) to facilitate hydride transfer, the hydrocarbation product [**9**]^+^ was observed as the major product. Notably, the conversion of **8** into [**9**]^+^ was significantly quicker when [**1**]^+^ was used as an exogenous hydridophilic Lewis acid to mediate the 1,3‐hydride migration than in the absence of [**1**]^+^, as observed in the formation of [**2**]^+^ from **4**.

**Scheme 10:**
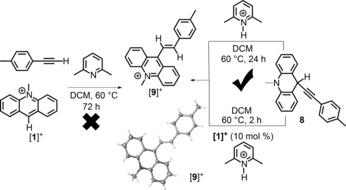
Synthesis of the 1,2‐hydrocarbation product [**9**]^+^ from **8** and [2,6‐lutidinium], and solid state structure of [**9**]^+^.

In summary, the first example of alkyne 1,2‐hydrocarbation enabled by FLP chemistry has been presented. It is based on extending an established FLP reaction, alkyne activation/deprotonation, to carbon Lewis acid based FLPs, which in this case results in dehydrocarbation. This step is then followed by intermolecular hydride transfer enabled by the Lewis acidic component of the FLP, which acts as a hydride shuttle. Finally, protonation delivers the hydrocarbation product. A series of control experiments disfavored a mechanism based on intramolecular 1,3‐hydride migration; instead, a new alkyne 1,2‐hydrocarbation mechanism was identified that requires a Lewis base and a free Lewis acid. The ability to circumvent the intramolecular 1,3‐hydride migration step in this hydrocarbation mechanism is particularly important, since 1,3‐hydride migration is a slow process (even when highly exothermic), as highlighted by Mayr and co‐workers.^6^ Therefore, in previous studies slow intramolecular 1,3‐hydride migration led to the carbocation derived from the addition of [R_2_CH]^+^ to an alkyne/olefin reacting with an external nucleophile and not undergoing hydrocarbation.^20^ The intermolecular hydride‐shuttle mechanism disclosed herein offers the potential to circumvent the intramolecular 1,3‐hydride‐migration step and make hydrocarbation reactions more general.

## Conflict of interest

The authors declare no conflict of interest.

## Supporting information

As a service to our authors and readers, this journal provides supporting information supplied by the authors. Such materials are peer reviewed and may be re‐organized for online delivery, but are not copy‐edited or typeset. Technical support issues arising from supporting information (other than missing files) should be addressed to the authors.

Supplementary
