# Supplementary material for: Frustrated Lewis Pair Mediated 1,2‐Hydrocarbation of Alkynes
Source: Angew Chem Int Ed Engl. 2017 Jul 4;56(31):9202–6. doi: 10.1002/anie.201705100 (PMC5577509; doi:10.1002/anie.201705100)
Supplement: Supplementary file 1 — Supplementary [file ANIE-56-9202-s001.pdf]

Angewandte Chemie
Eine Zeitschrift der Gesellschaft Deutscher Chemiker

Supporting Information

Frustrated Lewis Pair Mediated 1,2-Hydrocarbation of Alkynes

*Valerio Fasano, Liam D. Curless, James E. Radcliffe, and Michael J. Ingleson**

anie_201705100_sm_miscellaneous_information.pdf

Supporting Information

Table of Contents

1. General Remarks S2

2. Synthesis of compounds [**2**][BArCl] and [**3**][BArCl] S3

3. Attempted 1,2-hydrocarbation in absence of Lewis base S8

4. Synthesis and reactivity of the intermediates **4** and **4D** with protonated bases S9

4.1 Synthesis of the intermediate **4** and **4D** S9

4.2 Synthesis of the protonated bases S10

4.3 Reaction between **4** and [TBP-H][BArCl] with catalytic [**1**][BArCl] S12

4.4 Reaction between **4** and [TBP-H][BArCl] S13

4.5 Reaction between **4** and [TBP-H][$AlCl_4$] S15

4.6 Reaction between **4D** and [2,6-lutidinium][$AlCl_4$] S16

5. [**7**][I] synthesis S19

5.1 Synthesis of **1-H** S19

5.2 Reduction of [**5**][I] with **1-H** to form [**7**][I] S20

6. Synthesis and reactivity of the intermediates **4'** with [2,6-lutidinium][$AlCl_4$] S30

6.1 Synthesis of the intermediate **4'** S30

6.2 Reaction between **4'** and [2,6-lutidinium][$AlCl_4$] S32

6.3 Reaction between **4'** and [2,6-lutidinium][$AlCl_4$] with catalytic [**1**][BArCl] S35

7. HIA calculation coordinates of the Lewis acids S36

8. Crystal structures of [**2**]$^+$, [**3**]$^+$, [**7**]$^+$, [**9**]$^+$ S45

9. References S50

10. NMR spectra of al compounds S51

1. General Remarks

Unless otherwise indicated all manipulations were conducted under inert nitrogen atmosphere. 4-ethynylanisole, 4-ethynyltoluene, 2,6-lutidine, 2,4,6-tri-tert-butyl-pyridine were purchased from commercial sources and used as received unless otherwise stated. *N*-methyl- acridinium tetra(3,5-dichlorophenyl)borate ([**1**][BArCl]) was synthesized in accordance with the literature.[1] Dichloromethane, d_2-dichloromethane, *d*-chloroform were dried over CaH_2 and distilled and degassed before use. Solvents for column chromatography were of technical grade and used without further purification. Column chromatography was performed on silica gel (230-400 mesh). NMR spectra were recorded with a Bruker AV-400 spectrometer (400 MHz ^{1}H; 100 MHz ^{13}C; 128 MHz ^{11}B; 104 MHz ^{27}Al). ^{1}H NMR chemical shifts are reported in ppm relative to *protio* impurities in the deuterated solvents and ^{13}C NMR chemical shifts using the solvent resonances unless otherwise stated. ^{11}B NMR spectra were referenced to external BF_3:Et_2O, ^{27}Al to $Al(NO_3)_3$ in D_2O ($Al(D_2O)_6^{3+}$). Coupling constants *J* are given in Hertz (Hz), while the multiplicity of the signals are indicated as "s", "d", "t" "pent", "sept" or "m" for singlet, doublet, triplet, pentet, septet or multiplet, respectively. Mesitylene (distilled from K) was used as an internal standard to determine the NMR yields. GC-MS analysis was performed on an Agilent Technologies 7890A GC system equipped with an Agilent Technologies 5975C inert XL EI/CI MSD with triple axis detector. The column employed was an Agilent J&W HP-5ms ((5%-Phenyl)-methylpolysiloxane) of dimensions: length, 30 m; internal diameter, 0.250 mm; film, 0.25 μm. Mass spectra were recorded on a Waters QTOF mass spectrometer.

2. Synthesis of compounds [2][BArCl] and [3][BArCl]

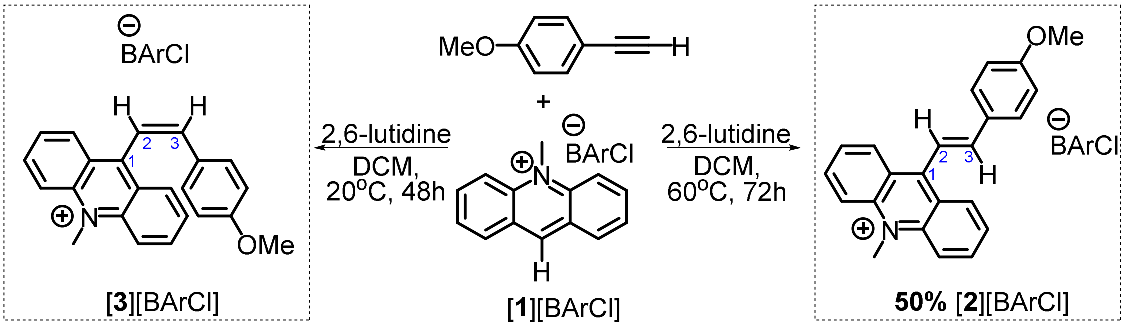

In a J Youngs NMR tube, 4-ethynylanisole (13 mg, 0.1 mmol, 1.0 eq.) was added to a solution of a *N*-methyl- acridinium tetra(3,5-dichlorophenyl)borate ([**1**][BArCl]) (79 mg, 0.1 mmol, 1.0 eq.) and 2,6-lutidine (12 µL, 0.1 mmol, 1.0 eq.) in anhydrous d_2-DCM (0.8 mL). The NMR tube was sealed and monitored by multinuclear NMR spectroscopy periodically while heating at 60°C which resulted in a colour change from yellow to red. After 72 h, full consumption of [**1**][BArCl] was observed. Subsequent addition of mesitylene (0.1 mmol) to the reaction mixture allowed the determination of the NMR yields based on the relative integral of the *N*-methyl signal of [**2**][BArCl] (50% NMR yield). ^{1}H NMR (400 MHz, CD_2Cl_2) δ 8.71 (dd, *J* = 8.8 Hz, *J* = 1.0 Hz, 2H), 8.18 (ddd, *J* = 9.1 Hz, *J* = 6.8 Hz, *J* = 1.0 Hz, 2H), 8.04 (d, *J* = 9.1 Hz, 2H), 8.01 (d, *J* = 15.9 Hz, 1H vinylC), 7.82 (ddd, *J* = 8.6 Hz, *J* = 6.8 Hz, *J* = 1.0 Hz, 2H), 7.73-7.70 (m, 2H), 7.27 (d, *J* = 16.1 Hz, 1H vinylC), 7.07 (q, *J* = 2.0 Hz, 8H), 7.06 (d, *J* = 9.1 Hz, 2H), 6.88 (t, *J* = 2.0 Hz, 4H), 4.38 (s, 3H), 3.91 (s, 3H) ppm. ^{13}C{^{1}H} NMR (100 MHz, CD_2Cl_2) δ 165.9 (q, $J_{B\text{-}C}$ = 49.4 Hz), 162.9, 159.7, 141.4, 139.3, 133.6, 133.5-133.3(m) 130.7, 129.9, 128.4, 128.0, 125.2, 123.5, 122.8, 117.8, 117.4, 115.3, 56.1, 38.3 ppm. ^{11}B NMR (128 MHz, CD_2Cl_2) δ -6.9 ppm. MS: m/z calcd for $C_{23}H_{20}NO^+$ [**2**]$^+$ 326.1 Found ES$^+$ 326.1. m/z calcd for $C_{24}H_{12}BCl_8^+$ [BArCl] 594.8 Found ES$^+$ 595.0. Accurate mass for [**2**]$^+$ 326.1545 Found 326.1561. Elemental Analysis for [**2**][BArCl] Expected C 61.28%, H 3.50%, N 1.52%. Found: C 58.85%, H 3.55%, N 1.91%.

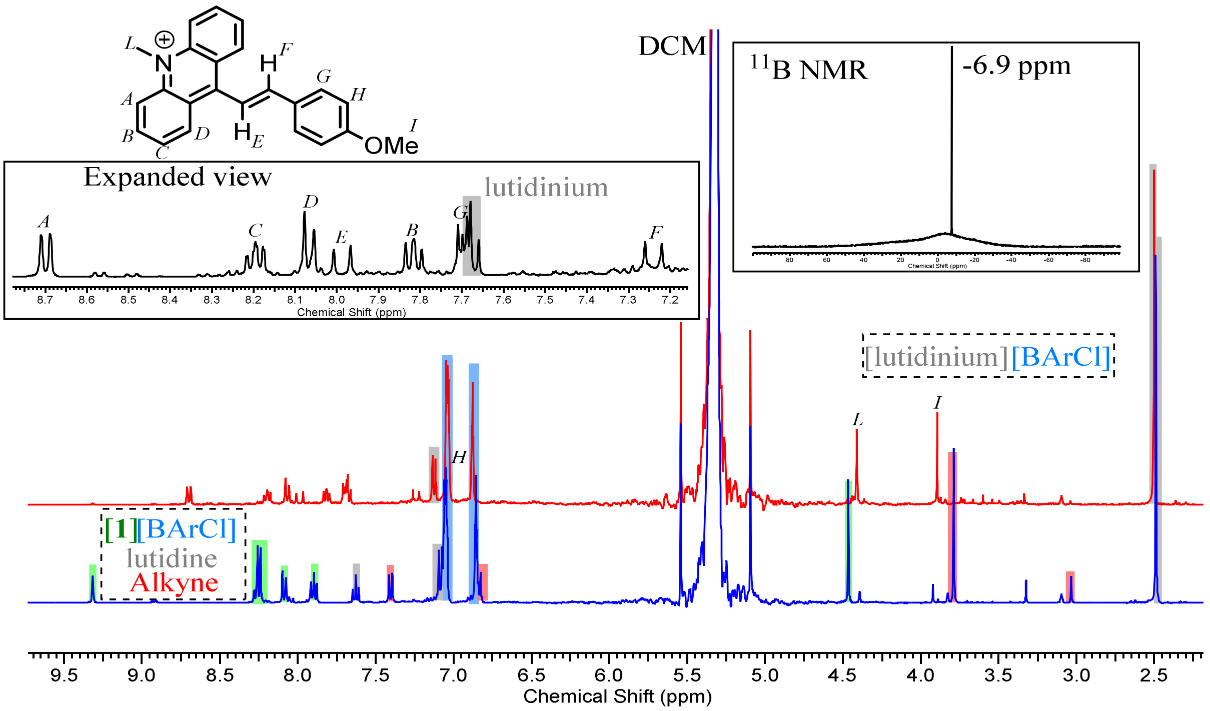

Figure S1. *In situ* ^{1}H-NMR spectra of the reaction with 4-ethynylanisole, [**1**][BArCl] and 2,6-lutidine in anhydrous DCM, with a d_6-DMSO capillary inserted. Blue (t = 5 min, r.t.), red (after 72 h at 60°C). Inset, ^{11}B-NMR spectrum after 72 h at 60°C.

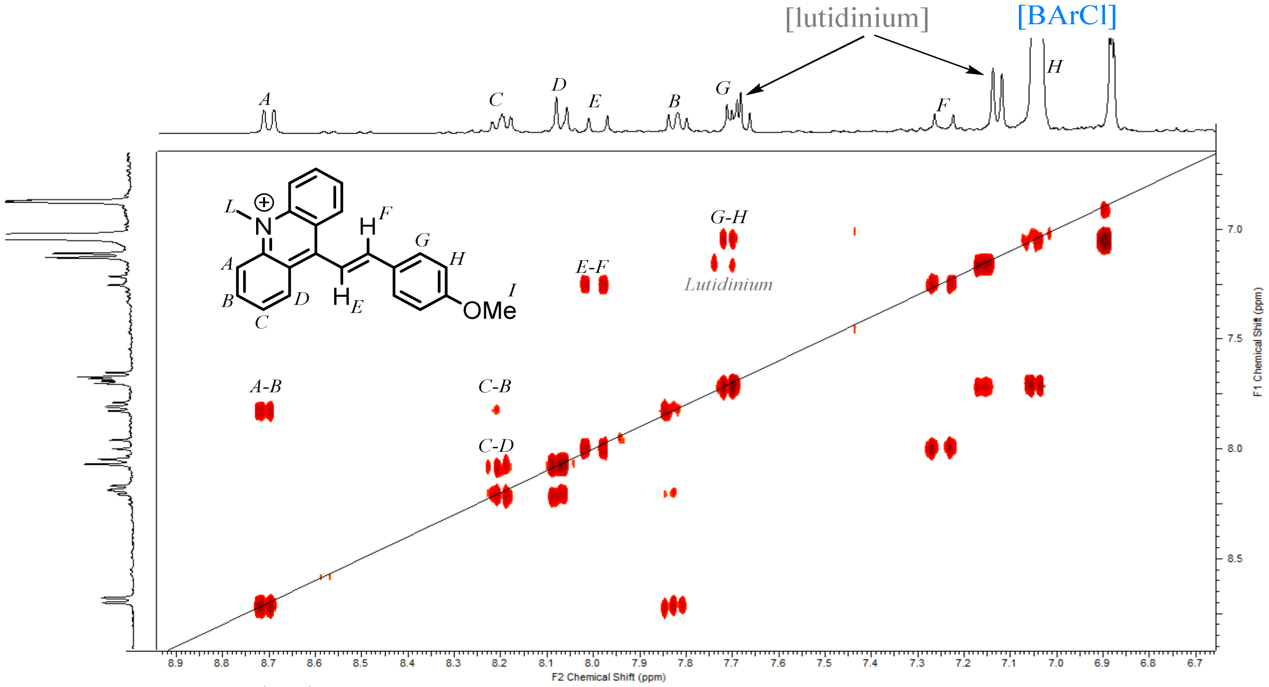

Figure S2. *In situ* ^{1}H-^{1}H COSY NMR spectra of the reaction with 4-ethynylanisole, [**1**][BArCl] and 2,6-lutidine in anhydrous DCM, with a d_6-DMSO capillary inserted, after 72 hours at 60°C.

Single crystals of [**2**][BArCl] were isolated from the dichloromethane solution layered with hexane.

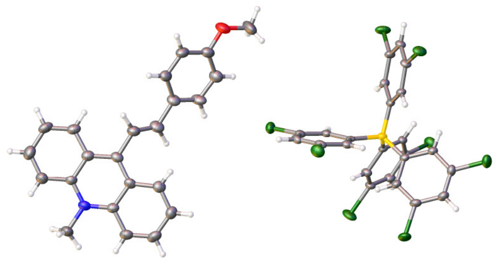

The product [**2**][BArCl] was again isolated and characterized by X-ray diffraction when 2,6-lutidine was replaced with 2,4,6-tri-tert-butyl-pyridine (25 mg, 0.1 mmol, 1.0 eq.).

The product [**3**][BArCl] was instead obtained when a J Youngs NMR tube containing 4-ethynylanisole (13 mg, 0.1 mmol, 1.0 eq.), [**1**][BArCl] (79 mg, 0.1 mmol, 1.0 eq.) and 2,6-lutidine (12 µL, 0.1 mmol, 1.0 eq.) in anhydrous d_2-DCM (0.8 mL) was inverted for 48 hours at room temperature. ^{1}H NMR spectroscopy revealed partial consumption of [**1**] / alkyne with the formation of protonated lutidine along with a mixture of products.

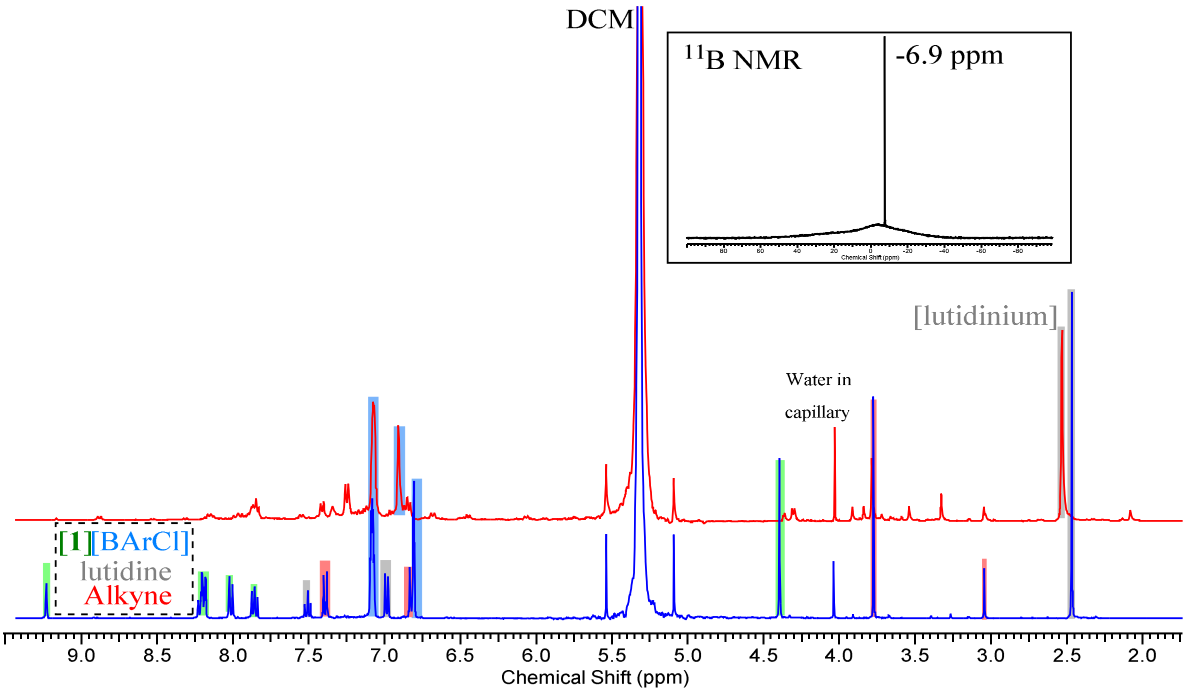

Figure S3. *In situ* ^{1}H-NMR spectra of the reaction with 4-ethynylanisole, [**1**][BArCl] and 2,6-lutidine in anhydrous DCM, with a d_6-DMSO capillary inserted. Blue (t = 5 min, r.t.), red (after 48 h at r.t.). Inset, ^{11}B-NMR spectrum after 48 h at r.t..

On layering the solution with pentane, suitable crystals were obtained and analysed by X-ray crystallography, revealing the formation of [**3**][BArCl].

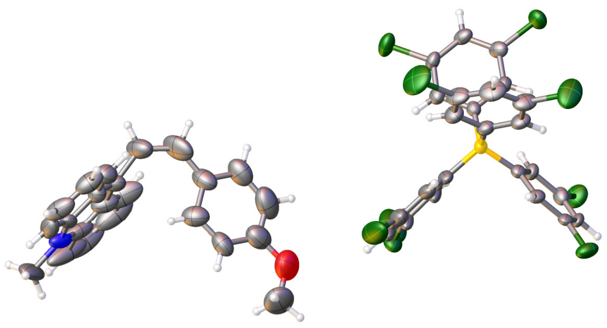

3. Attempted 1,2-hydrocarbation in absence of Lewis base

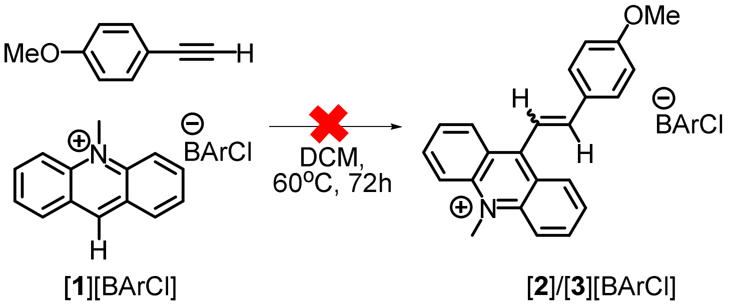

In a J Youngs NMR tube, 4-ethynylanisole (13 mg, 0.1 mmol, 1.0 eq.) was added to a solution of a *N*-methyl- acridinium tetra(3,5-dichlorophenyl)borate ([**1**][BArCl]) (79 mg, 0.1 mmol, 1.0 eq.) and 4-ethynylanisole (13 mg, 0.1 mmol, 1.0 eq.) in anhydrous d_2-DCM (0.8 mL). The NMR tube was sealed and monitored by multinuclear NMR spectroscopy periodically while heating at 60°C. After 72 h, no product was observed and [**1**][BArCl] was found unreacted. Instead, degradation of the alkyne to unidentified products was observed: this is tentatively attributed to Lewis acid catalysed oligomerisation of the alkyne at elevated temperatures.

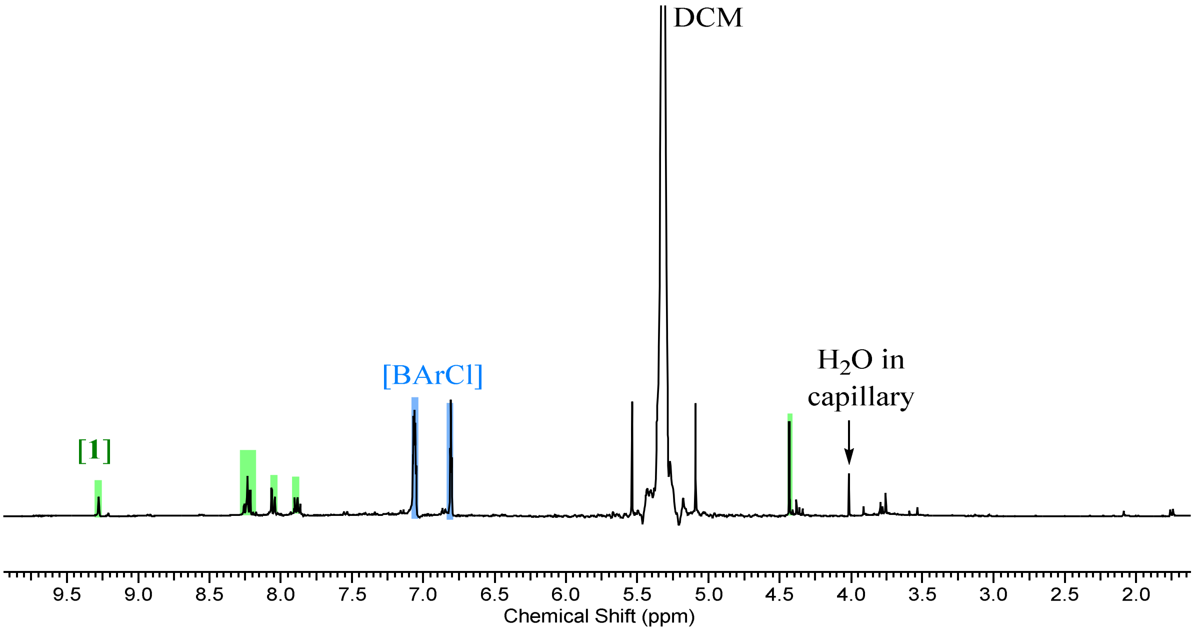

Figure S4. *In situ* ^{1}H-NMR spectrum of the reaction with 4-ethynylanisole and [**1**][BArCl] in anhydrous DCM, with a d_6-DMSO capillary inserted, after 72 h at 60°C.

4. Synthesis and reactivity of the intermediate 4 and 4D with protonated bases

4.1 Synthesis of the intermediate 4 and 4D

- Synthesis of 9-((4-methoxyphenyl)ethynyl)acridine

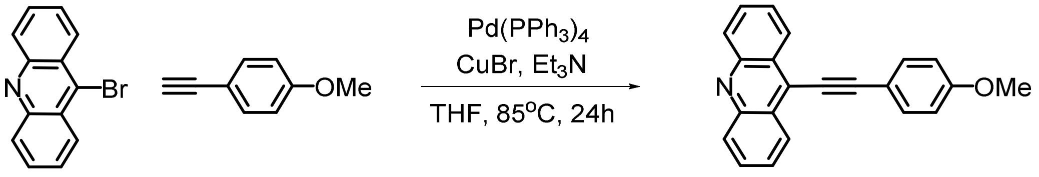

A Schlenk tube was charged with tetrakis-(triphenylphosphine)palladium(0) (88 mg, 0.075 mmol, 0.02 eq.) and copper(I) bromide (22 mg, 0.150 mmol, 0.04 eq.). Tetrahydrofuran was added and the suspension was stirred for five minutes, followed by the addition of 9-bromoacridine (1g, 3.750 mmol, 1 eq.) and triethylamine (6 mL). After five minutes under stirring, 4-ethynylanisole (551 µL, 4.125 mmol, 1.1 eq.) was added and the solution was stirred and refluxed at 85°C for 24 h. The solution was cooled to room temperature, filtered through celite/silica and eluted with diethyl ether (3 x 10 mL). The solvent was removed in vacuo and the crude material was purified via column chromatography (petr. ether: AcOEt 80:20) yielding the corresponding product as a yellow solid (985 mg, 3.184 mmol, 85%). $R_f = 0.2$. ^{1}H NMR (400 MHz, $CDCl_3$) δ 8.58 (d, *J* = 8.0 Hz, 2H), 8.25 (d, *J* = 8.8 Hz, 2H), 7.82 (t, *J* = 8.0 Hz, 2H), 7.75 (d, *J* = 8.8 Hz, 2H), 7.64 (t, *J* = 8.5 Hz, 2H), 7.01 (d, *J* = 8.8 Hz, 2H), 3.91 (s, 3H) ppm. ^{13}C{^{1}H} NMR (100 MHz, $CDCl_3$): δ 160.7, 148.6, 133.7, 130.3, 129.9, 128.4, 126.8, 126.4, 126.3, 114.5, 114.4, 105.7, 83.2, 55.5 ppm. GC-MS: m/z calculated for $C_{22}H_{15}NO$, 309.1; found 309.1 (retention time of analyte: 25.84 minutes). Accurate mass: $[MH]^+$ 310.1126; found: 310.1125.

- Synthesis of *N*-methyl-9-((4-methoxyphenyl)ethynyl)acridium iodide ([5][I])

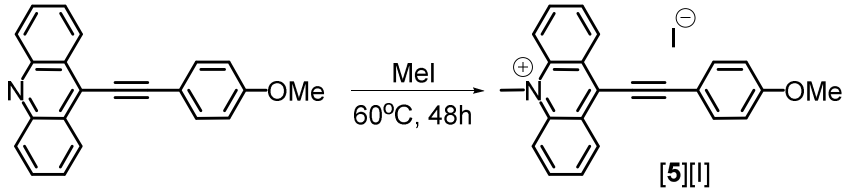

9-((4-methoxyphenyl)ethynyl)acridine (500 mg, 1.570 mmol, 1.0 eq.) was dissolved in an excess of iodomethane (1.5 mL, > 10.0 eq.), within a sealed ampule fitted with a J. Young's stopcock. The reaction vessel was heated at 60°C for 2 days and then iodomethane was removed under vacuum, leaving a dark red solid. The latter was washed with hexane affording *N*-methyl-9-((4-methoxyphenyl)ethynyl)acridium iodide [**5**][I] as a red-brown solid (657 mg, 1.456 mmol, 93%). ^{1}H NMR (400 MHz, $CDCl_3$) δ 8.85 (d, *J* = 8.5 Hz, 2H), 8.80 (d, *J* = 9.3 Hz, 2H), 8.39 (t, *J* = 8.3 Hz, 2H), 7.98 (t, *J* = 8.3 Hz, 2H), 7.89 (d, *J* = 8.8 Hz, 2H), 7.09 (d, *J* = 8.8 Hz, 2H), 5.11 (s, 3H), 3.96 (s, 3H) ppm. ^{13}C{^{1}H} NMR (100 MHz, $CDCl_3$): δ 163.1, 142.3, 141.0, 139.0, 135.5, 129.6, 128.3, 126.3, 119.9, 119.6, 115.1, 111.9, 84.9, 55.8, 41.1 ppm. MS: m/z calcd for $C_{23}H_{18}NO^+$ [**5**]$^+$ 324.4 Found ES^+ 324.6, m/z calcd for I^- 126.9 Found ES^- 126.8. Accurate mass for $C_{23}H_{18}NO^+$ [**5**]$^+$ 324.1383 Found 324.1371.

- Synthesis of *N*-methyl-9-((4-methoxyphenyl)ethynyl)acridane (4)

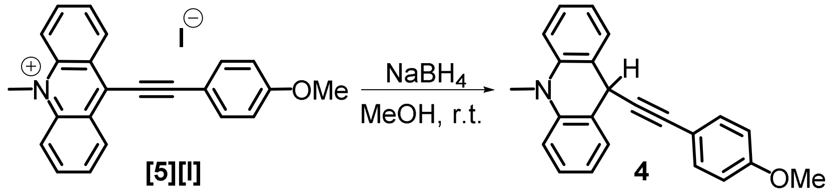

N-methyl-9-((4-methoxyphenyl)ethynyl)acridinium iodide [**5**][I] (25 mg, 0.055 mmol, 1.0 eq.) was dissolved in MeOH (2 mL), obtaining a dark red solution. While under stirring, $NaBH_4$ (3 mg, 0.080 mmol, 1.5 eq.) was added at room temperature. After a few seconds, the solution became green, and then an excess of water was added to quench the reaction. The solution was extracted two times with Et_2O (3 mL) and the organic phase was dried over anhydrous $MgSO_4$. After filtration, the residue was purified on a plug of silica, obtaining the desired compound **4** as a yellow oil (10 mg, 0.031 mmol, 56%). ^1H NMR (400 MHz, $CDCl_3$) δ 7.76 (d, *J* = 7.5 Hz, 2H), 7.54 (d, *J* = 8.8 Hz, 2H), 7.29 (t, *J* = 8.0 Hz, 2H), 7.06 (t, *J* = 7.5 Hz, 2H), 6.96 (d, *J* = 8.3 Hz, 2H), 6.91 (d, *J* = 8.8 Hz, 2H), 4.94 (s, 1H), 3.85 (s, 3H), 3.46 (s, 3H) ppm. $^{13}C\{^1H\}$ NMR (100 MHz, $CDCl_3$): δ 159.5, 142.9, 133.2, 127.4, 126.4, 124.2, 120.9, 115.6, 113.9, 111.9, 87.2, 85.3, 55.3, 35.2, 33.3 ppm. *Note*: the compound was found to be unstable on flash chromatography, so it was used as obtained after filtration on a plug of silica.

- Synthesis of *N*-methyl-9-((4-methoxyphenyl)ethynyl)acridane (4D)

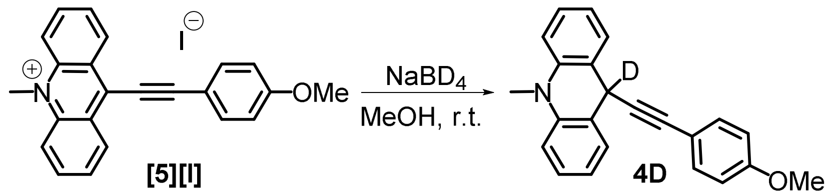

N-methyl-9-((4-methoxyphenyl)ethynyl)acridinium iodide [**5**][I] (25 mg, 0.055 mmol, 1.0 eq.) was dissolved in MeOH (2 mL), obtaining a dark red solution. While under stirring, $NaBD_4$ (3 mg, 0.072 mmol, 1.3 eq.) was added at room temperature. After a few seconds, the solution became green, and then an excess of water was added to quench the reaction. The solution was extracted two times with Et_2O (3 mL) and the organic phase was dried over anhydrous $MgSO_4$. After filtration, the residue was purified on a plug of silica, obtaining the desired compound as a yellow oil (16 mg, 0.049 mmol, 89%). ^1H NMR (400 MHz, CD_2Cl_2) δ 7.69 (d, *J* = 7.6 Hz, 2H), 7.51 (d, *J* = 8.4 Hz, 2H), 7.29 (t, *J* = 7.6 Hz, 2H), 7.05 (t, *J* = 7.6 Hz, 2H), 6.97 (d, *J* = 7.6 Hz, 2H), 6.90 (d, *J* = 9.1 Hz, 2H), 3.82 (s, 3H), 3.44 (s, 3H) ppm. $^{13}C\{^1H\}$ NMR (100 MHz, CD_2Cl_2): δ 160.2, 143.5, 133.7, 128.0, 127.0, 124.6, 121.4, 115.9, 114.5, 112.6, 87.1, 86.2, 55.9, 35.2 (t, *J* = 19 Hz), 33.8 ppm. ^2H NMR (61 MHz, CH_2Cl_2) δ 4.95 ppm (s, 1D).

Note: the compound was found instable on flash chromatography, so it was used as obtained after filtration on a plug of silica.

4.2 Synthesis of protonated bases

- 2,4,6-tri-*tert*-butylpyridinium chloride ([TBP-H][Cl])

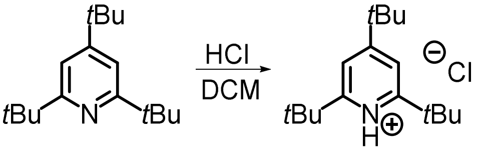

Under nitrogen atmosphere, 2,4,6-tri-*tert*-butylpyridine (500 mg, 2.00 mmol, 1.0 eq.) was dissolved in DCM (2 mL), followed by the addition of a 4M solution of HCl in dioxane (0.5 mL, 2.00 mmol, 1.0 eq.). After stirring for 30 min at r.t., the mixture was dried under vacuum, obtaining 2,4,6-tri-*tert*-butylpyridinium chloride as a white solid (423 mg, 1.49 mmol, 75%). ^{1}H NMR (400 MHz, CH_2Cl_2) δ 14.55 (bs, 1H), 7.54 (s, 2H), 1.72 (s, 18H), 1.38 (s, 9H) ppm.

- 2,4,6-tri-*tert*-butylpyridinium tetra(3,5-dichlorophenyl)borate ([TBP-H][BArCl])

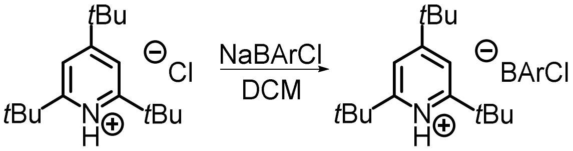

2,4,6-tri-*tert*-butylpyridinium chloride (50 mg, 0.17 mmol, 1.0 eq.) was dissolved in DCM (2 mL), followed by the addition of sodium tetra(3,5-dichlorophenyl)borate (NaBArCl, 119 mg, 0.19 mmol, 1.1 eq.). After stirring the reaction mixture for 12h at r.t., the system was filtered to remove NaCl and the residue solution was concentrated in vacuo, obtaining 2,4,6-tri-*tert*-butylpyridinium tetra(3,5-dichlorophenyl)borate as a white solid (15 mg, 0.02 mmol, 12%). ^{1}H NMR (400 MHz, $CDCl_3$) δ 10.56 (bs, 1H), 7.69 (s, 2H), 7.01 (m, 8H), 6.94 (t, J = 2.0 Hz, 4H), 1.44 (s, 18H), 1.40 (s, 9H) ppm. ^{11}B NMR (128 MHz, $CDCl_3$) δ -6.95 ppm (s, 1B).

- 2,4,6-tri-*tert*-butylpyridinium tetrachloroaluminate

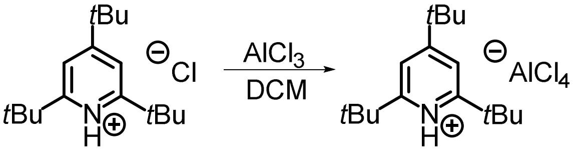

2,4,6-tri-*tert*-butylpyridinium chloride (50 mg, 0.17 mmol, 1.0 eq.) was dissolved in DCM (2 mL), followed by the addition of aluminium trichloride (23 mg, 0.17 mmol, 1.0 eq.). After stirring the reaction mixture for 30 min at r.t., the mixture was dried under vacuum, obtaining 2,4,6-tri-*tert*-butylpyridinium tetrachloroaluminate as a white solid (15 mg, 0.02 mmol, 12%). ^{1}H NMR (400 MHz, CH_2Cl_2) δ 10.65 (bs, 1H), 7.73 (s, 2H), 1.56 (s, 18H), 1.44 (s, 9H) ppm. ^{27}Al NMR (104 MHz, CH_2Cl_2) δ 103.02 ppm (s, 1Al).

- 2,6-lutidinium chloride

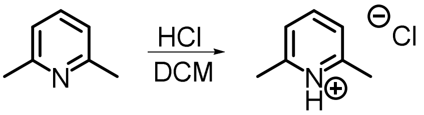

Under nitrogen atmosphere, 2,6-lutidine (353 µL, 3.00 mmol, 1.0 eq.) was dissolved in DCM (2 mL), followed by the addition of a 4M solution of HCl in dioxane (0.75 mL, 3.00 mmol, 1.0 eq.). After stirring for 30 min at r.t., the mixture was dried under vacuum, obtaining 2,6-lutidinium chloride as a white solid (393 m4.94, 2.74 mmol, 91%). ^{1}H NMR (400 MHz, CH_2Cl_2) δ 14.44 (bs, 1H), 8.06 (t, *J* = 7.8 Hz, 1H), 7.42 (d, *J* = 7.8 Hz, 2H), 2.91 (s, 6H) ppm.

- 2,6-lutidinium tetrachloroaluminate

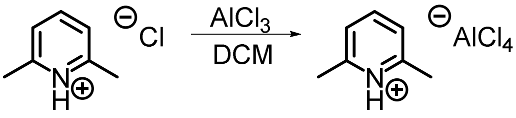

2,6-lutidinium chloride (100 mg, 0.689 mmol, 1.0 eq.) was dissolved in DCM (2 mL), followed by the addition of aluminium trichloride (92 mg, 0.689 mmol, 1.0 eq.). After stirring the reaction mixture for 30 min at r.t., the mixture was dried under vacuum, obtaining 2,6-lutidinium tetrachloroaluminate as a white solid (140 mg, 0.506 mmol, 74%). ^{1}H NMR (400 MHz, CH_2Cl_2) δ 14.64 (bs, 1H), 8.23 (t, *J* = 8.0 Hz, 1H), 7.56 (d, *J* = 8.0 Hz, 2H), 2.90 (s, 6H) ppm. ^{27}Al NMR (104 MHz CH_2Cl_2) δ 103.02 ppm (s, 1Al).

4.3 Reaction between 4 and [TBP-H][BArCl] with catalytic [1][BArCl]

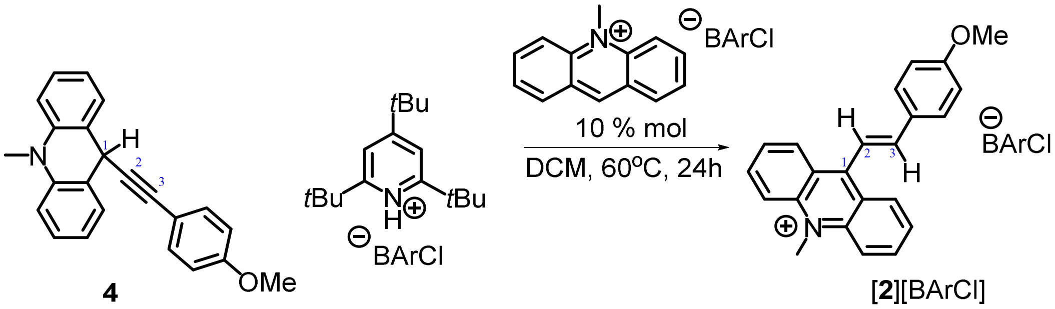

Under inert atmosphere, a J Youngs NMR tube was charged with **4** (10 mg, 0.030 mmol, 1.0 eq.) in d_2-DCM (0.5 mL), followed by the addition of [**1**][BArCl] (3 mg, 0.003 mmol, 0.1 eq.). On mixing, the solution changed from pale yellow to strong red. Then, 2,4,6-tri-*tert*-butylpyridinium tetra(3,5-dichlorophenyl)borate (26 mg, 0.030 mmol, 1.0 eq.) was added. The initial monitoring of the reaction by multinuclear NMR spectroscopy revealed conversion of $[\mathbf{1}]^+$ to **1-H**, along with a new *N*-methyl-acridinium resonance (pink signal in the following spectra). Upon heating at 60°C for 24 hours, the ^{1}H NMR spectrum revealed the disappearing of the starting material **4**, with concomitant formation of $[\mathbf{2}]^+$ (pair of doublets with J = 16 Hz) and 2,4,6-tri-*tert*-butylpyridine, along with partial regeneration of $[\mathbf{1}]^+$. MS: m/z calcd for $C_{23}H_{20}NO^+$ $[\mathbf{2}]^+$ 326.1 Found ES$^+$ 326.2. m/z calcd for $C_{24}H_{12}BCl_8^+$ [BArCl] 594.8 Found ES$^+$ 594.9.

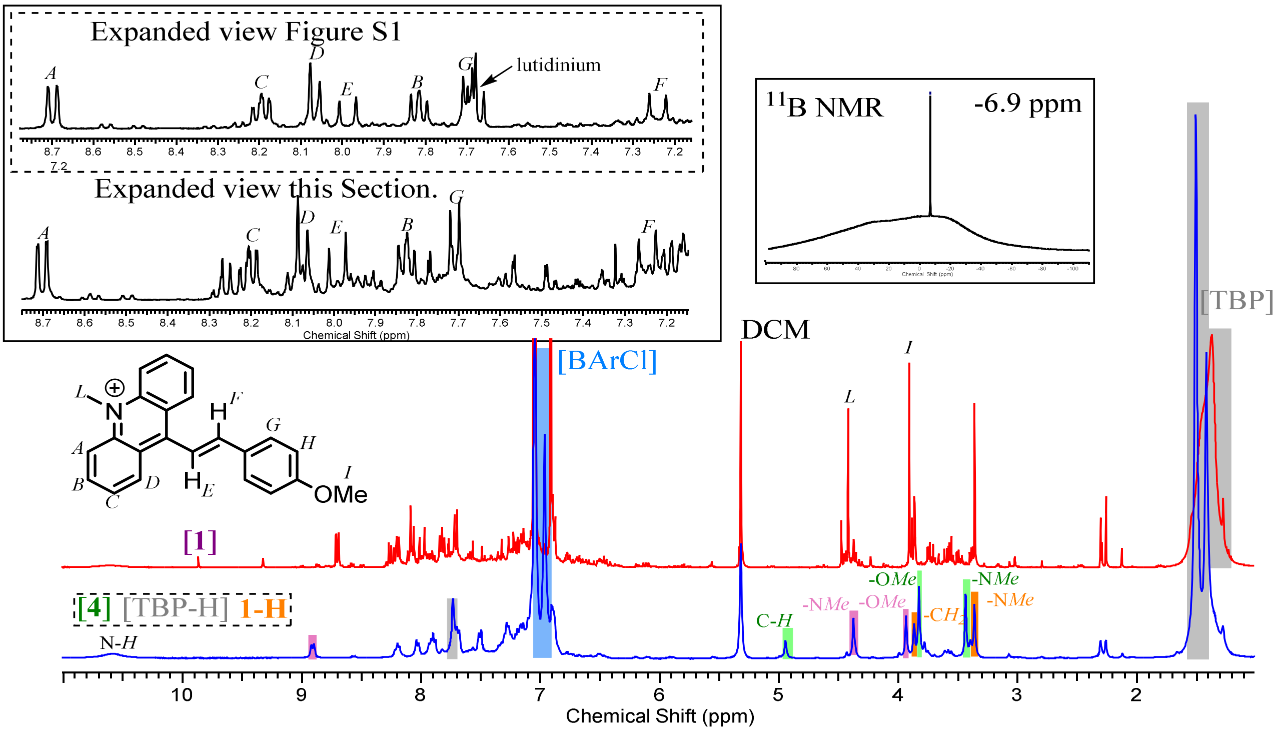

Figure S5. *In situ* ^{1}H-NMR spectra of the reaction with **4** and [2,4,6-tri-*tert*-butylpyridinium][BACl] with catalytic [**1**][BArCl] in anhydrous d_2-DCM. Blue (t = 5 min, r.t.), red (after 24 h at 60°C). Inset, ^{11}B-NMR spectrum after 24 h at 60°C. The expanded views show the comparison between the reaction after 24 h at 60°C (bottom) and the reaction reported in Section 2 (top).

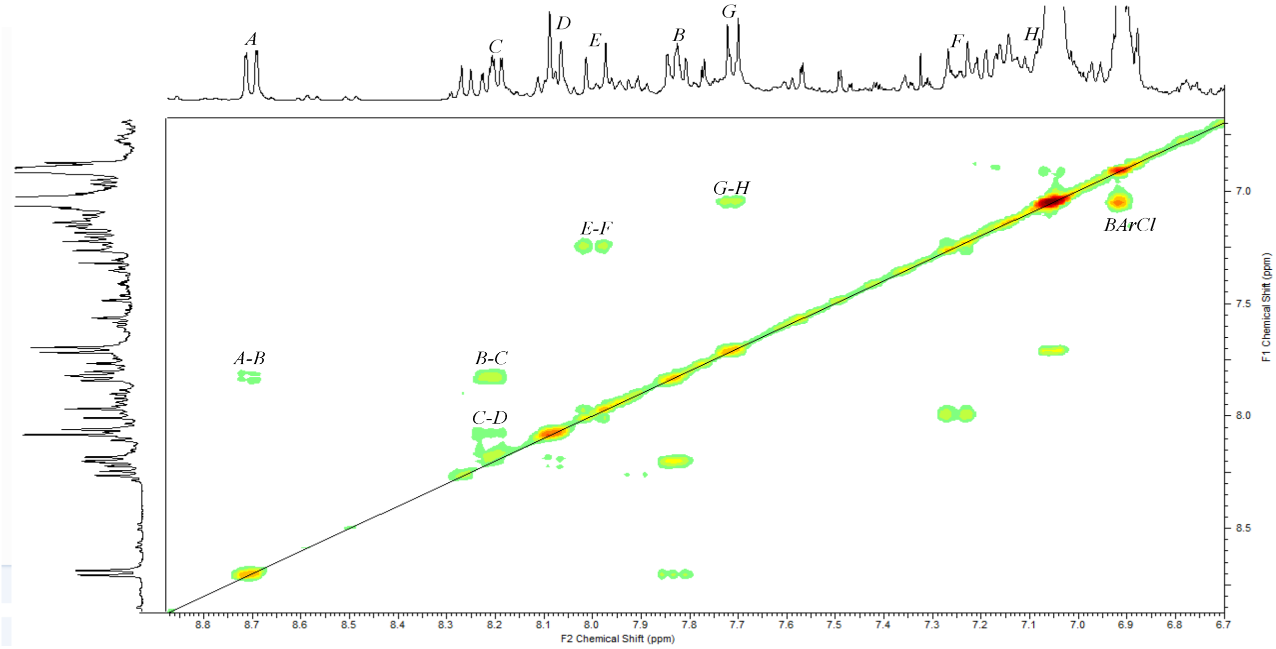

Figure S6. *In situ* ^{1}H-^{1}H COSY NMR spectra of the reaction with **4** and [2,4,6-tri-*tert*-butylpyridinium][BACl] with catalytic [**1**][BArCl] in anhydrous d_2-DCM.

4.4 Reaction between 4 and [TBP-H][BArCl]

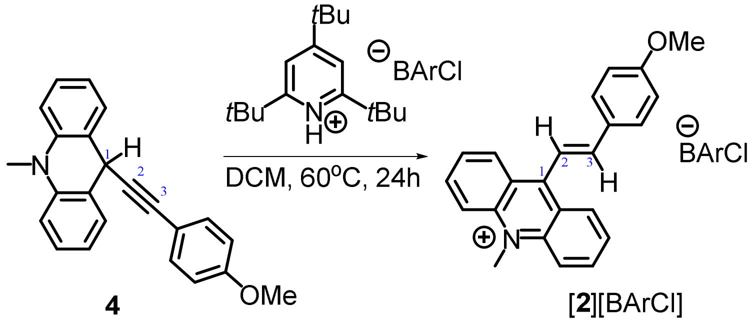

Under inert atmosphere, a J Youngs NMR tube was charged with **4** (4 mg, 0.012 mmol, 1.0 eq.) and 2,4,6-tri-*tert*-butylpyridinium tetra(3,5-dichlorophenyl)borate (10 mg, 0.012 mmol, 1.0 eq.), followed by the addition of d_2-DCM (0.5 mL). After initial monitoring of the reaction by multinuclear NMR spectroscopy the system was heated at 60°C for 24 hours. Upon heating, the ^{1}H NMR spectrum revealed the disappearance of the starting material **4**, with concomitant formation of [**2**]$^{+}$ and 2,4,6-tri-*tert*-butylpyridine. MS: m/z calcd for $C_{23}H_{20}NO^{+}$ [**2**]$^{+}$ 326.1 Found ES^{+} 326.2. m/z calcd for $C_{24}H_{12}BCl_8^{+}$ [BArCl] 594.8 Found ES^{+} 594.9.

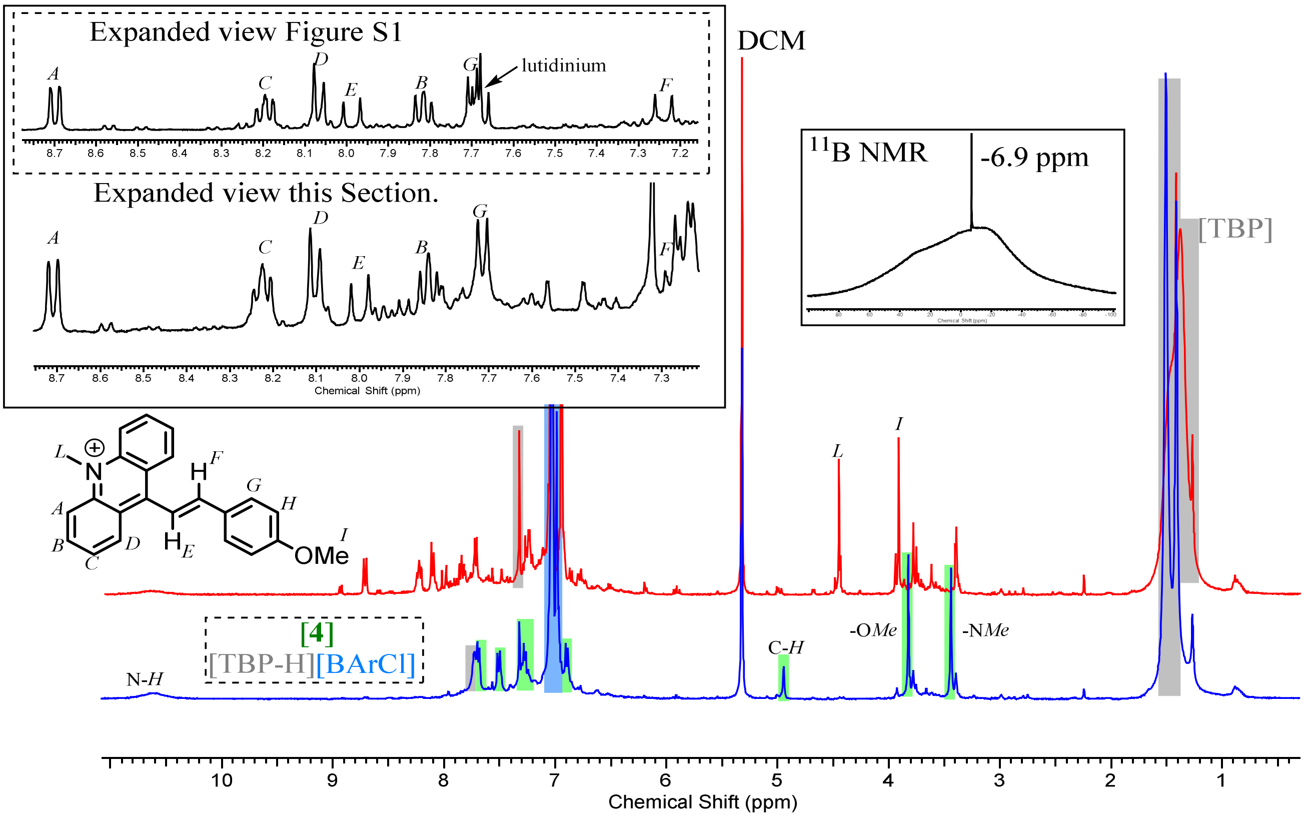

Figure S7. *In situ* ^{1}H-NMR spectra of the reaction with **4** and [2,4,6-tri-*tert*-butylpyridinium][BACl] in anhydrous d_2-DCM. Blue (t = 5 min, r.t.), red (after 24 h at 60°C). Inset, ^{11}B-NMR spectrum after 24 h at 60°C. The expanded views show the comparison between the reaction after 24 h at 60°C (bottom) and the reaction reported in Section 2 (top).

4.5 Reaction between 4 and [TBP-H][AlCl$_4$]

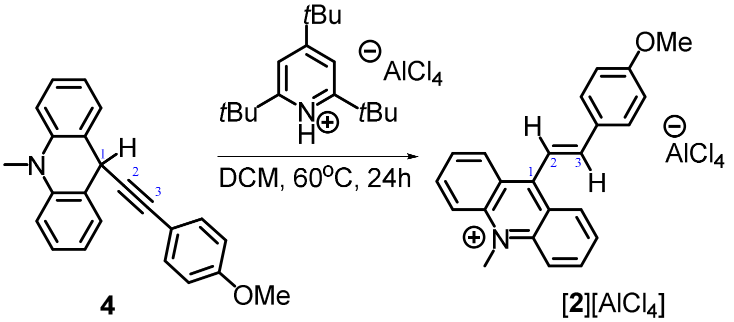

4 [**2**][AlCl$_4$]

Under inert atmosphere, a J Youngs NMR tube was charged with **4** (23 mg, 0.012 mmol, 1.0 eq.) and 2,4,6-tri-*tert*-butylpyridinium tetrachloroaluminate (38 mg, 0.012 mmol, 1.0 eq.), followed by the addition of *d*$_2$-DCM (0.5 mL). After initial monitoring of the reaction by multinuclear NMR spectroscopy the system was heated at 60°C for 24 hours. Upon heating, the ^{1}H NMR spectrum revealed the disappearing of the starting material **4**, with concomitant formation of [**2**]$^+$ and 2,4,6-tri-*tert*-butylpyridine. MS: m/z calcd for $C_{23}H_{20}NO^+$ [**2**]$^+$ 326.1 Found ES$^+$ 326.8. Due to different degree of cation-anion interaction (BArCl or $AlCl_4^-$), the protons on the acridinium unit (including the methyl group) are slightly shifted in [**2**]$^+$.

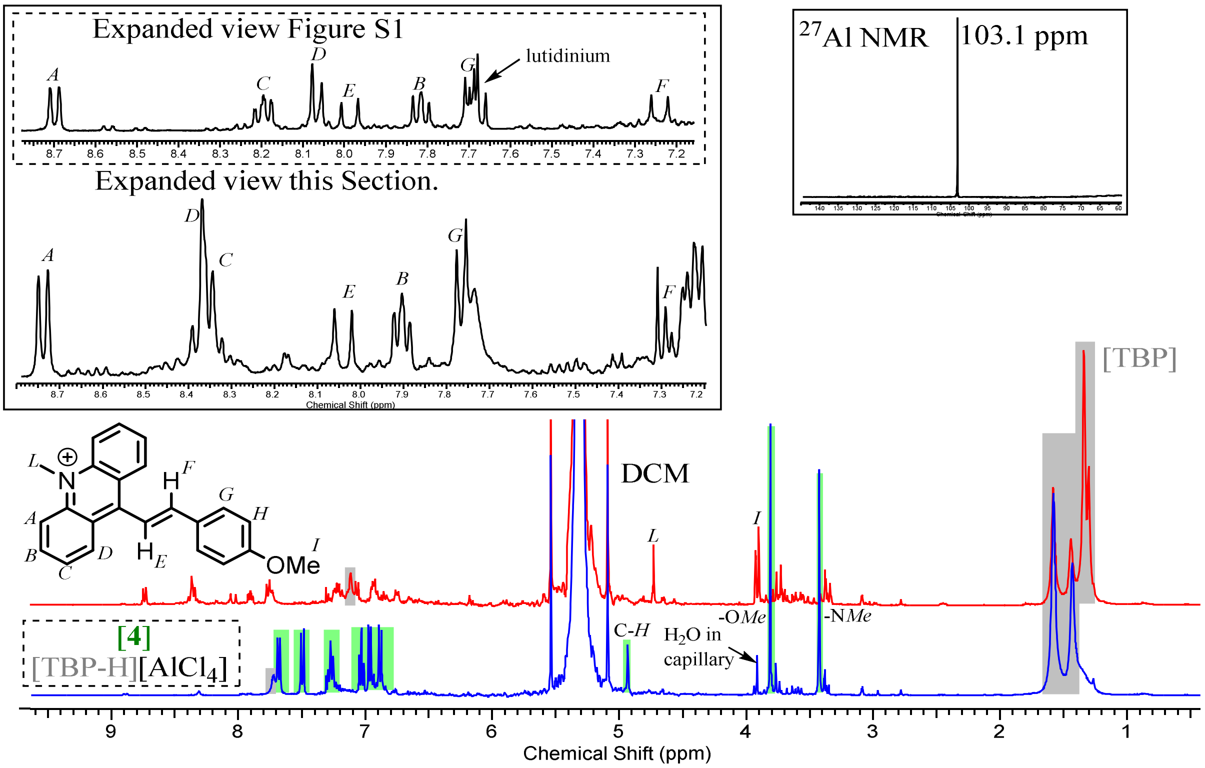

Figure S8. *In situ* ^{1}H-NMR spectra of the reaction with **4** and [2,4,6-tri-*tert*-butylpyridinium][AlCl$_4$] in anhydrous *d*$_2$-DCM. Blue (t = 5 min, r.t.), red (after 24 h at 60°C). Inset, ^{27}Al-NMR spectrum after 24 h at 60°C. The expanded views show the comparison between the reaction after 24 h at 60°C (bottom) and the reaction reported in Section 2 (top).

4.6 Reaction between 4D and [2,6-lutidinium][$AlCl_4$]

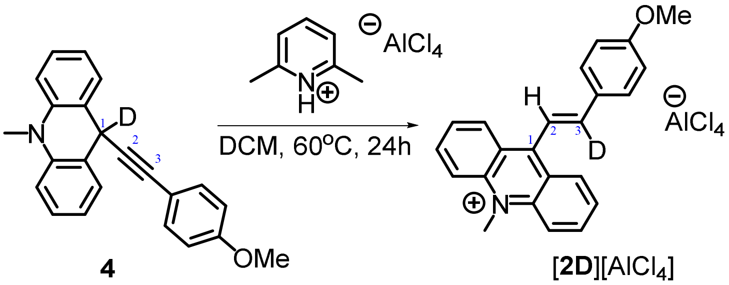

Under inert atmosphere, a J Youngs NMR tube was charged with **4D** (16 mg, 0.049 mmol, 1.0 eq.) and 2,6-lutidinium tetrachloroaluminate (14 mg, 0.049 mmol, 1.0 eq.), followed by the addition of d_2-DCM (0.5 mL). After initial monitoring of the reaction by multinuclear NMR spectroscopy the system was heated at 60°C for 24 hours. Upon heating, the ^{1}H NMR spectrum revealed the disappearing of the starting material **4D**, with concomitant formation of 2,4,6-tri-*tert*-butylpyridine and [**2D**]$^+$ (diagnostic due to the signal E that collapses from a doublet in [**2**]$^+$ to a singlet in [**2D**]$^+$). MS: m/z calcd for $C_{23}H_{19}DNO^+$ [**2D**]$^+$ 327.1 Found ES$^+$ 327.2. Due to different degree of cation-anion interaction (BArCl or $AlCl_4^-$), the protons on the acridinium unit (including the methyl group) and those of the lutidinium are slightly shifted.

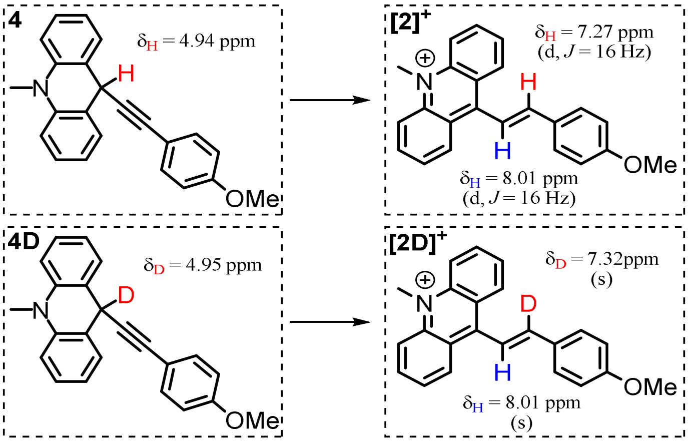

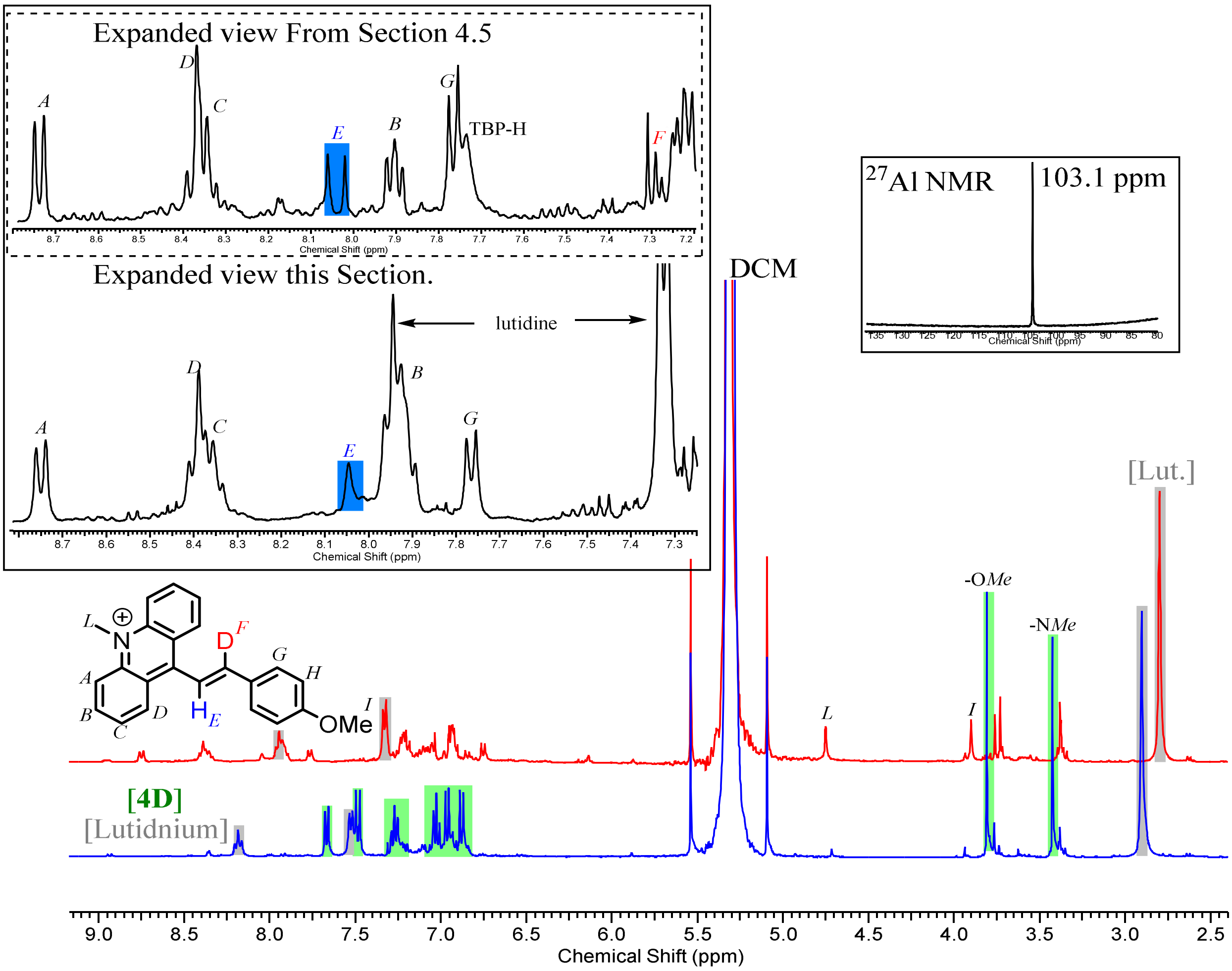

Figure S9. *In situ* ^{1}H-NMR spectra of the reaction with **4D** and [2,6-lutidinium][$AlCl_4$] in anhydrous d_2-DCM. Blue (t = 5 min, r.t.), red (after 24 h at 60°C). Inset, ^{27}Al-NMR spectrum after 24 h at 60°C. The expanded views show the comparison between the reaction after 24 h at 60°C (bottom) and the reaction reported in Section 4.5 (top).

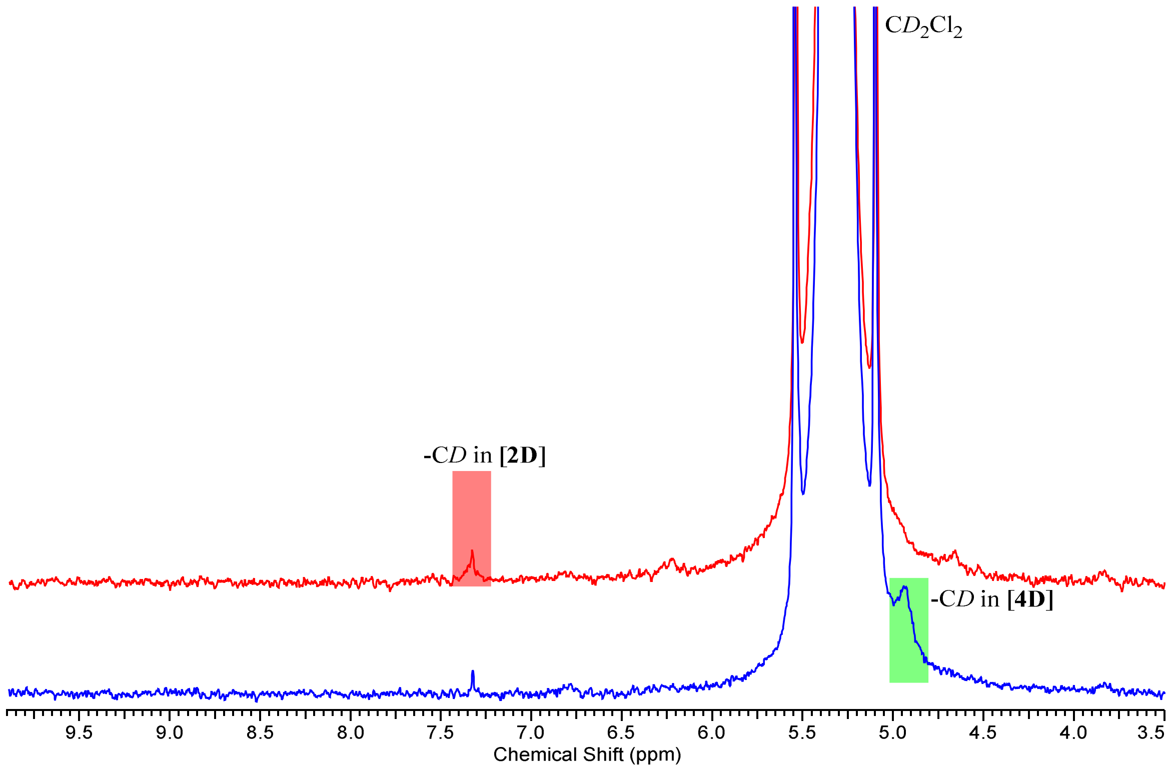

Figure S10. *In situ* ^{2}D-NMR spectra of the reaction with **4D** and [2,6-lutidinium][$AlCl_4$] in anhydrous d_2-DCM. Blue (t = 5 min, r.t.), red (after 24 h at 60°C).

5. [7][I] synthesis

5.1 Synthesis of 1-H

- Synthesis of *N*-methyl-acridinium iodide

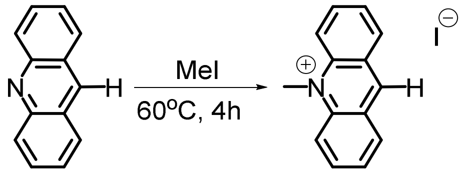

Acridine (1.4 g, 7.800 mmol, 1.0 eq.) was dissolved in an excess of iodomethane (6 mL, > 10.0 eq.), within a sealed ampule fitted with a J. Young's stopcock. The reaction vessel was heated at 60°C for 4 hours and then iodomethane was removed under vacuum, leaving a red solid. The latter was washed with hexane affording *N*-methyl-acridinium iodide as a red solid (1.4 g, 4.360 mmol, 56%). ^{1}H NMR (400 MHz, d_6-DMSO) δ 10.21 (s, 1H), 8.79 (d, J = 9.0 Hz, 2H), 8.64 (d, J = 8.0 Hz, 2H), 8.47 (m, 2H), 8.04 (t, J = 7.0 Hz, 2H), 4.86 (s, 3H) ppm. ^{13}C{^{1}H} NMR (100 MHz, d_6-DMSO): δ 150.6, 141.3, 139.0, 131.6, 127.7, 126.2, 118.9, 38.6 ppm. The data are in accordance with those reported in the literature.[1]

- Synthesis of *N*-methyl-acridane 1-H

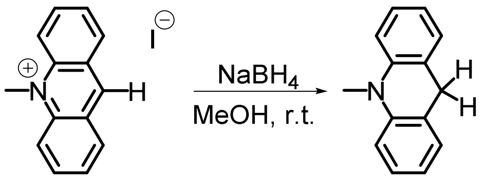

1-H

N-methyl-acridinium iodide (700 mg, 2.180 mmol, 1.0 eq.) was dissolved in MeOH (10 mL), obtaining a dark red solution. While stirring, $NaBH_4$ (247 mg, 6.540 mmol, 3.0 eq.) was added at room temperature. After a minute, the solution became pale yellow with a whitish precipitate. To this water was added in excess (10 mL). The solid was separated from the solution by filtration and left drying under vacuum at 40°C overnight, obtaining *N*-methyl-acridane **1**-H was obtained as a grey solid (158 mg, 0.809 mmol, 37%). ^{1}H NMR (400 MHz, $CDCl_3$) δ 7.17-7.42 (m, 4H), 7.16-6.87 (m, 4H), 4.03 (s, 2H), 3.48 (s, 3H) ppm. ^{13}C{^{1}H} NMR (100 MHz, $CDCl_3$): δ 143.6, 127.5, 126.8, 124.2, 120.5, 111.8, 33.2, 33.0 ppm. The data are in accordance with those reported in the literature.[1]

5.2 Reduction of [5][I] with 1-H to form [7][I]

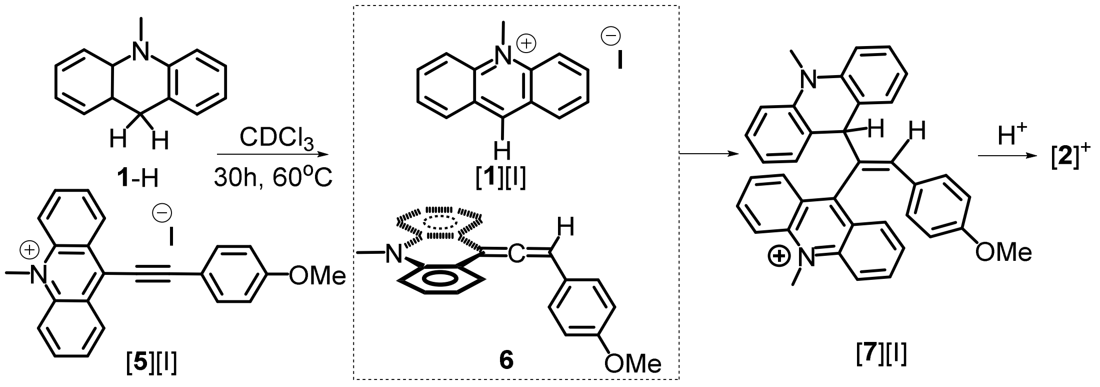

Under inert atmosphere, a J. Youngs NMR tube was charged with *N*-methyl-9-((4-methoxyphenyl)ethynyl)acridinium iodide (25 mg, 0.055 mmol, 1.00 eq.) and *N*-methyl-acridane (11 mg, 0.055 mmol, 1.00 eq.), followed by the addition of anhydrous *d*-$CDCl_3$ (0.8 mL). The system was then sealed and heated at 60°C for 30 h. In situ ^{1}H NMR spectroscopic analysis revealed the formation of a new major species whose full NMR characterization revealed to be the product [**7**][I]. ^{1}H NMR (400 MHz, $CDCl_3$) δ 8.70 (d, *J* = 9.0 Hz, 2H), 8.12 (t, *J* = 7.8 Hz, 2H), 7.49 (d, *J* = 8.4 Hz, 2H), 7.37 (s, 1H), 7.36 (d, *J* = 6.9 Hz, 2H), 7.19 (t, *J* = 7.9 Hz, 2H), 6.94-6.97 (m, 2H), 6.92 (t, *J* = 7.9 Hz, 2H), 6.68 (d, *J* = 8.7 Hz, 2H), 6.50 (t, *J* = 8.7 Hz, 2H), 6.09 (d, *J* = 7.3 Hz, 2H), 5.57 (s, 1H), 5.10 (s, 3H), 3.58 (s. 3H), 2.17 (s, 3H) ppm. $^{13}C\{^1H\}$ NMR (100 MHz, $CDCl_3$) δ 159.5, 159.1, 141.4, 139.7, 138.7, 134.4, 130.3, 130.2, 129.1, 128.8, 128.4, 127.9, 126.0, 125.5, 122.3, 121.6, 118.2, 114.4, 112.2, 56.8, 55.1, 41.1, 31.8 ppm. ESI$^+$ of $[C_{37}H_{31}N_2O]^+$ [**7**]$^+$ 519.1; found 519.1. Accurate mass: $[C_{37}H_{31}N_2O]^+$ [**7**]$^+$ 519.2431; found: 519.2428.

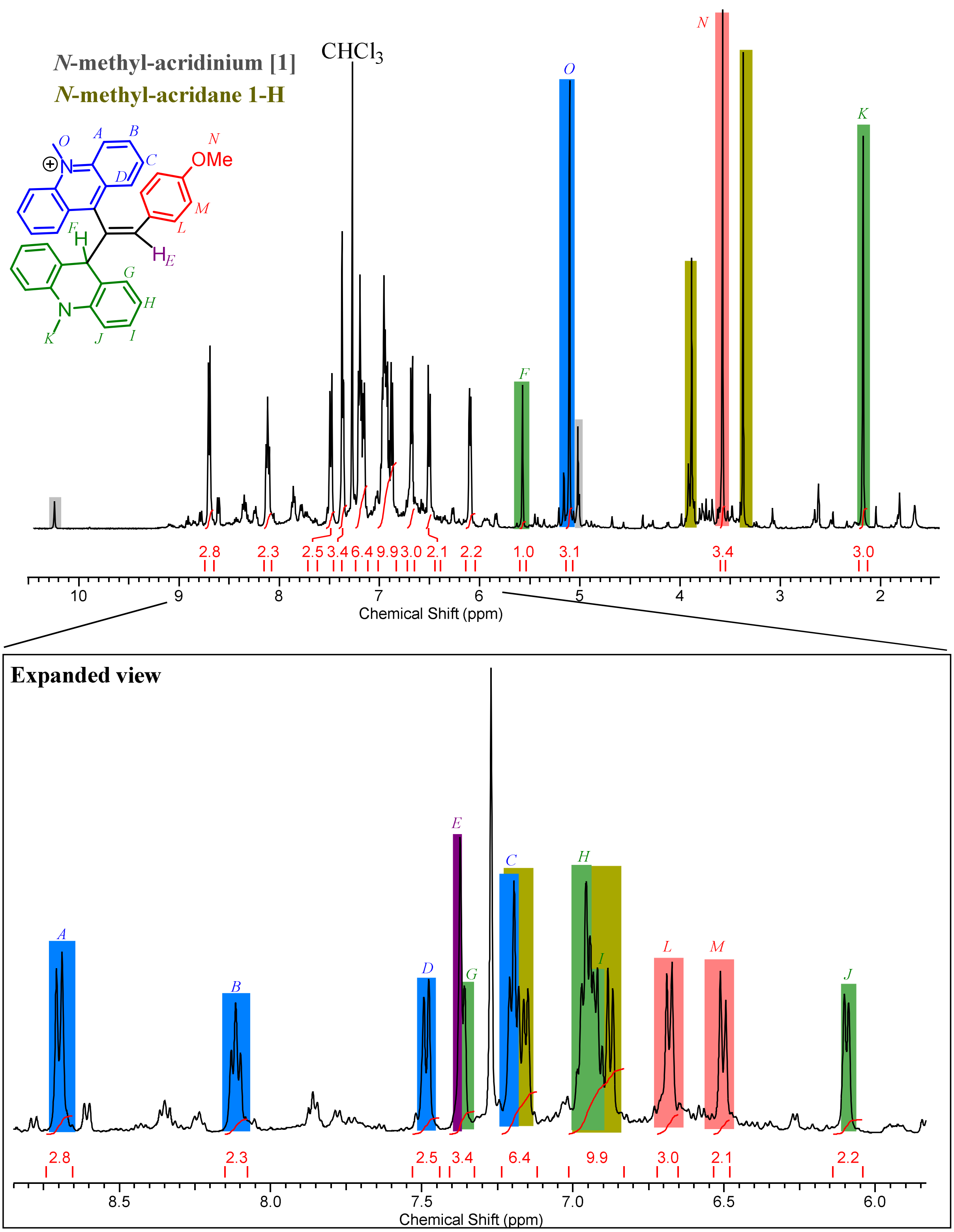

Figure S11. In situ ^{1}H NMR spectrum of the reaction between [**5**][I] and **1**-H in $CDCl_3$ after heated at 60°C for 30 h.

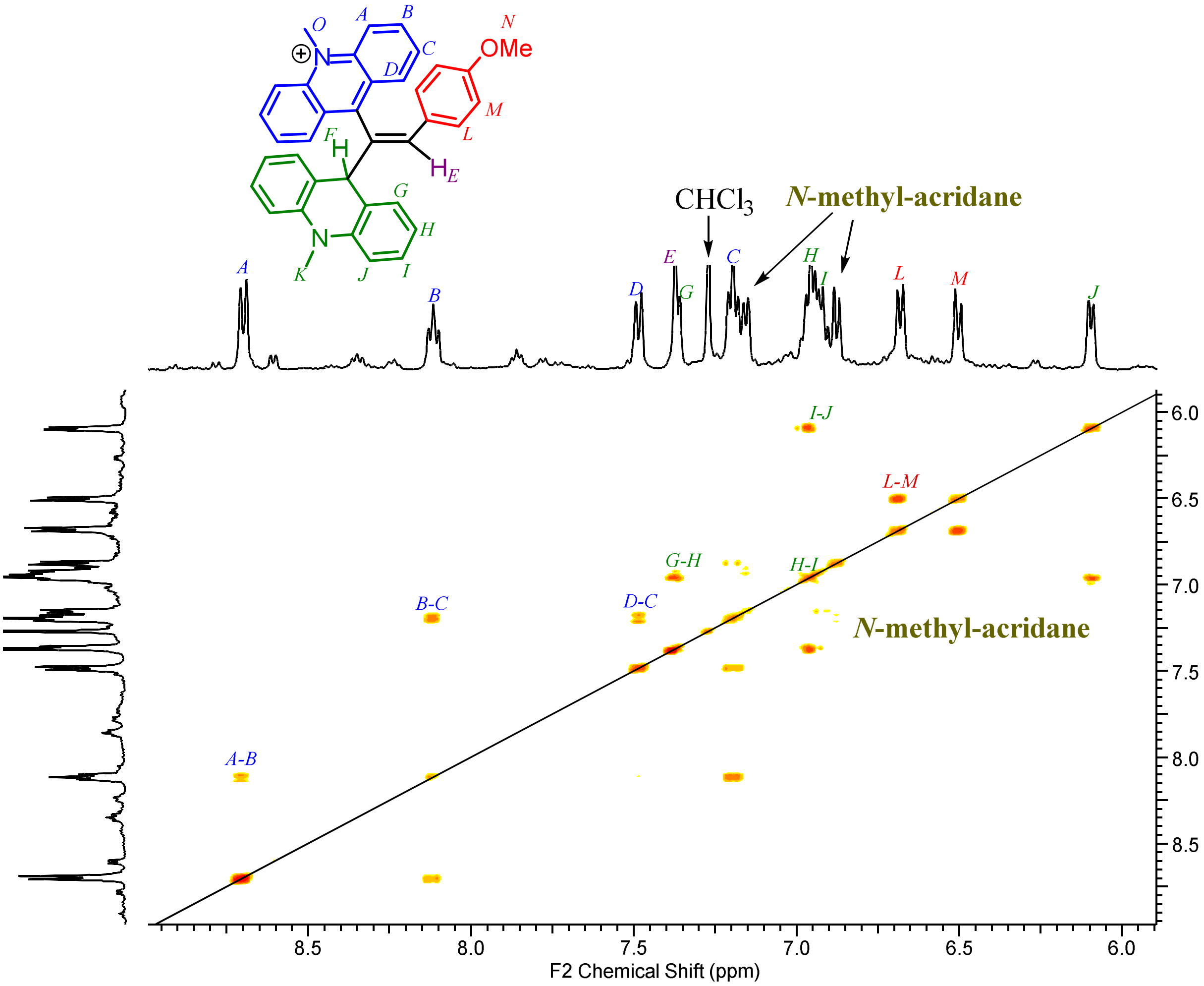

Figure S12. In situ ^{1}H-^{1}H COSY NMR spectrum of the reaction between [**5**][I] and **1**-H in $CDCl_3$ after heated at 60°C for 30h.

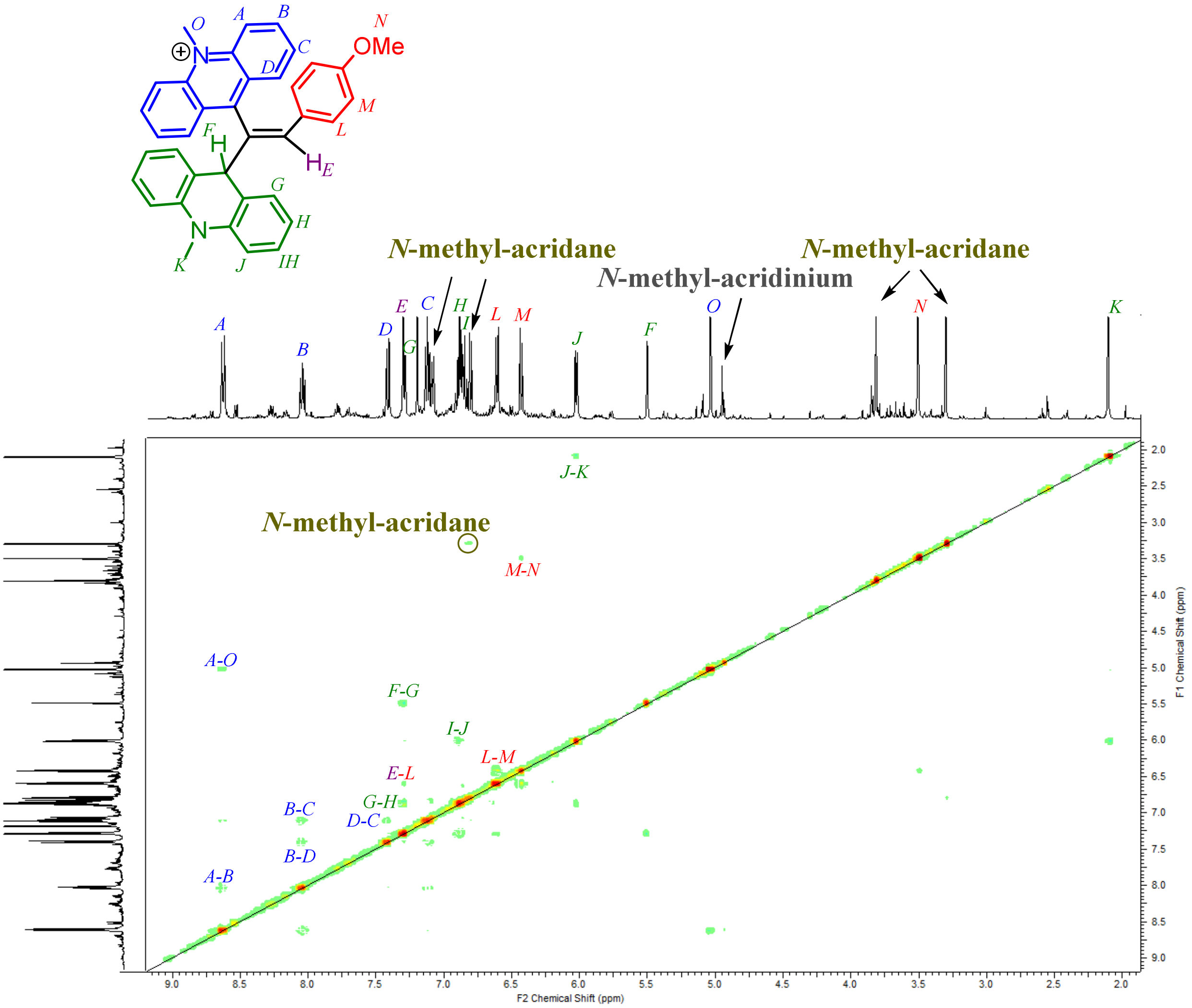

Figure S13. In situ ^{1}H-^{1}H NOESY NMR spectrum of the reaction between [**5**][I] and **1**-H in *d*-$CDCl_3$ after heated at 60°C for 30h. Due to proximity of the peaks E and G, any correlation involving D, F and L to assign the *E/Z* isomerism was inconclusive (the stereochemistry was unambiguously clarified by X-ray diffraction analysis).

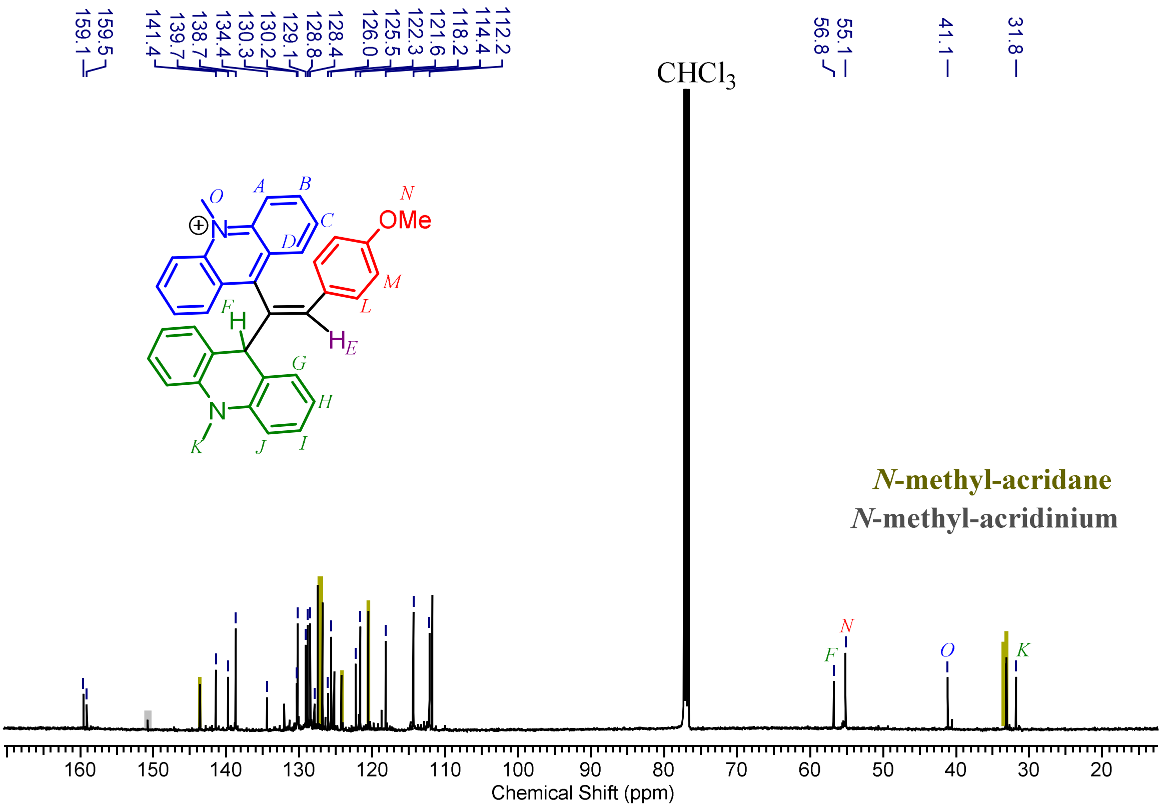

Figure S14. In situ $^{13}C\{^1H\}$ NMR spectrum of the reaction between [**5**][I] and **1**-H in $CDCl_3$ after heated at 60°C for 30 h.

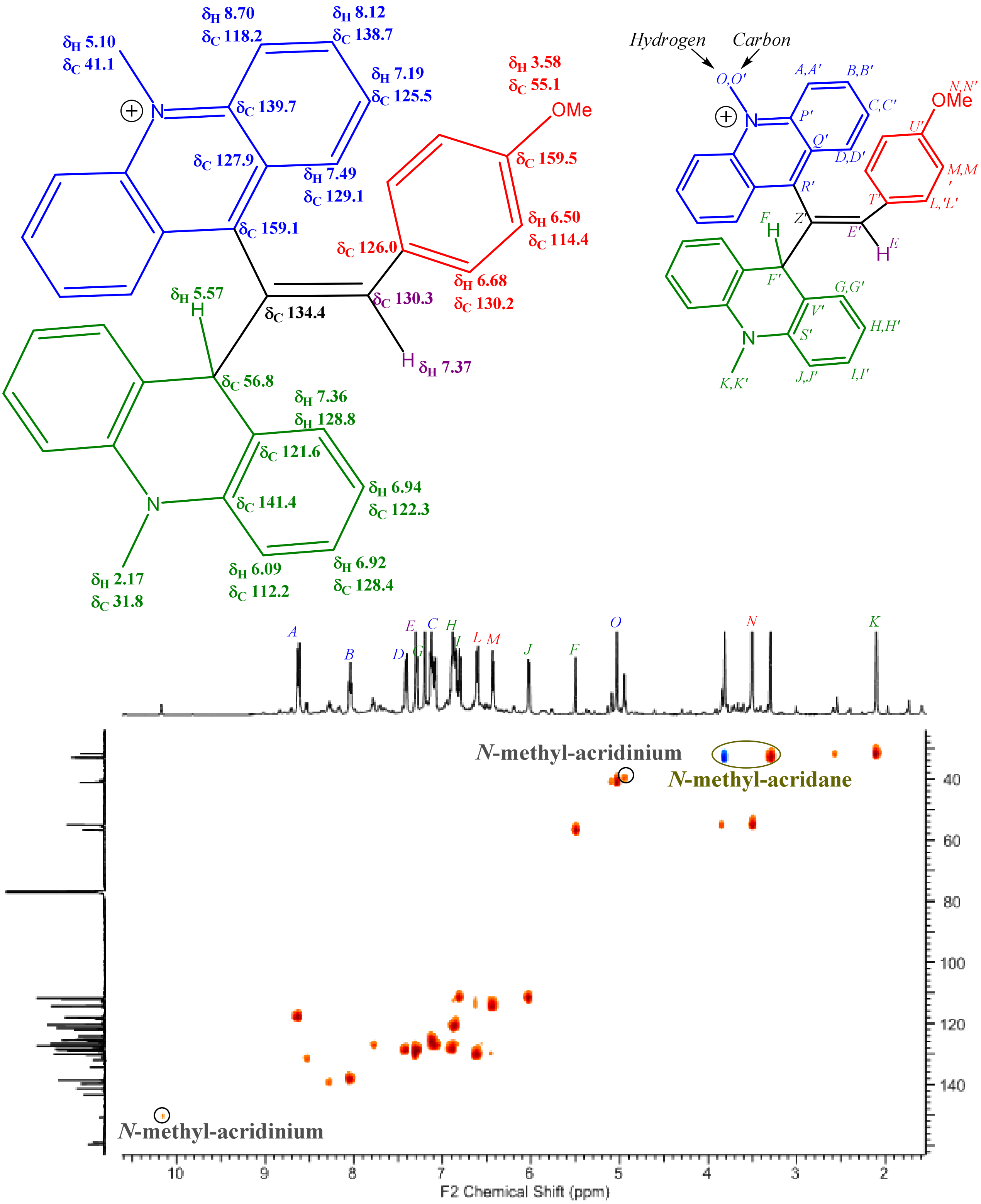

Figure S15. In situ ^{13}C-^{1}H HSQC NMR spectrum of the reaction between [**5**][I] and **1**-H in $CDCl_3$ after heated at 60°C for 30h.

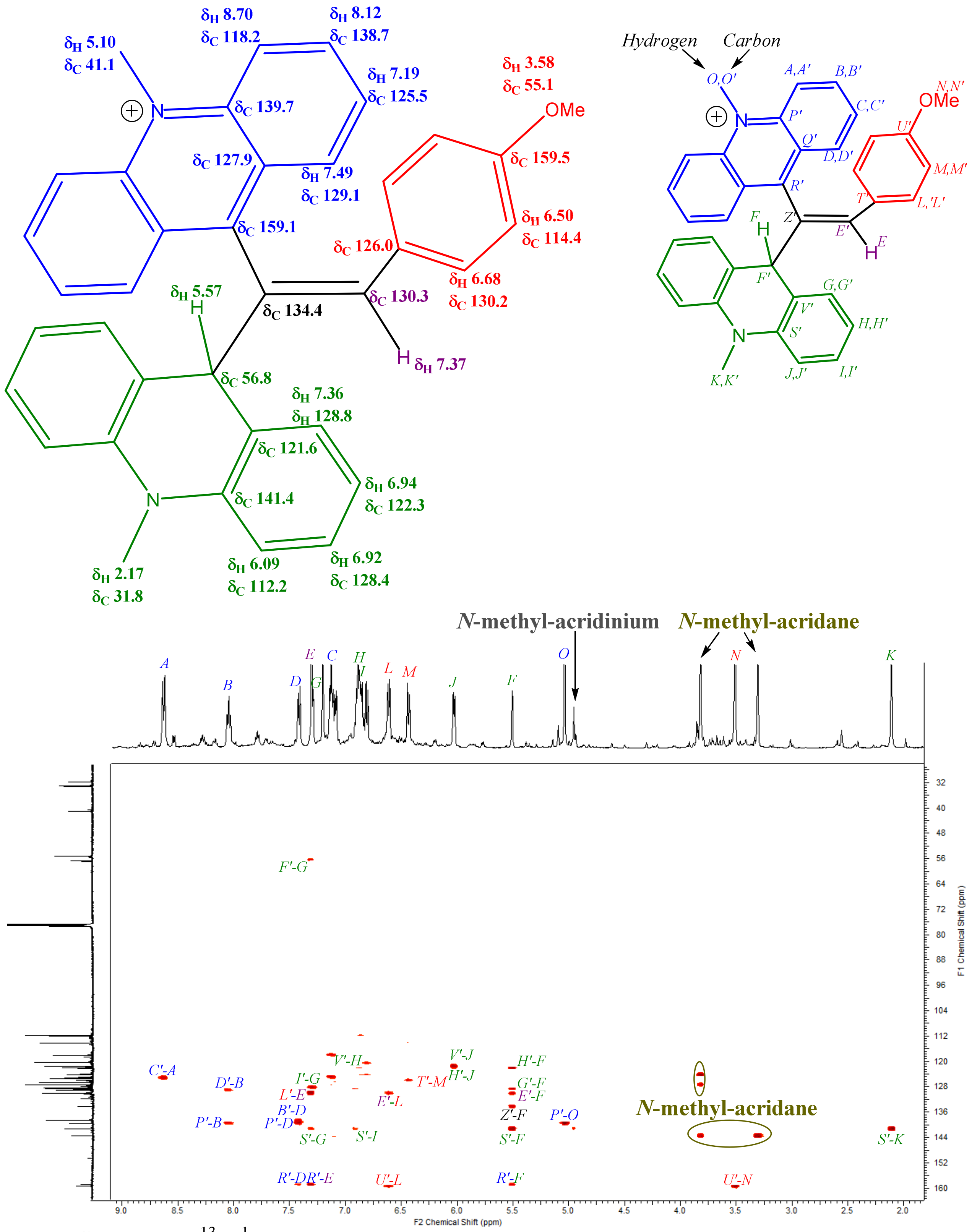

Figure S16. In situ ^{13}C-^{1}H HMBC NMR spectrum of the reaction between [**5**][I] and **1**-H in *d*-$CDCl_3$ after heated at 60°C for 30h.

Single crystals of [**7**][I] were isolated from the chloroform solution layered with hexane.

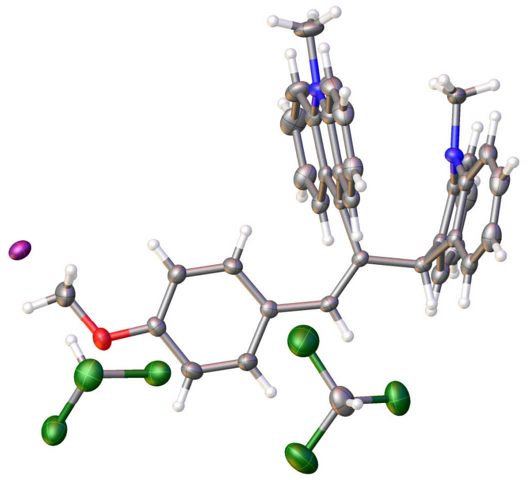

The reaction system was then concentrated under reduced pressure and washed with hexane (causing a precipitate to form) to remove the unreacted **1**-H. Then, [**7**][I] was redissolved in $CDCl_3$ (0.5 mL) in a J. Youngs NMR tube, followed by the addition of 2,6-lutidinium tetrachloroaluminate (15 mg, 0.055 mmol, 1.0 eq.). The tube was then sealed and heated at 60°C for 2 hours. ^{1}H NMR spectroscopy revealed that upon heating all [**7**]$^+$ had been consumed forming equimolar amount of [**2**]$^+$ (pair of doublet with *J* = 16Hz) and [**1**]$^+$ (proton on C9 at 10 ppm).

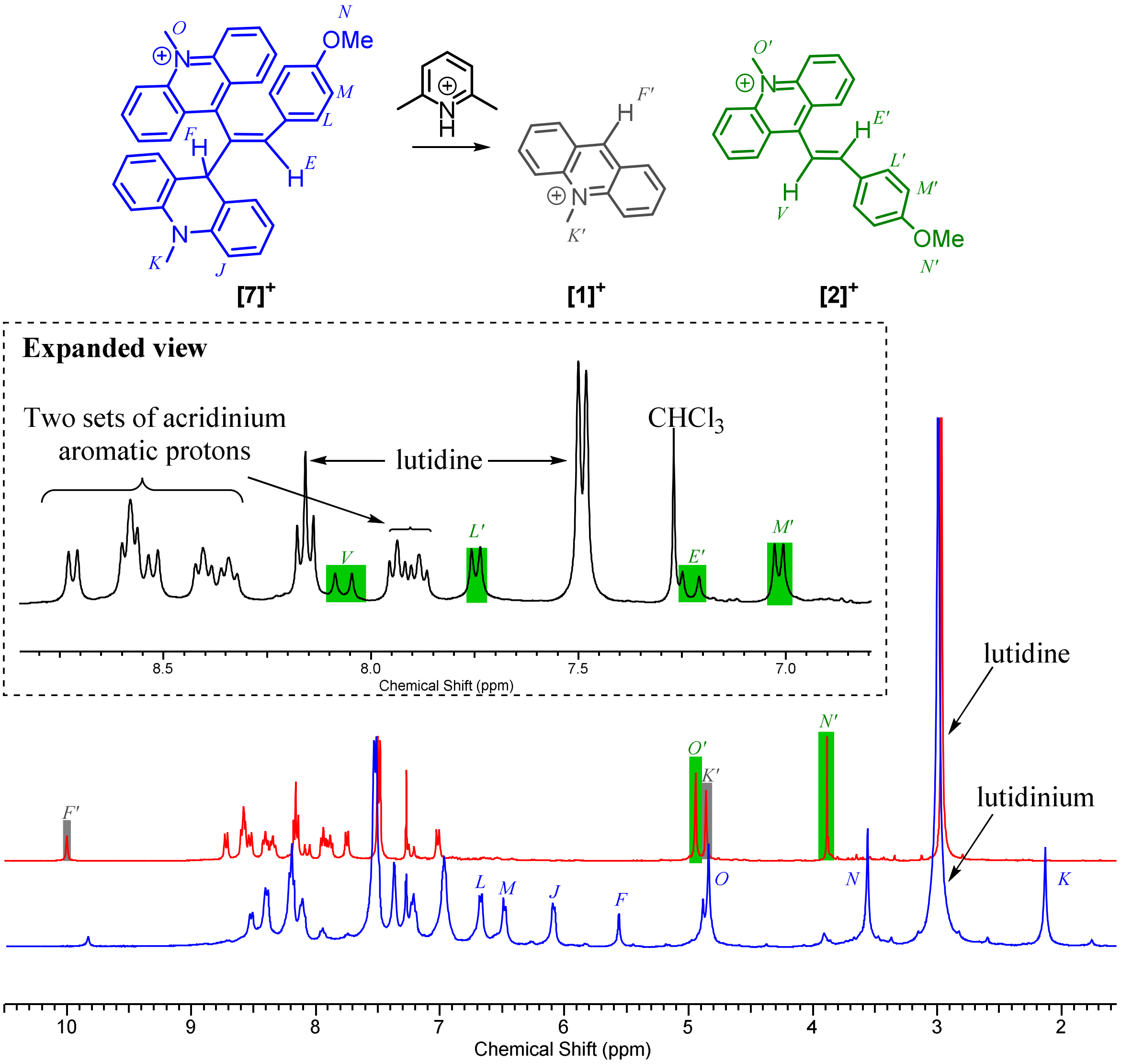

Figure S17. In situ ^{1}H NMR spectra of the reaction between [**7**][I] and [2,6-lutidinium][$AlCl_4$] in $CDCl_3$. Blue (on mixing), red (after heated at 60°C for 2h).

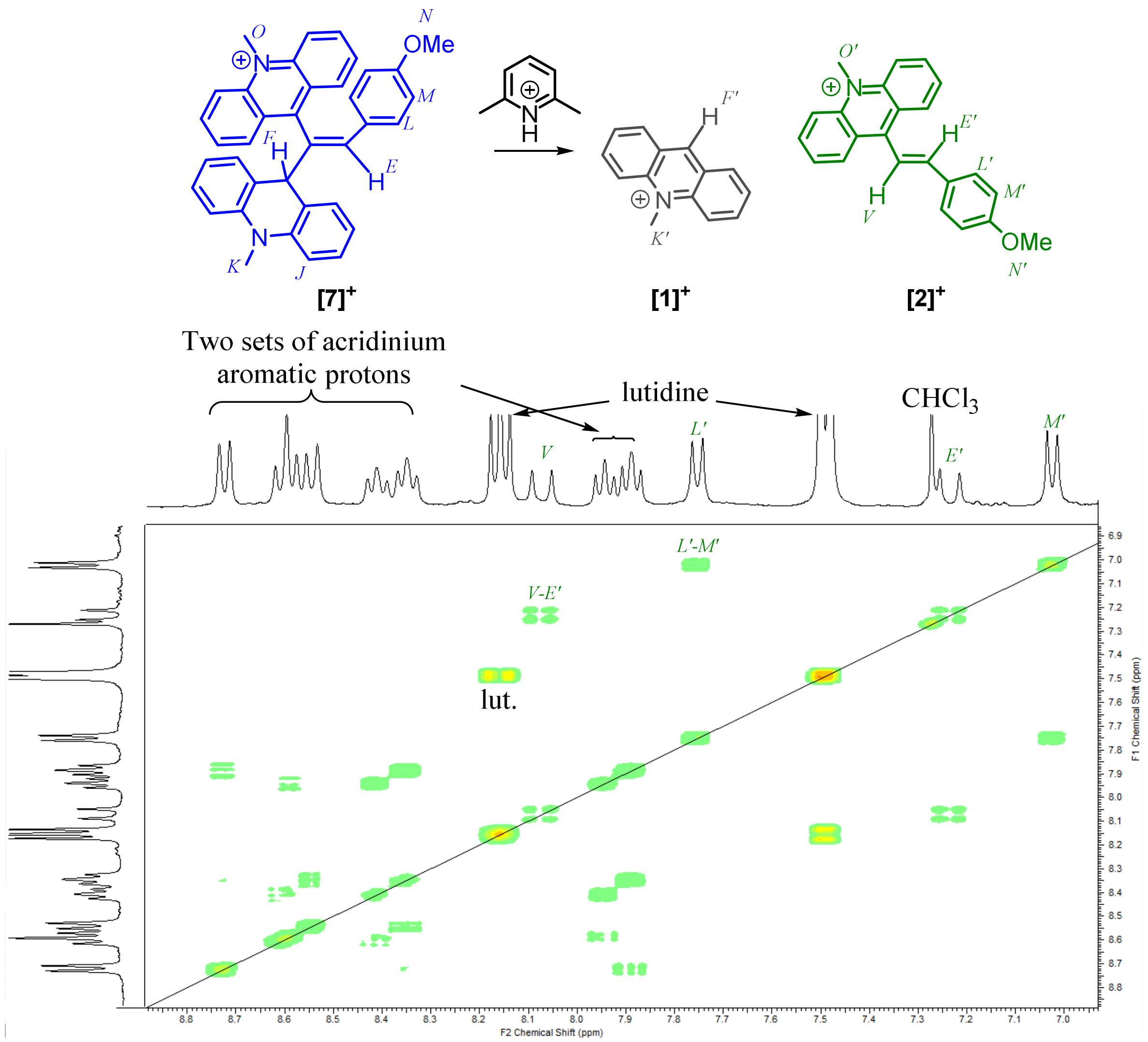

Figure S18. In situ ^{1}H-^{1}H COSY NMR spectrum of the reaction between [**7**][I] and [2,6-lutidinium][$AlCl_4$] in $CDCl_3$, after heated at 60°C for 2h.

6. Synthesis and reactivity of the intermediate 8 with [2,6-lutidinium][$AlCl_4$]

6.1 Synthesis of intermediate 8

- Synthesis of 9-(p-tolylethynyl)acridine

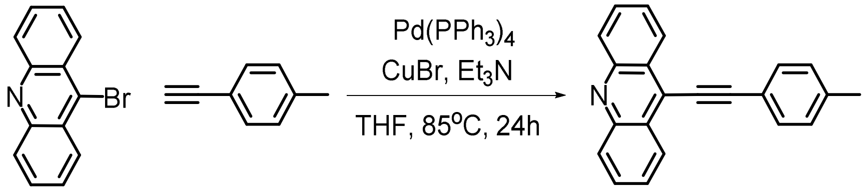

A Schlenk tube was charged with tetrakis-(triphenylphosphine)palladium(0) (88 mg, 0.075 mmol, 0.02 eq.) and copper(I) bromide (22 mg, 0.150 mmol, 0.04 eq.). Tetrahydrofuran was added and the suspension was stirred for five minutes, followed by the addition of the 9-bromoacridine (1g, 3.750 mmol, 1 eq.) and triethylamine (6 mL). After five minutes under stirring, 4-ethynyltoluene (490 µL, 4.125 mmol, 1.1 eq.) was added and the solution was stirred and refluxed at 85°C for 24 h. The solution was cooled to room temperature, filtered through celite/silica and eluted with diethyl ether (3 x 10 mL). The solvent was removed in vacuo and the crude material was purified via column chromatography (petr. ether: AcOEt 80:20) yielding the corresponding product as a yellow solid (742 mg, 2.529 mmol, 67%). R_f = 0.3. ^{1}H NMR (400 MHz, $CDCl_3$) δ 8.58 (d, *J* = 9.3 Hz, 2H), 8.25 (d, *J* = 8.8 Hz, 2H), 7.82 (t, *J* = 8.0 Hz, 2H), 7.70 (d, *J* = 8.0 Hz, 2H), 7.64 (t, *J* = 8.5 Hz, 2H), 7.29 (d, *J* = 7.8 Hz, 2H), 2.45 (s, 3H) ppm. ^{13}C{^{1}H} NMR (100 MHz, $CDCl_3$): δ 148.6, 140.0, 131.9, 130.3, 129.9, 129.4, 128.1, 126.7, 126.5, 126.4, 119.4, 105.6, 83.6, 21.7 ppm. GC-MS: m/z calculated for $C_{22}H_{15}N$, 293.4; found 293.1 (retention time of analyte: 15.83 minutes). Accurate mass: 293.1277; found: 293.1274.

- Synthesis of *N*-methyl-9-(p-tolylethynyl)acridium iodide

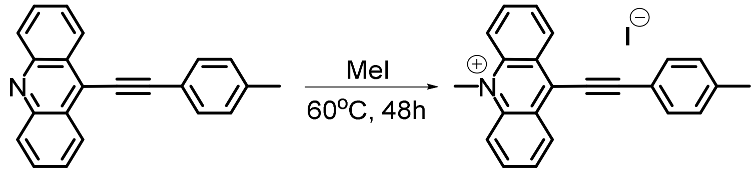

9-(p-tolylethynyl)acridine (560 mg, 1.852 mmol, 1.0 eq.) was dissolved in an excess of iodomethane (2.0 mL, > 10.0 eq.), within a sealed ampule fitted with a J. Young's stopcock. The reaction vessel was heated at 60°C for 2 days and then iodomethane was removed under vacuum, leaving a dark red solid. The latter was washed with hexane affording *N*-methyl-9-(p-tolylethynyl)acridinium iodide as a red-brown solid (585 mg, 1.344 mmol, 72%). ^{1}H NMR (400 MHz, *d*$_6$-DMSO) δ 9.04 (d, *J* = 7.9 Hz, 2H), 8.80 (d, *J* = 9.2 Hz, 2H), 8.47 (t, *J* = 8.7 Hz, 2H), 8.13-8.06 (m, 4H), 7.48 (d, *J* = 8.1 Hz, 2H), 4.83 (s, 3H), 2.47 (s, 3H) ppm. ^{13}C{^{1}H}

NMR (100 MHz, d_6-DMSO): δ 143.1, 141.0, 140.4, 138.5, 133.6, 130.0, 129.3, 128.7, 126.2, 119.5, 116.9, 116.4, 84.0, 38.8, 21.5 ppm. MS: m/z calcd for $C_{23}H_{18}N^+$ 308.4 Found ES^+ 308.2, m/z calcd for I^- 126.9 Found ES^- 126.8. Accurate mass for $C_{23}H_{18}N^+$ 308.1434 Found 308.1426.

- Synthesis of *N*-methyl-9-(p-tolylethynyl)acridane 8

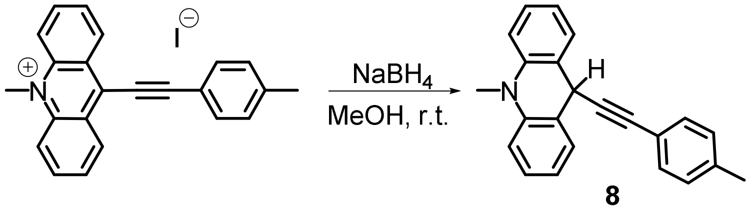

8

N-methyl-9-(p-tolylethynyl)acridinium iodide (25 mg, 0.058 mmol, 1.0 eq.) was dissolved in MeOH (2 mL), obtaining a dark red solution. While stirring, $NaBH_4$ (4 mg, 0.084 mmol, 1.5 eq.) was added at room temperature. After a few seconds, the solution became green, and an excess of water was added to quench the reaction. The solution was extracted two times with Et_2O (3 mL) and the organic phase was dried on anhydrous $MgSO_4$. After filtration, the residue was purified on a plug of silica, obtaining the desired compound **8** as a yellow oil (15 mg, 0.048 mmol, 84%). ^1H NMR (400 MHz, $CDCl_3$) δ 7.71 (d, *J* = 7.5 Hz, 2H), 7.48 (d, *J* = 8.0 Hz, 2H), 7.30 (t, *J* = 8.3 Hz, 2H), 7.19 (d, *J* = 8.3 Hz, 2H), 7.06 (t, *J* = 7.5 Hz, 2H), 6.99 (d, *J* = 8.3 Hz, 2H), 4.97 (s, 1H), 3.44 (s, 3H), 2.38 (s, 3H) ppm. $^{13}C\{^1H\}$ NMR (100 MHz, $CDCl_3$): δ 143.5, 139.1, 132.1, 129.7, 128.1, 127.0, 124.6, 121.4, 120.8, 112.7, 87.4, 87.0, 35.7, 33.8, 21.8 ppm. *Note*: the compound was found instable on flash chromatography, so it was used as obtained after filtration on a plug of silica.

6.2 Reaction between 8 and [2,6-lutidinium][$AlCl_4$]

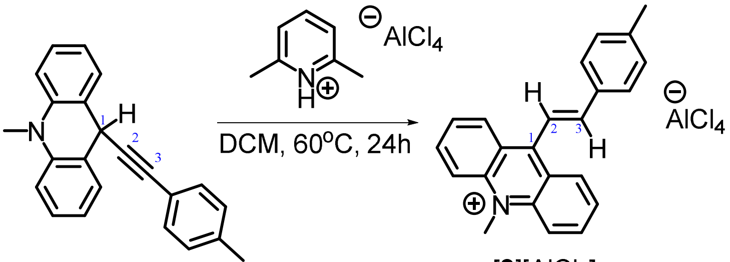

8 [**9**][$AlCl_4$]

Under inert atmosphere, a J Youngs NMR tube was charged with **8** (15 mg, 0.046 mmol, 1.0 eq.) and 2,6-lutidinium tetrachloroaluminate (13 mg, 0.046 mmol, 1.0 eq.), followed by the addition of d_2-DCM (0.5 mL). After initial monitoring of the reaction by multinuclear NMR spectroscopy the system was heated at 60°C for 24 hours. Upon heating, the ^{1}H NMR spectrum revealed the disappearing of the starting material **8**, with concomitant formation of 2,4,6-tri-*tert*-butylpyridine and [**9**]$^+$. ^{1}H NMR (400 MHz, CD_2Cl_2) δ 8.73 (d, J = 8.5 Hz, 2H), 8.40 (t, J = 8.5 Hz, 2H), 8.36 (d, J = 8.3 Hz, 2H), 8.01 (d, J = 16.3 Hz, 1H), 7.92 (d, J = 7.9 Hz, 2H), 7.69 (d, J = 8.0 Hz, 2H), 7.36 (d, J = 8.0 Hz, 2H), 7.31 (m, 1H), 4.76 (s, 3H), 2.45 (s, 3H) ppm. ^{13}C NMR (100 MHz, CD_2Cl_2) δ 159.0, 147.4, 142.3, 141.8, 139.4, 133.0, 130.5, 129.7, 128.6, 128.2, 125.5, 119.5, 119.2, 40.3, 21.9 ppm. ^{27}Al NMR (104 MHz, CD_2Cl_2) δ 103.7 ppm. MS: m/z calcd for $C_{23}H_{20}N^+$ [**9**]$^+$ 310.1 Found ES$^+$ 310.8. Accurate mass for $C_{23}H_{20}N^+$ [**9**]$^+$ 310.1590 Found 310.1584.

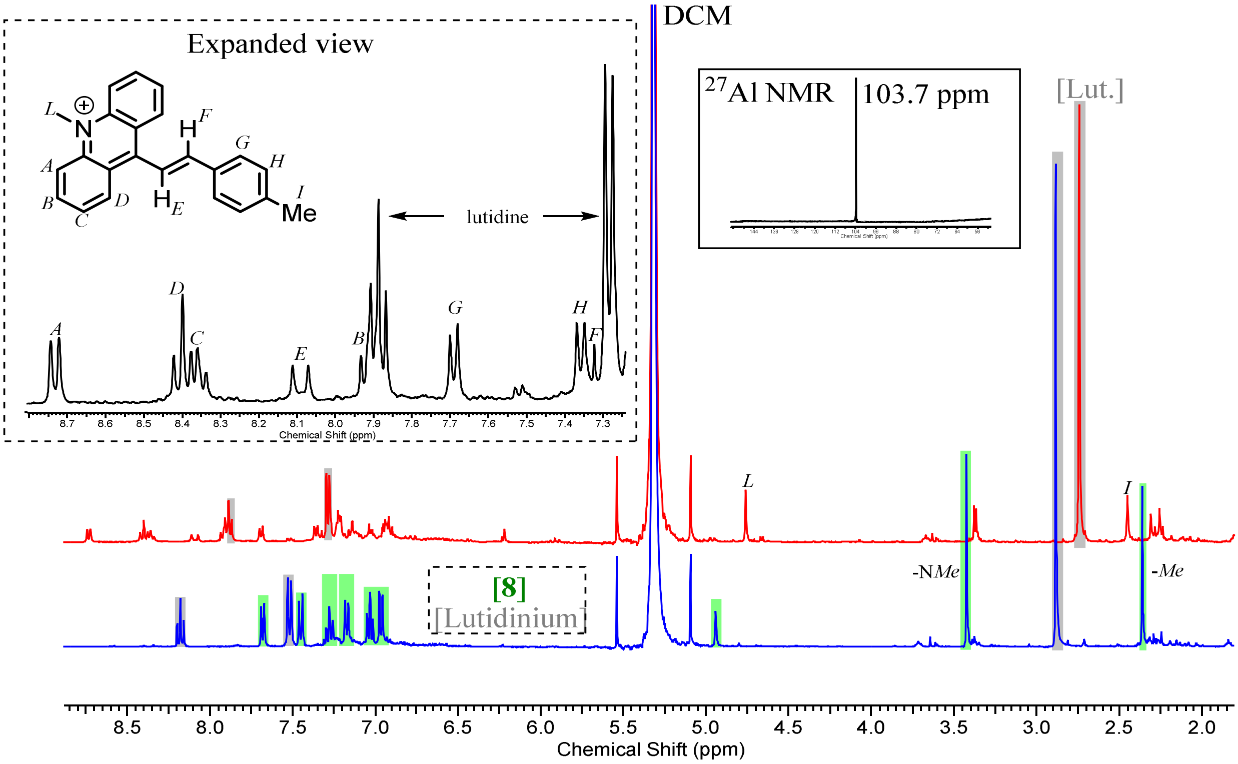

Figure S19. *In situ* ^{1}H-NMR spectra of the reaction with **8** and [2,6-lutidinium][$AlCl_4$] in anhydrous d_2-DCM. Blue (t = 5 min, r.t.), red (after 24 h at 60°C). Inset, ^{27}Al-NMR spectrum after 24 h at 60°C.

The sample was then washed with hexane and characterized by 2D NMR (some lutidine was still left). The diagnostic scalar coupling (J = 16 Hz) and the 2D NMR analysis confirmed the E configuration of the product.

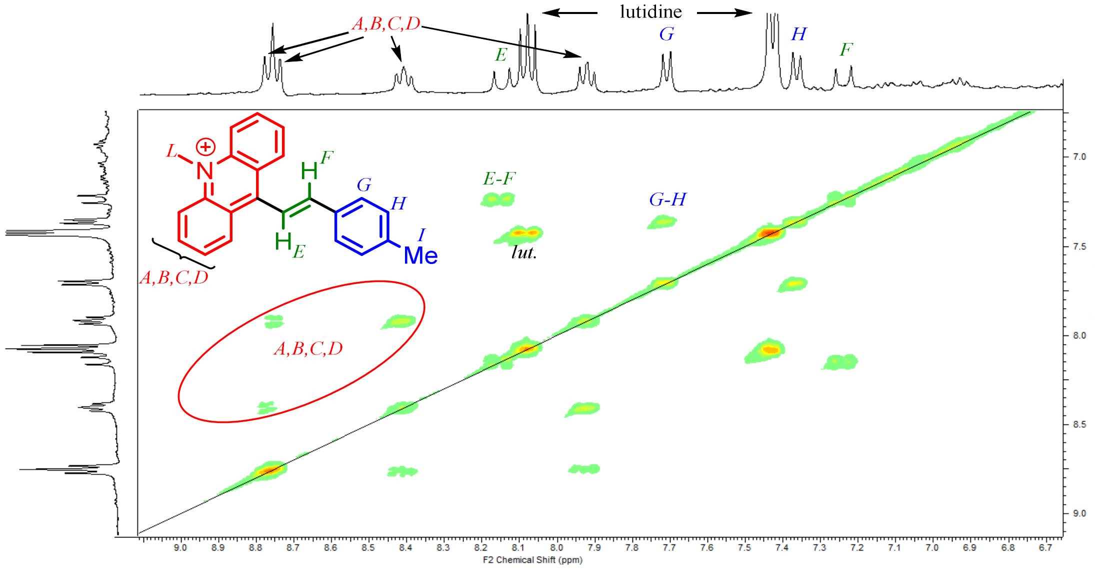

Figure S20. ^{1}H-^{1}H COSY NMR spectra of the reaction with **8** and [2,6-lutidinium][$AlCl_4$] in d_2-DCM after hexane washing.

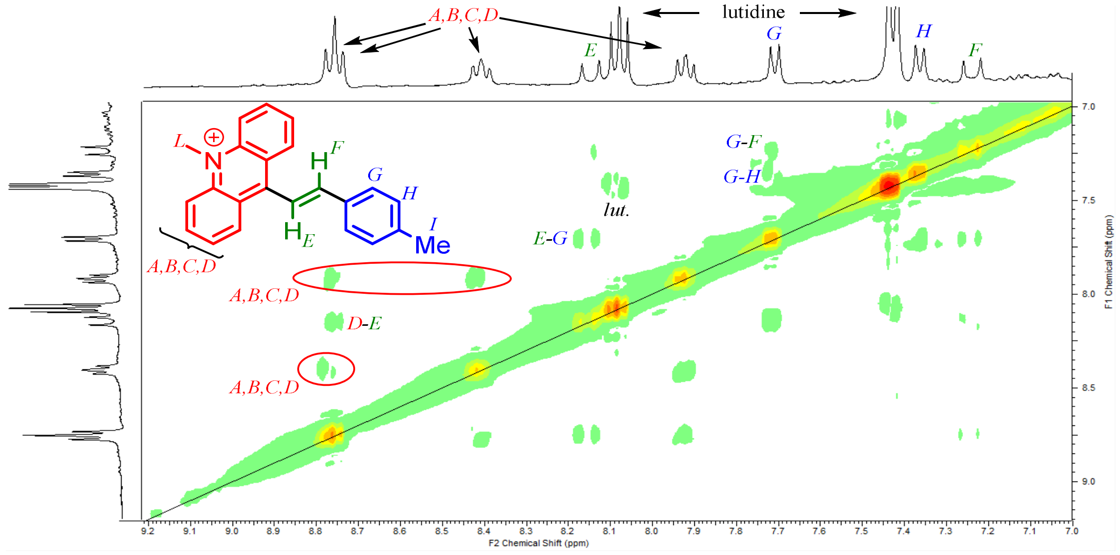

Figure S21. ^{1}H-^{1}H NOESY NMR spectra of the reaction with **8** and [2,6-lutidinium][$AlCl_4$] in d_2-DCM after hexane washing.

Single crystals of [**9**][$AlCl_4$] were isolated from the dichloromethane solution layered with hexane.

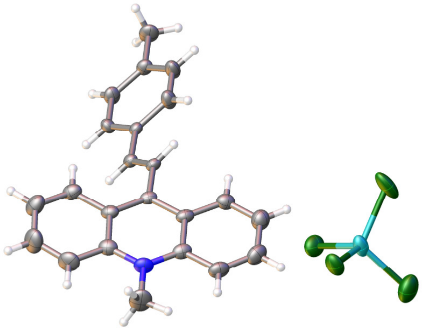

6.3 Reaction between 8 and [2,6-lutidinium][AlCl$_4$] with catalytic [1][BArCl]

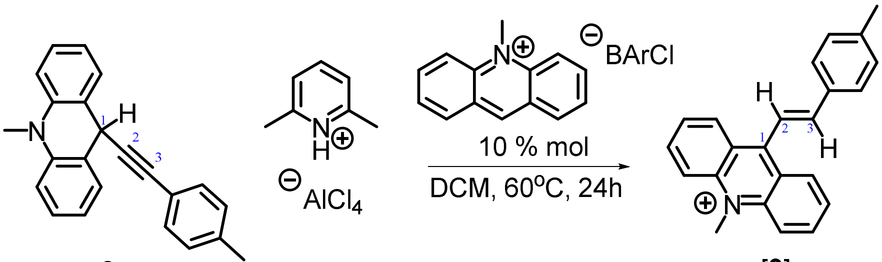

Under inert atmosphere, a J Youngs NMR tube was charged with **8** (15 mg, 0.046 mmol, 1.0 eq.) in *d*$_2$-DCM (0.5 mL), followed by the addition of [**1**][BArCl] (4 mg, 0.005 mmol, 0.1 eq.). On mixing, the solution changed from pale yellow to strong red. Then, 2,6-lutidinium tetrachloroaluminate (13 mg, 0.046 mmol, 1.0 eq.) was added. The initial monitoring of the reaction by multinuclear NMR spectroscopy revealed conversion of [**1**]$^+$ to **1-H**, along with a new *N*-methyl-acridinium resonance (pink signal in the following spectra). Upon heating at 60°C for 2 h, the ^{1}H NMR spectrum revealed full consumption of the starting material **8**, with [**9**]$^+$ observed as the major product (diagnostic ^{1}H NMR signals analogous to that observed in Section 6.2), along with an un-identified product that we tentatively assign to the corresponding allene.

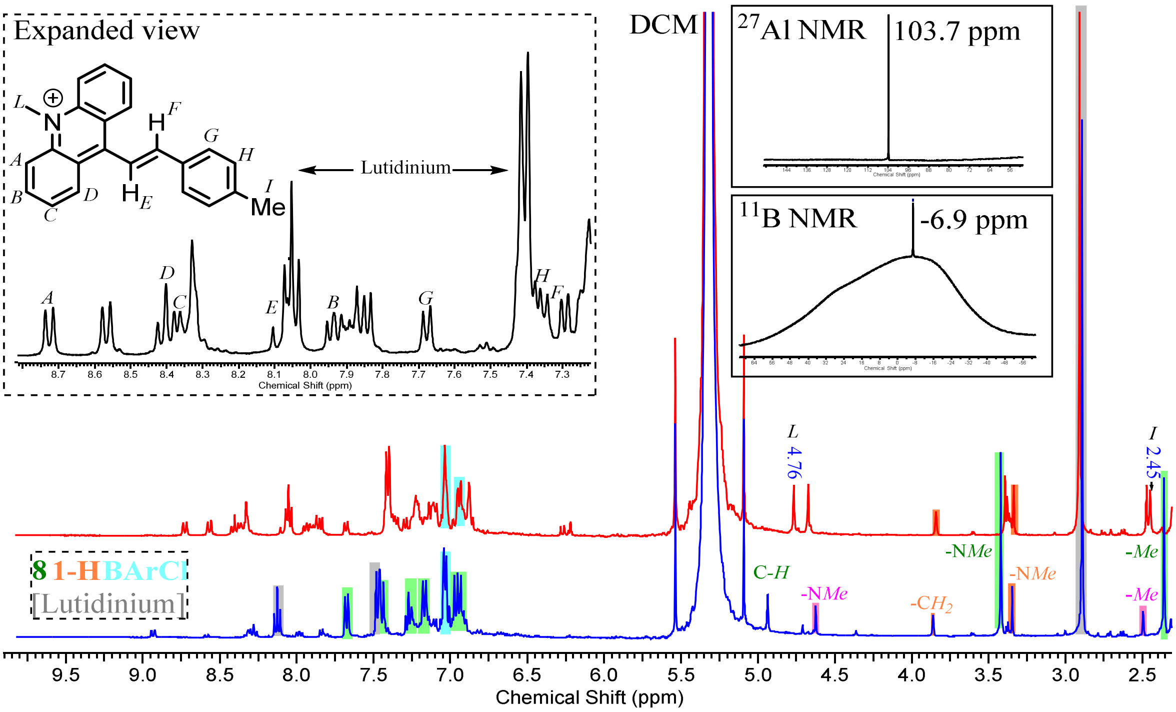

Figure S22. *In situ* ^{1}H-NMR spectra of the reaction with **8** and [2,6-lutidinium][AlCl$_4$] with catalytic [**1**][BArCl] in anhydrous *d*$_2$-DCM. Blue (t = 5 min, r.t.), red (after 2 h at 60°C). Inset, ^{27}Al-NMR and ^{11}B-NMR spectra after 2 h at 60°C.

7. HIA calculation coordinates of the Lewis acids

Calculations were performed using the Gaussian09[2] suite of programmes. Structures were optimised at the M06-2X/6-311G(d,p) level with PCM(Dichloromethane) solvation.[3] In all cases, structures were confirmed as minima by frequency analysis and the absence of imaginary frequencies. Structures and energies for Et_3B, Et_3BH and [**1**]$^+$ were taken from previous work.[1,4] Full Cartesian coordinates for the optimised geometries for the HIA determinations are provided below.

Compound [**5**]$^+$

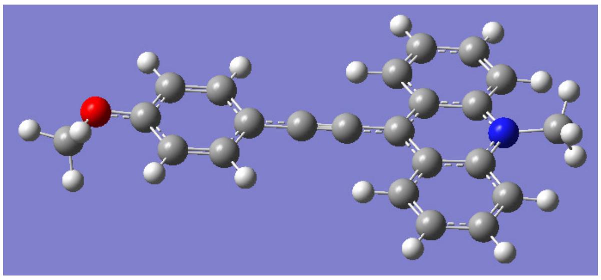

C	-1.905400	-2.258500	-1.338900
C	-0.572900	-2.165900	-1.064500
C	0.027200	-0.908800	-0.784300
C	-0.769900	0.269200	-0.821900
C	-2.156300	0.144800	-1.086700
C	-2.698800	-1.088400	-1.333200
C	1.400600	-0.804100	-0.462500
C	1.098900	1.605700	-0.158000
C	1.935300	0.456200	-0.107100
C	3.286700	0.595000	0.309400
H	3.909300	-0.290600	0.328700
C	3.780200	1.808500	0.687800
C	2.931000	2.938600	0.673800
C	1.628100	2.851800	0.260400
H	-2.360100	-3.218500	-1.544300
H	0.054100	-3.048400	-1.044300
H	-2.805000	1.006300	-1.058700
H	-3.763200	-1.164500	-1.517900
H	4.807300	1.908400	1.012500
H	3.311800	3.896200	1.007000
H	1.006800	3.733100	0.296100
C	-0.983600	2.713600	-0.828300
H	-1.512900	3.011200	0.077700
H	-0.318200	3.505500	-1.153800
H	-1.686900	2.532000	-1.633300
C	2.219300	-1.948600	-0.463900
C	2.922200	-2.935700	-0.465700
C	3.745300	-4.087500	-0.467300
C	5.121300	-3.979700	-0.188100
C	3.200800	-5.347100	-0.747400

C	5.919000	-5.100700	-0.194200
H	5.547000	-3.007700	0.030000
C	3.998400	-6.479800	-0.752700
H	2.142400	-5.434000	-0.961400
C	5.365700	-6.360100	-0.476600
H	6.979200	-5.040900	0.016000
H	3.552900	-7.440200	-0.969900
N	-0.195600	1.492200	-0.593100
O	6.225300	-7.390100	-0.458400
C	5.726300	-8.692000	-0.744000
H	5.301800	-8.733100	-1.750000
H	6.582600	-9.357300	-0.681300
H	4.974400	-8.989500	-0.009100

Total Energy: -1016.87671516 Hartrees

Compound **4** (hydride attack in β-position of [**5**]$^{+}$)

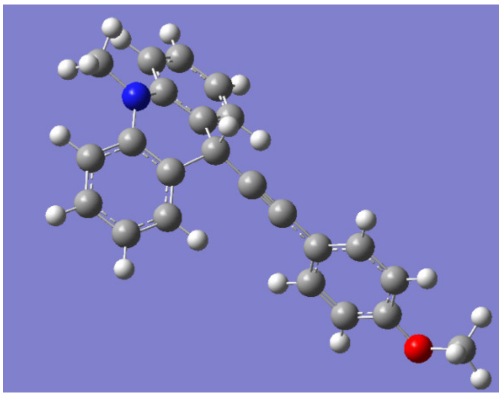

C	-2.509500	-1.370500	0.678400
C	-1.123100	-1.422200	0.805800
C	-0.312000	-0.578200	0.062900
C	-0.889100	0.361400	-0.807200
C	-2.279800	0.413900	-0.933800
C	-3.078700	-0.457300	-0.198800
C	1.112500	1.673500	-0.827200
C	1.755400	0.777100	0.041900
C	2.862500	1.190800	0.766400
H	3.342600	0.488000	1.437700
C	3.365900	2.481900	0.621800
C	2.744000	3.360500	-0.254700
C	1.616900	2.968900	-0.971600
H	-3.133700	-2.040600	1.256300
H	-0.661300	-2.136700	1.477800
H	-2.745100	1.141000	-1.585600
H	-4.155600	-0.404700	-0.307900
H	4.237800	2.790700	1.184900
H	3.123900	4.367800	-0.377400
H	1.127700	3.680800	-1.622600
C	-0.598000	2.072900	-2.548300

H	-1.154800	2.928900	-2.146700
H	0.208000	2.440000	-3.181900
H	-1.265000	1.474100	-3.166800
N	-0.033800	1.228800	-1.510700
C	1.207300	-0.641400	0.062300
H	1.498400	-1.099700	-0.895900
C	1.761800	-1.466000	1.133900
C	2.225400	-2.139900	2.018300
C	2.775400	-2.932000	3.078500
C	2.543600	-2.587000	4.419900
C	3.552000	-4.058700	2.802100
C	3.074400	-3.346900	5.442600
H	1.943000	-1.715100	4.649100
C	4.090700	-4.830300	3.825700
H	3.738100	-4.335100	1.771300
C	3.853100	-4.474300	5.154300
H	2.903100	-3.090900	6.480900
H	4.687100	-5.696700	3.574900
O	4.329700	-5.150800	6.224100
C	5.134200	-6.294200	5.980700
H	5.419400	-6.673100	6.958600
H	4.572800	-7.061500	5.440600
H	6.031800	-6.028800	5.415500

Total Energy: -1017.64300204 Hartrees

Compound **6** (hydride attack in δ-position of [**5**]$^+$)

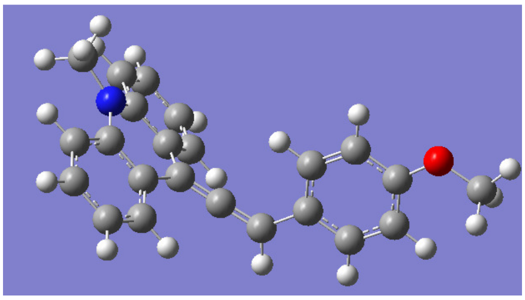

C	-2.837800	-2.884800	0.588100
C	-1.451700	-2.900900	0.560800
C	-0.718200	-1.764500	0.220100
C	-1.395200	-0.569500	-0.090000
C	-2.797800	-0.553800	-0.041800
C	-3.505300	-1.701100	0.285500
C	0.666100	0.692400	-0.057800
C	1.416600	-0.458000	0.252500
C	2.755300	-0.325800	0.620900
H	3.312100	-1.224900	0.862100
C	3.370000	0.915400	0.677200
C	2.624700	2.052400	0.377000
C	1.288000	1.947900	0.021600
H	-3.390500	-3.777800	0.851200

H	-0.908600	-3.808100	0.802700
H	-3.339400	0.360900	-0.238600
H	-4.587700	-1.661700	0.317500
H	4.411100	0.997700	0.962400
H	3.080000	3.034000	0.433800
H	0.724300	2.849300	-0.174100
C	-1.363100	1.716300	-1.006100
H	-1.778600	2.382900	-0.242100
H	-0.670600	2.278500	-1.629900
H	-2.168600	1.363800	-1.647400
C	0.761400	-1.782200	0.162800
N	-0.671600	0.571900	-0.436900
C	1.445500	-2.901100	0.057800
C	2.119600	-4.014600	-0.073800
H	2.424000	-4.554200	0.823400
C	2.513000	-4.625100	-1.360400
C	3.183000	-5.845000	-1.374200
C	2.230300	-4.010200	-2.589100
C	3.566000	-6.454700	-2.568000
H	3.413000	-6.337400	-0.435300
C	2.603400	-4.600300	-3.779100
H	1.711200	-3.057400	-2.598500
C	3.275100	-5.830400	-3.778800
H	4.084800	-7.402900	-2.535700
H	2.390300	-4.131600	-4.732200
O	3.598800	-6.327900	-4.997900
C	4.278800	-7.571400	-5.040200
H	3.673900	-8.365800	-4.593800
H	4.444200	-7.788100	-6.092400
H	5.241500	-7.510600	-4.524300

Total Energy: -1017.65358806 Hartrees

Compound [**2**]$^+$

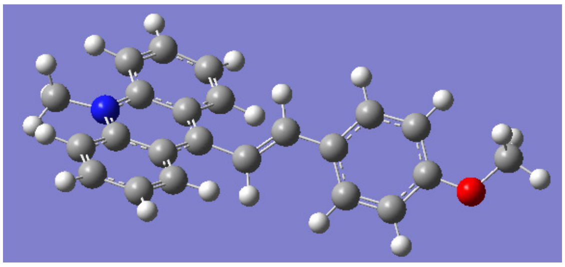

C	0.567100	-3.903800	0.438300
C	1.877800	-3.843400	0.067500
C	2.550800	-2.595900	-0.071700
C	1.831400	-1.399800	0.206100
C	0.475900	-1.487000	0.611600
C	-0.132200	-2.708900	0.716100
C	3.919900	-2.533600	-0.431400

C	3.806100	-0.099900	-0.135900
C	4.551400	-1.269600	-0.474200
C	5.906100	-1.106900	-0.893300
H	6.442200	-1.969000	-1.261300
C	6.516200	0.111800	-0.861700
C	5.798900	1.238200	-0.400700
C	4.476200	1.147200	-0.061200
H	0.070400	-4.859100	0.543800
H	2.428800	-4.757600	-0.103000
H	-0.081800	-0.604000	0.881000
H	-1.164700	-2.755200	1.040000
H	7.541800	0.219500	-1.188600
H	6.295200	2.198200	-0.327900
H	3.964000	2.028700	0.291200
C	1.675200	1.048800	0.248800
H	1.632200	1.363400	1.292100
H	2.123200	1.824600	-0.361400
H	0.676400	0.877100	-0.135500
C	4.627600	-3.774000	-0.739900
C	5.809700	-4.121400	-0.200100
H	4.097400	-4.471700	-1.377800
H	6.276200	-3.442700	0.510700
C	6.522500	-5.374300	-0.425600
C	7.648900	-5.663700	0.348200
C	6.127400	-6.314700	-1.394600
C	8.363000	-6.845500	0.188400
H	7.975900	-4.950700	1.097400
C	6.825300	-7.488500	-1.567000
H	5.270500	-6.121300	-2.028700
C	7.949600	-7.768000	-0.773800
H	9.226700	-7.033000	0.810900
H	6.529400	-8.215900	-2.312500
O	8.560700	-8.943300	-1.017400
N	2.463100	-0.188200	0.109500
C	9.711900	-9.267400	-0.249500
H	9.465700	-9.332700	0.813500
H	10.046100	-10.237100	-0.607600
H	10.501900	-8.527500	-0.401500

Total Energy: -1018.11389501 Hartrees

Compound **2-H1** (hydride attack in β-position of [**2**]$^+$)

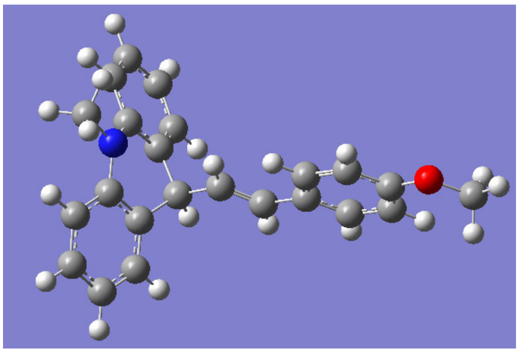

C	0.402000	-3.744000	0.848800
C	1.793600	-3.765100	0.842500
C	2.523500	-2.727600	0.278900
C	1.853700	-1.627100	-0.280600
C	0.454400	-1.601800	-0.269900
C	-0.258800	-2.659600	0.284400
C	4.032000	-2.773600	0.174400
C	3.865000	-0.310000	-0.236100
C	4.587100	-1.374600	0.328300
C	5.810400	-1.130900	0.937700
H	6.355100	-1.966900	1.365300
C	6.347600	0.152200	0.983800
C	5.638100	1.201500	0.412400
C	4.402100	0.981400	-0.187100
H	-0.155800	-4.562600	1.286200
H	2.328900	-4.608600	1.266900
H	-0.084300	-0.754400	-0.671900
H	-1.341800	-2.622300	0.284000
H	7.306500	0.327300	1.455400
H	6.037600	2.208400	0.441400
H	3.855800	1.821500	-0.594000
C	1.944400	0.472800	-1.561000
H	1.455000	1.206300	-0.907900
H	2.670700	0.986700	-2.189000
H	1.197300	0.025500	-2.214600
N	2.617200	-0.579300	-0.822000
H	4.434500	-3.417600	0.961000
C	4.438200	-3.338000	-1.167900
C	5.163000	-4.448200	-1.314200
H	4.094400	-2.769800	-2.030000
H	5.467500	-4.985700	-0.417400
C	5.602600	-5.052300	-2.582900
C	6.235500	-6.294300	-2.566700
C	5.420000	-4.433300	-3.830900
C	6.668100	-6.920600	-3.734200
H	6.395300	-6.793300	-1.616500
C	5.842300	-5.038600	-4.997000
H	4.948000	-3.459900	-3.892200

C	6.469800	-6.290300	-4.960400
H	7.152200	-7.885500	-3.669400
H	5.704100	-4.561700	-5.960000
O	6.848500	-6.799100	-6.159000
C	7.478100	-8.069400	-6.163000
H	6.814500	-8.837600	-5.755700
H	7.697000	-8.293400	-7.204000
H	8.409600	-8.049100	-5.590200

Total Energy: -1018.88609409 Hartrees

Compound **2-H3** (hydride attack in δ-position of [**2**]$^{+}$)

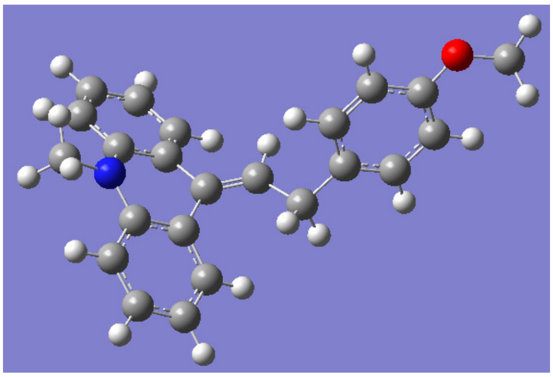

C	-0.189600	1.050600	4.852100
C	0.874300	1.022600	3.960000
C	0.782300	1.619500	2.703600
C	-0.399900	2.300500	2.350400
C	-1.468900	2.333500	3.255000
C	-1.362800	1.703200	4.488300
C	0.710600	3.388500	0.518500
C	1.923400	2.734300	0.824100
C	3.116900	3.248800	0.314900
H	4.051400	2.787000	0.608300
C	3.126900	4.343700	-0.540100
C	1.922100	4.949200	-0.878800
C	0.723900	4.489700	-0.347200
H	-0.100300	0.578000	5.822300
H	1.801000	0.533100	4.237600
H	-2.377000	2.867700	3.011600
H	-2.199600	1.744600	5.175400
H	4.063300	4.723400	-0.929200
H	1.910500	5.807200	-1.540500
H	-0.192600	5.011900	-0.584700
C	1.861300	1.543200	1.694100
C	2.639100	0.452900	1.582800
H	2.458300	-0.360300	2.281500
C	3.698200	0.173900	0.548100
H	3.514200	0.782400	-0.343000
H	4.688000	0.460700	0.921700

C	3.728100	-1.289100	0.165100
C	4.849000	-2.079100	0.381200
C	2.603500	-1.889800	-0.414700
C	4.872800	-3.431900	0.035100
H	5.733500	-1.639500	0.830500
C	2.606900	-3.227200	-0.765800
H	1.713300	-1.293700	-0.590800
C	3.745000	-4.010000	-0.541500
H	5.767100	-4.010600	0.222200
H	1.740500	-3.695100	-1.217600
O	3.653500	-5.313000	-0.916100
C	-1.747800	3.444300	0.635700
H	-2.533000	2.734400	0.889700
H	-1.993900	4.421100	1.069200
H	-1.720200	3.532900	-0.448900
C	4.786800	-6.136100	-0.702300
H	4.511900	-7.125600	-1.058800
H	5.650700	-5.772100	-1.266200
H	5.040800	-6.188800	0.360500
N	-0.473000	2.928500	1.101900

Total Energy: -1018.88819019 Hartrees

Vinyl cation (for HIA compare its energy with that of **2-H1**)

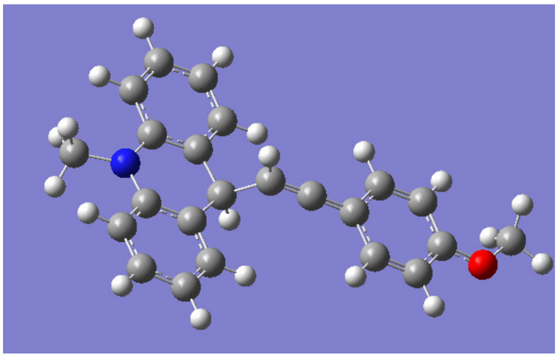

C	-3.286200	-4.668000	-1.079200
C	-2.049500	-4.108800	-0.794900
C	-1.907900	-2.759300	-0.480300
C	-3.038700	-1.924500	-0.475100
C	-4.295300	-2.506000	-0.718900
C	-4.412500	-3.854100	-1.018900
C	-0.536000	-2.228400	-0.126200
C	-1.726500	-0.004300	0.215800
C	-0.542000	-0.760400	0.240900
C	0.651500	-0.156800	0.629700
H	1.558300	-0.753100	0.622500
C	0.706100	1.171500	1.023400
C	-0.475700	1.904600	1.051300

C	-1.673800	1.329000	0.658900
H	-3.371800	-5.718300	-1.325500
H	-1.160700	-4.731900	-0.813100
H	-5.193100	-1.908100	-0.654900
H	-5.396800	-4.268800	-1.201200
H	1.645800	1.620400	1.317900
H	-0.472200	2.934900	1.386500
H	-2.577900	1.917900	0.716800
C	-4.045400	0.321900	-0.548000
H	-4.710400	0.467000	0.309100
H	-3.670000	1.288400	-0.877000
H	-4.614600	-0.097700	-1.374400
N	-2.922700	-0.554500	-0.242000
H	0.154500	-2.401100	-0.955900
C	-0.010500	-3.025100	1.084400
H	-0.597600	-2.976300	2.000200
C	1.064300	-3.728100	1.062800
C	2.195800	-4.456000	1.001100
C	2.160100	-5.837000	0.599400
C	3.455400	-3.858500	1.339300
C	3.308400	-6.556100	0.536600
H	1.206200	-6.285200	0.352200
C	4.609000	-4.581000	1.277000
H	3.473200	-2.820900	1.648900
C	4.547900	-5.941300	0.872100
H	3.323900	-7.596000	0.238600
H	5.552500	-4.121100	1.532400
O	5.593700	-6.715800	0.779800
C	6.903000	-6.213200	1.098100
H	7.574900	-7.048400	0.933400
H	7.157100	-5.387900	0.433100
H	6.937500	-5.900100	2.141700

Total Energy: -1018.04106202 Hartrees

8. Crystal structures of [2]$^+$, [3]$^+$, [7]$^+$, [9]$^+$

Crystallographic data for compounds [**2**]$^+$, [**3**]$^+$ and [**7**]$^+$ were recorded on an Agilent Supernova diffractometer, at 150 K with Mo Kα radiation (mirror monochromator, λ =0.7107). The CrysAlisPro[5] software package was used for data collection, cell refinement and data reduction. Data for [**9**]$^+$ was collected on an Oxford Diffraction Xcalibur 2 at 150 K with Mo Kα radiation (mirror monochromator, λ =0.7107). The CrysAlisPro[5] software package was used for data collection, cell refinement and data reduction. For all data sets the CrysAlisPro software package was used for empirical absorption corrections, which were applied using spherical harmonics, implemented in SCALE3 ABSPACK scaling algorithm. All further data processing was undertaking within the Olex2 software.[6] The molecular structures of compounds [**3**]$^+$ was solved with the Superflip structure solution program using Charge Flipping methods,[7-9] while the structure of [**2**]$^+$ and [**7**]$^+$ were solved using the ShelXS[10] structure solution program using direct methods and the structure of [**9**]$^+$ was solved with ShelXT[11] structure solution program using Intrinsic Phasing. All structures were refined with the SHELXL[12] refinement package using Least Squares minimisation against F2. Non-hydrogen atoms were refined anisotropically. Hydrogen atoms were all located in a difference map and repositioned geometrically.

Compound	[2][BArCl]-CH_2Cl_2	[3][BArCl]
**CCDC code**	1550739	1550716
**Empirical formula**	$C_{48}H_{34}BCl_{10}NO$	$C_{47}H_{32}BCl_8NO$
**Formula weight**	1006.07	921.14
**Temperature/K**	293(2)	150.01(11)
**Crystal system**	triclinic	monoclinic
**Space group**	P-1	$P2_1/c$
**a/Å**	12.230(2)	10.3102(8)
**b/Å**	13.175(3)	18.5763(13)
**c/Å**	14.826(3)	22.1935(19)
**α/°**	94.09(3)	90
**β/°**	90.63(3)	91.330(7)
**γ/°**	109.53(3)	90
**Volume/Å^3**	2244.1(9)	4249.5(6)
**Z**	2	4
**ρ_{calc}g/cm^3**	1.489	1.440
**μ/mm^{-1}**	0.661	0.569
**F(000)**	1024.0	1880.0
**Crystal size/mm^3**	0.3 × 0.2 × 0.1	0.3 × 0.1 × 0.1
**Radiation**	MoKα (λ = 0.71073)	MoKα (λ = 0.71073)
**2Θ range for data collection/°**	6.44 to 52.744	6.832 to 56.636
**Index ranges**	$-15 \leq h \leq 14$, $-16 \leq k \leq 16$, $-18 \leq l \leq 18$	$-13 \leq h \leq 11$, $-22 \leq k \leq 24$, $-27 \leq l \leq 22$
**Reflections collected**	18516	18158
**Independent reflections**	9174 [R_{int} = 0.0438, R_{sigma} = 0.0777]	9173 [R_{int} = 0.0437, R_{sigma} = 0.0890]
**Data/restraints/parameters**	9174/0/560	9173/54/535
**Goodness-of-fit on F^2**	1.025	1.107
**Final R indexes [I>=2σ (I)]**	R_1 = 0.0616, wR_2 = 0.1484	R_1 = 0.1460, wR_2 = 0.3144
**Final R indexes [all data]**	R_1 = 0.0944, wR_2 = 0.1745	R_1 = 0.2043, wR_2 = 0.3424
**Largest diff. peak/hole / e Å^{-3}**	0.74/-0.81	1.25/-1.08

Compound	[7][I]-($CHCl_3$)$_2$	[9][$AlCl_4$]
CCDC code	1550721	1550723
Empirical formula	$C_{39}H_{33}Cl_6IN_2O$	$C_{23}H_{20}AlCl_4N$
Formula weight	885.27	479.18
Temperature / K	149.9(4)	150
Crystal system	orthorhombic	monoclinic
Space group	Pbca	$P2_1/c$
a / Å	12.9340(4)	13.0390(7)
b / Å	19.8533(6)	7.3088(5)
c / Å	29.6855(10)	23.9159(14)
α / °	90	90
β / °	90	97.455(5)
γ / °	90	90
Volume / $Å^3$	7622.7(4)	2259.9(2)
Z	8	4
ρ_{calc} gcm^{-3}	1.543	1.408
M / mm^{-1}	1.295	0.573
F(000)	3552.0	984.0
Crystal size / mm^3	0.4 × 0.3 × 0.1	0.9 × 0.6 × 0.3
Radiation	MoKα (λ = 0.71073)	MoKα (λ = 0.71073)
2Θ range for data collection / °	6.612 to 58.324	6.736 to 58.394
Index ranges	$-15 \le h \le 17$, $-26 \le k \le 26$, $-39 \le l \le 40$	$-17 \le h \le 17$, $-7 \le k \le 9$, $-28 \le l \le 32$
Reflections collected	45640	12530
Independent reflections	9303 [R_{int} = 0.0567, R_{sigma} = 0.0548]	5292 [R_{int} = 0.0492, R_{sigma} = 0.0717]
Data/restraints/parameters	9303/0/445	5292/150/497
Goodness-of-fit on F^2	1.050	1.078
Final R indexes [I>=2σ (I)]	R_1 = 0.0415, wR_2 = 0.0724	R_1 = 0.0657, wR_2 = 0.1581
Final R indexes [all data]	R_1 = 0.0742, wR_2 = 0.0841	R_1 = 0.1130, wR_2 = 0.1866
Largest diff. peak/hole / e $Å^{-3}$	0.61/-0.55	0.38/-0.34

Compound **[2][BArCl]-CH_2Cl_2**

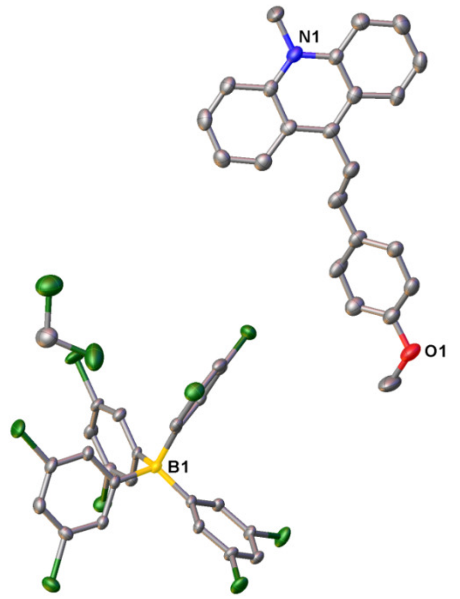

Figure S23: ORTEP representation of the molecular structure of compound **[2][BArCl]-CH_2Cl_2**, with thermal ellipsoids set at the 50 % level. H atoms are omitted for clarity.

Compound **[3][BArCl]**

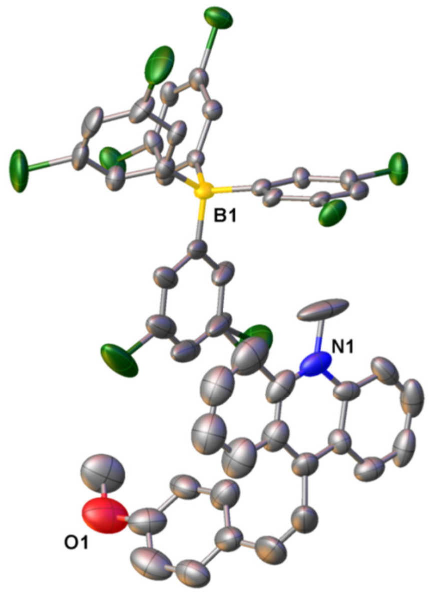

Figure S24: ORTEP representation of the molecular structure of compound **[3][BArCl]**, with thermal ellipsoids set at the 50 % level. H atoms are omitted for clarity.

Due to low quality of the data recorded for compound **[3][BArCl]**, the C-C bonds in the molecular contain large uncertainties.

Compound **[7][I]-($CDCl_3$)$_2$**

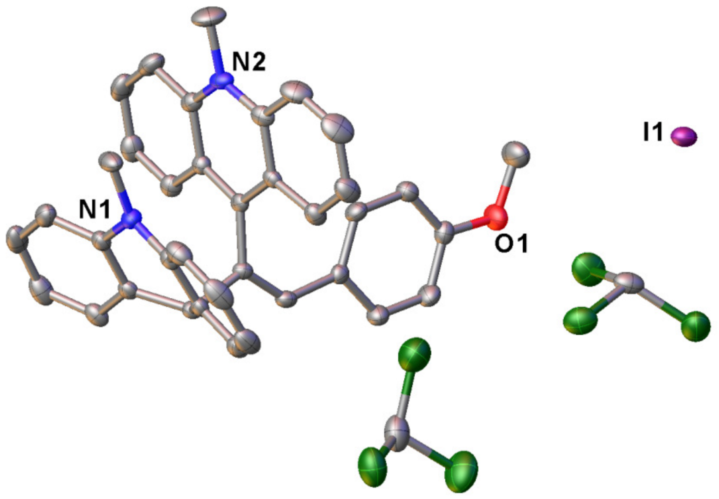

Figure S25: ORTEP representation of the molecular structure of compound **[7][I]-($CDCl_3$)$_2$**, with thermal ellipsoids set at the 50 % level. H atoms are omitted for clarity.

Compound **[9][$AlCl_4$]**

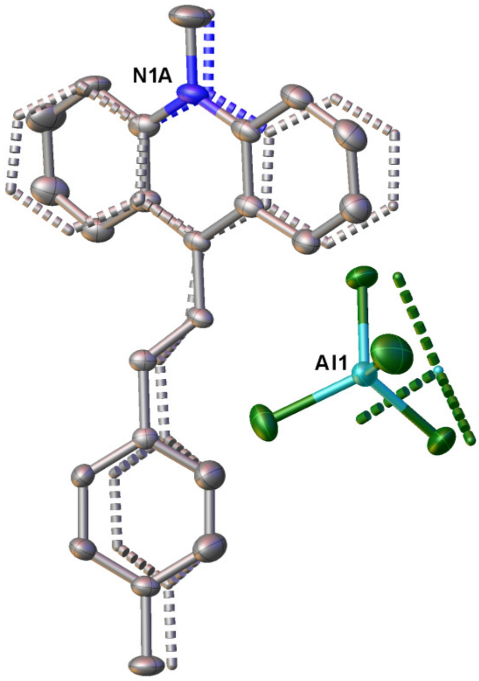

Figure S26: ORTEP representation of the molecular structure of compound **[9][$AlCl_4$]**, with thermal ellipsoids set at the 50 % level. H atoms are omitted for clarity.

The molecular structure of compound **[9][$AlCl_4$]** displays disorder across the entire molecule. The molecule is found to occupy two planes within the unit cell, with the occupancies refined to 0.73(1) and 0.27(1). The thermal ellipsoids of the atoms within the secondary disordered unit have been restrained with SIMU due to the level of disorder present.

9. References

1 E. R. Clark and M. J. Ingleson, *Angew. Chem. Int. Ed.* **2014**, *53*, 11306-11309

2 Gaussian 09, Revision C1, Frisch, M. J.; Trucks, G. W.; Schlegel, H. B.; Scuseria, G. E.; Robb, M. A.; Cheeseman, J. R.; Scalmani, G.; Barone, V.; Mennucci, B.; Petersson, G. A.; Nakatsuji, H.; Caricato, M.; Li, X.; Hratchian, H. P.; Izmaylov, A. F.; Bloino, J.; Zheng, G.; Sonnenberg, J. L.; Hada, M.; Ehara, M.; Toyota, K.; Fukuda, R.; Hasegawa, J.; Ishida, M.; Nakajima, T.; Honda, Y.; Kitao, O.; Nakai, H.; Vreven, T.; Montgomery, Jr., J. A.; Peralta, J. E.; Ogliaro, F.; Bearpark, M.; Heyd, J. J.; Brothers, E.; Kudin, K. N.; Staroverov, V. N.; Kobayashi, R.; Normand, J.; Raghavachari, K.; Rendell, A.; Burant, J. C.; Iyengar, S. S.; Tomasi, J.; Cossi, M.; Rega, N.; Millam, J. M.; Klene, M.; Knox, J. E.; Cross, J. B.; Bakken, V.; Adamo, C.; Jaramillo, J.; Gomperts, R.; Stratmann, R. E.; Yazyev, O.; Austin, A. J.; Cammi, R.; Pomelli, C.; Ochterski, J. W.; Martin, R. L.; Morokuma, K.; Zakrzewski, V. G.; Voth, G. A.; Salvador, P.; Dannenberg, J. J.; Dapprich, S.; Daniels, A. D.; Farkas, Ö.; Foresman, J. B.; Ortiz, J. V.; Cioslowski, J.; Fox, D. J. Gaussian, Inc., Wallingford CT, **2009**.

3 http://comp.chem.umn.edu/info/DFT.htm.

4 E. R. Clark, A. Del Grosso and M. J. Ingleson, Chem. Eur. J., 2013, 19, 2462-2466

5 CrysAlisPro, Agil. Technol. Version 1.1 71.35.19 (release 27-10-2011 CrysAlis171.NET) (compiled Oct 27 2011,150211)

6 O. V. Dolomanov, L. J. Bourhis, R. J. Gildea, J. A. K. Howard and H. Puschmann, *J. Appl. Crystallogr.* **2009**, *42*, 339-341

7 L. Palatinus and G. Chapuis, *J. Appl. Crystallogr.*, **2007**, *40*, 786-790.

8 L. Palatinus and A. van der Lee, *J. Appl. Crystallogr.*, **2008**, *41*, 975-984.

9 L. Palatinus, S. J. Prathapa and S. van Smaalen, *J. Appl. Crystallogr.*, **2012**, *45*, 575-580.

10 G. M. Sheldrick, *Acta Cryst.* **2008**, *A64*, 112-122.

11 G. M. Sheldrick, *Acta Cryst.* **2015**, *A71*, 3-8.

12 G. M. Sheldrick, *Acta Cryst.* **2015**, *C71*, 3-8.

10. NMR spectra of al compounds

^{1}H and $^{13}C\{^{1}H\}$-NMR spectra of 9-((4-methoxyphenyl)ethynyl)acridine in $CDCl_3$

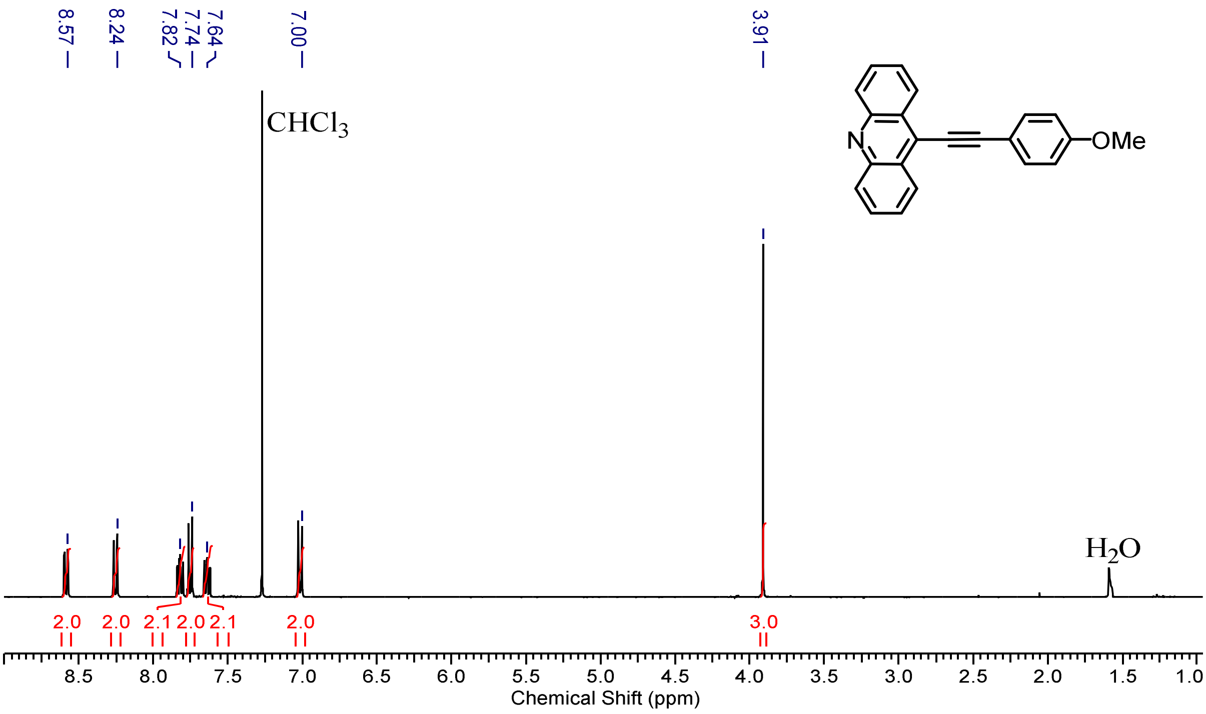

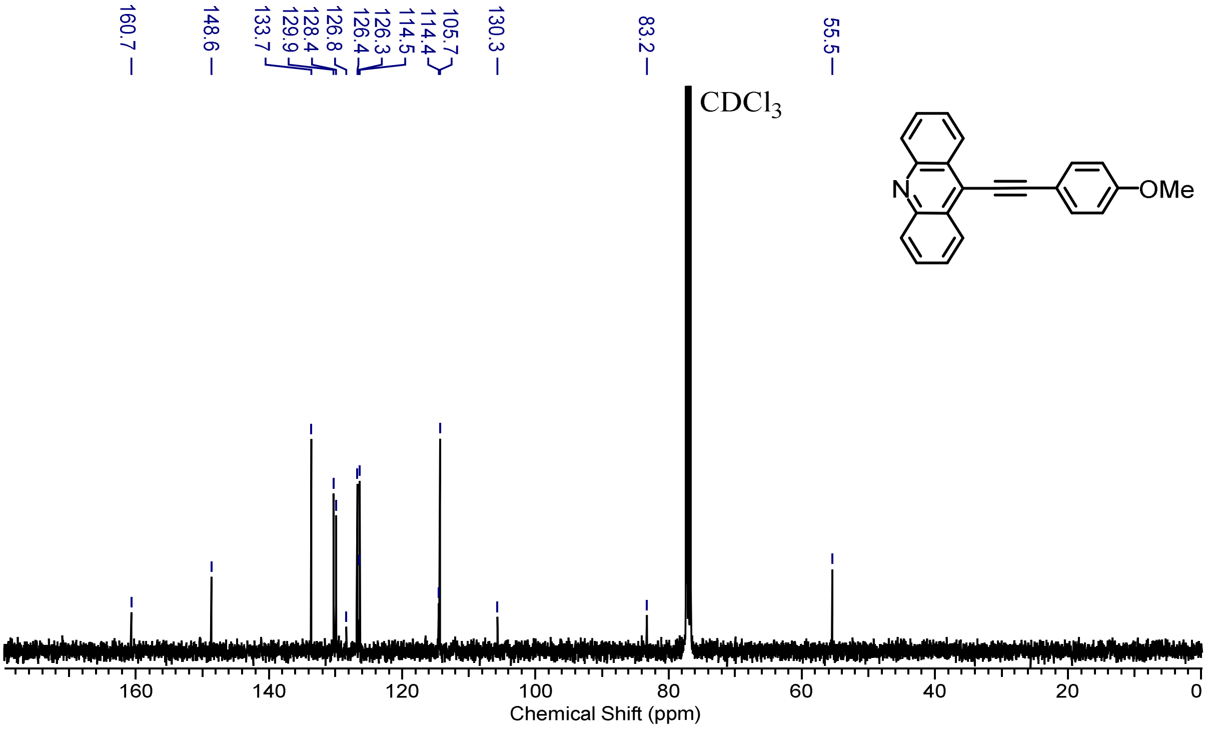

^{1}H and $^{13}C\{^{1}H\}$-NMR spectra of *N*-methyl-9-((4-methoxyphenyl)ethynyl)acridinium iodide [**5**][I] in $CDCl_3$

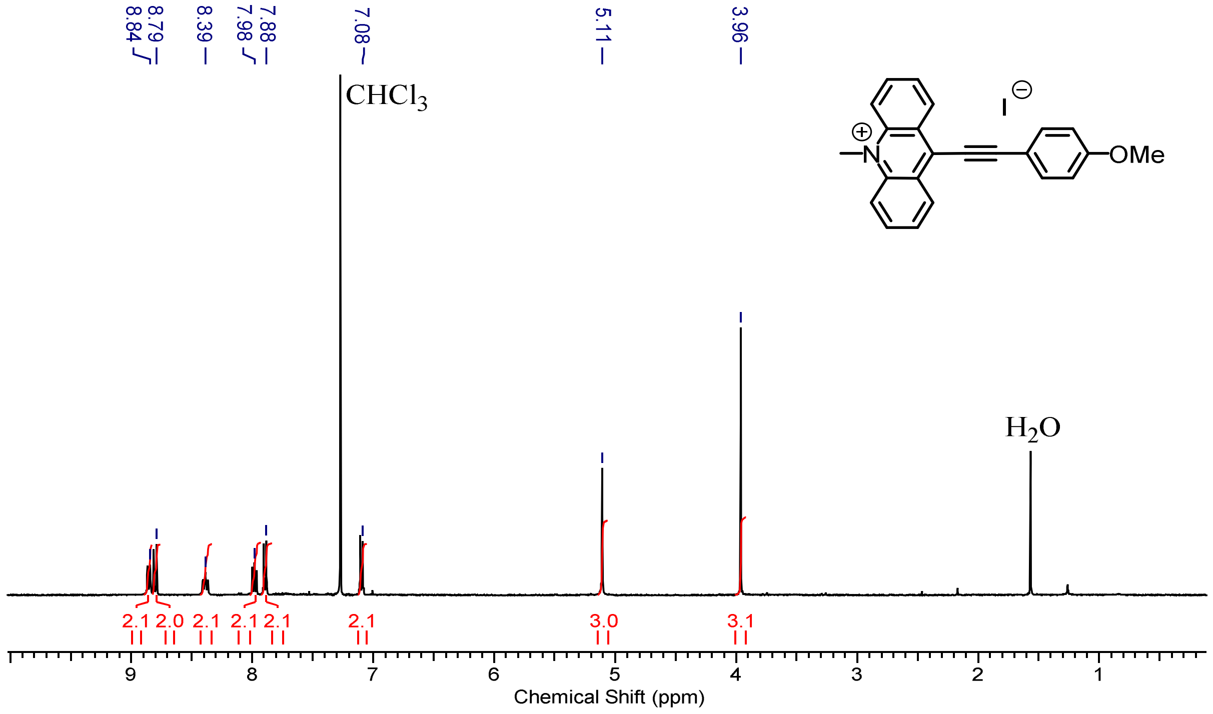

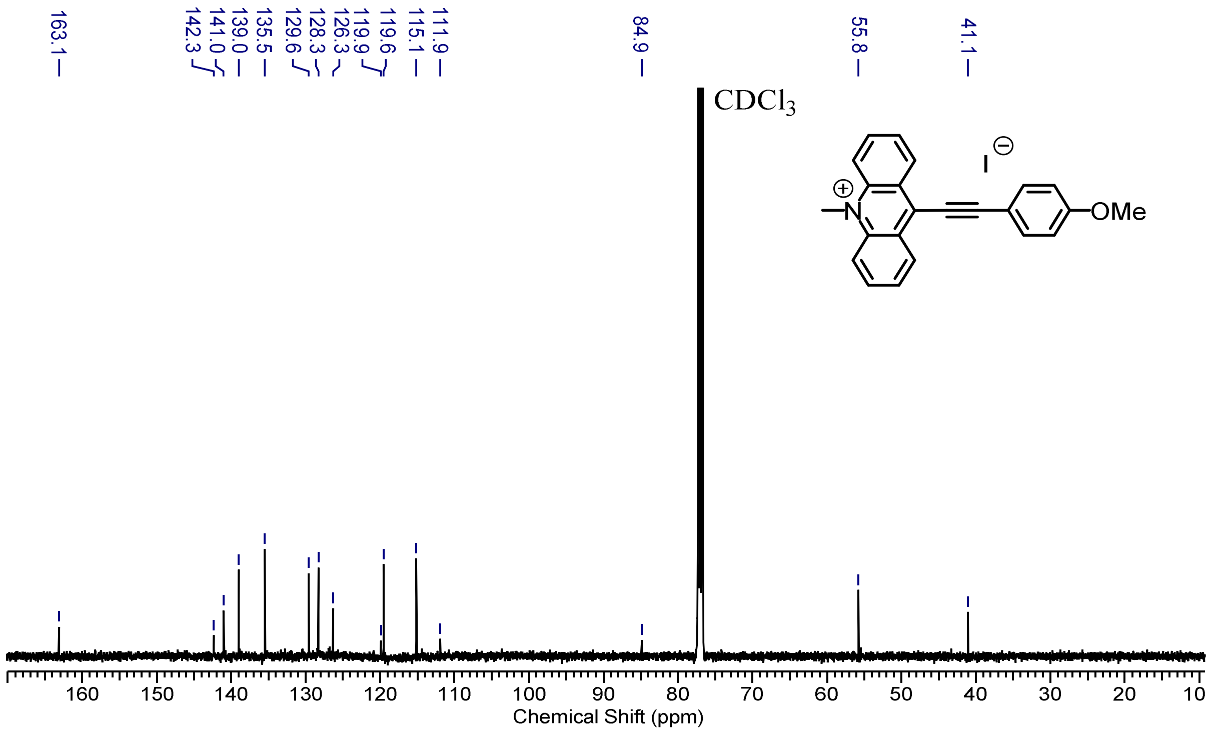

^1H and $^{13}C\{^1H\}$-NMR spectra of *N*-methyl-9-((4-methoxyphenyl)ethynyl)acridane **4** in $CDCl_3$

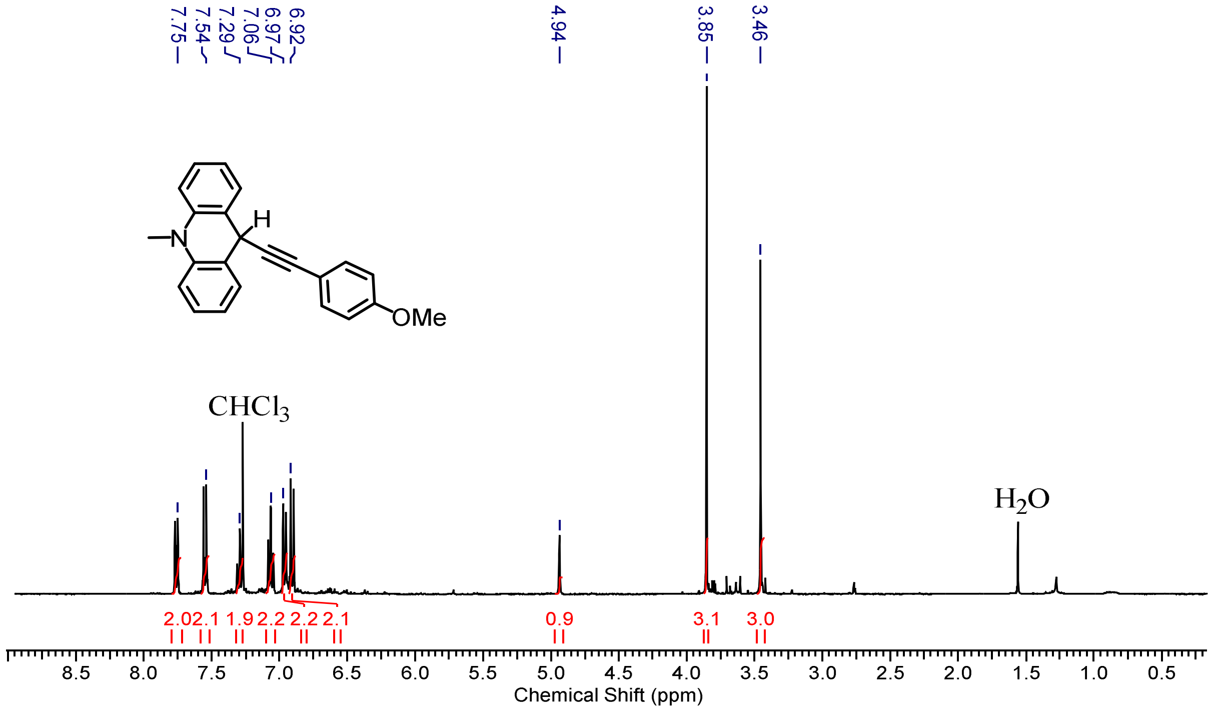

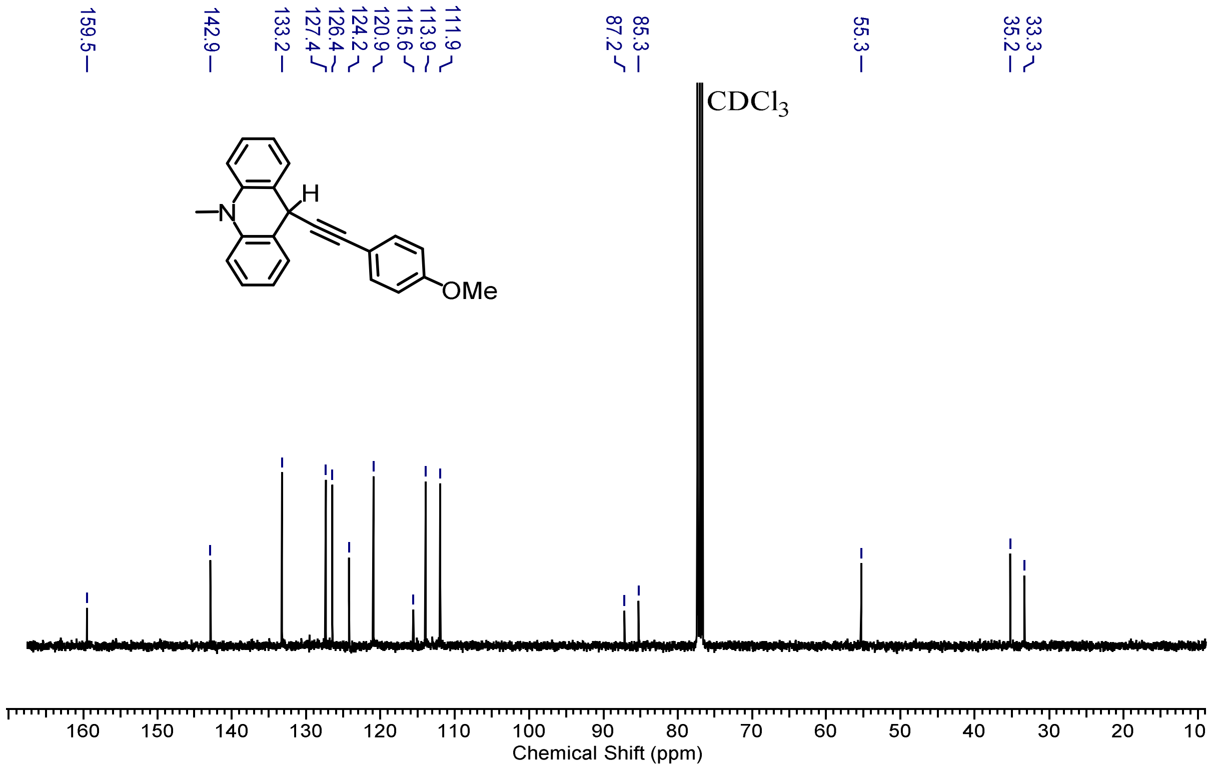

^{1}H, $^{13}C\{^{1}H\}$ and ^{2}H-NMR spectra of *N*-methyl-9-((4-methoxyphenyl)ethynyl)-9-deuterium-acridane **4D** in CD_2Cl_2

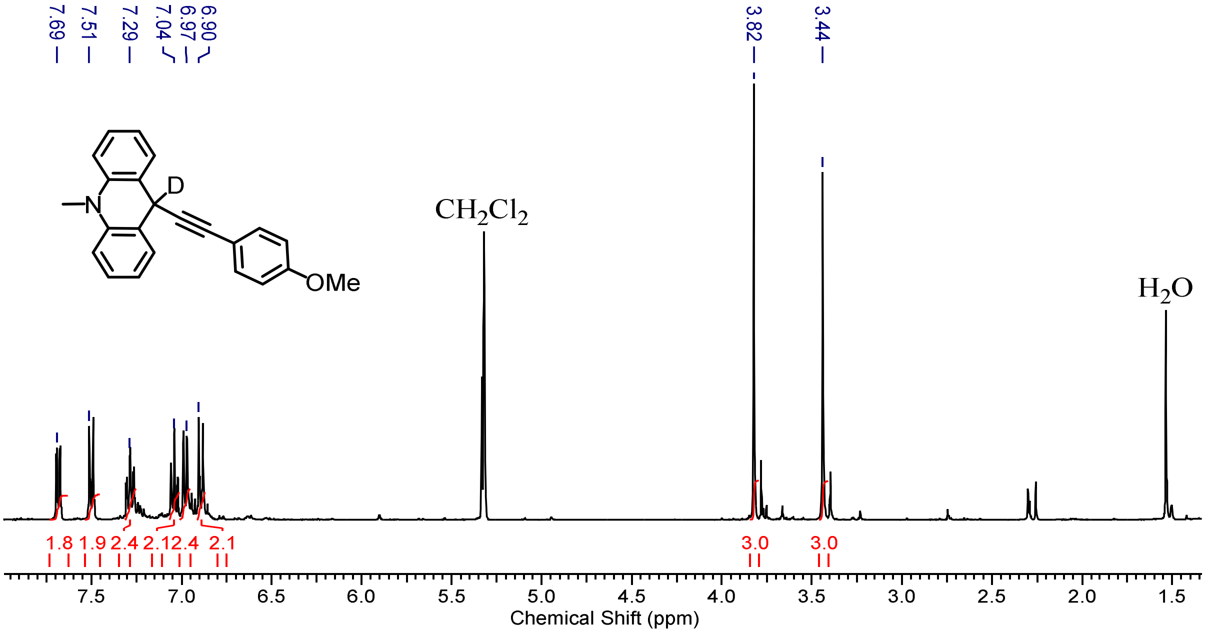

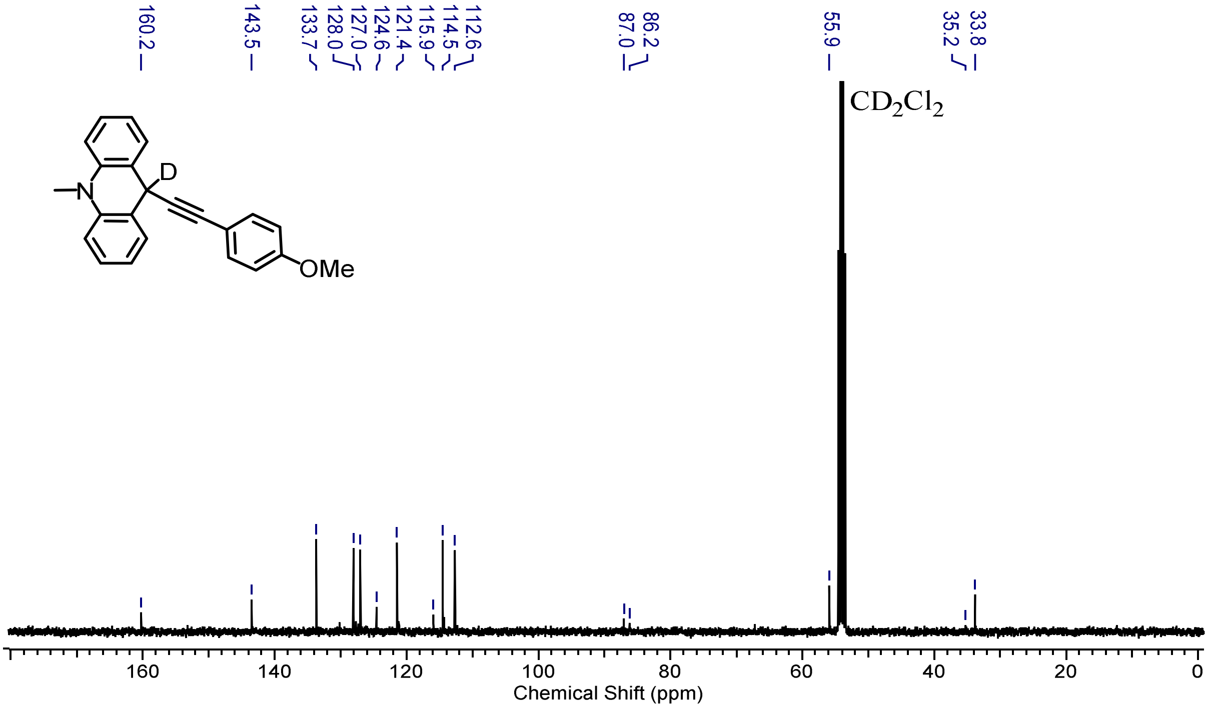

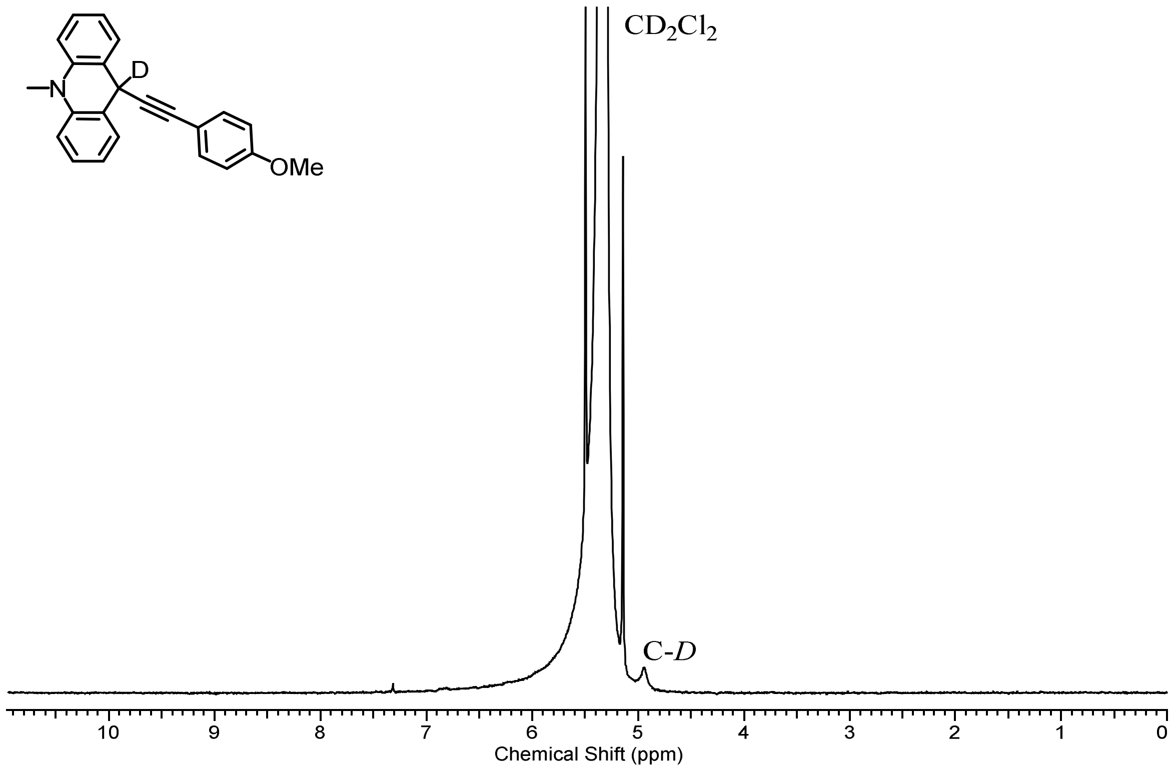
D
N
OMe
CD_2Cl_2
C-*D*
10
9
8
7
6
5
4
3
2
1
0
Chemical Shift (ppm)

^{1}H and $^{13}C\{^{1}H\}$-NMR spectra of 9-(p-tolylethynyl)acridine in $CDCl_3$

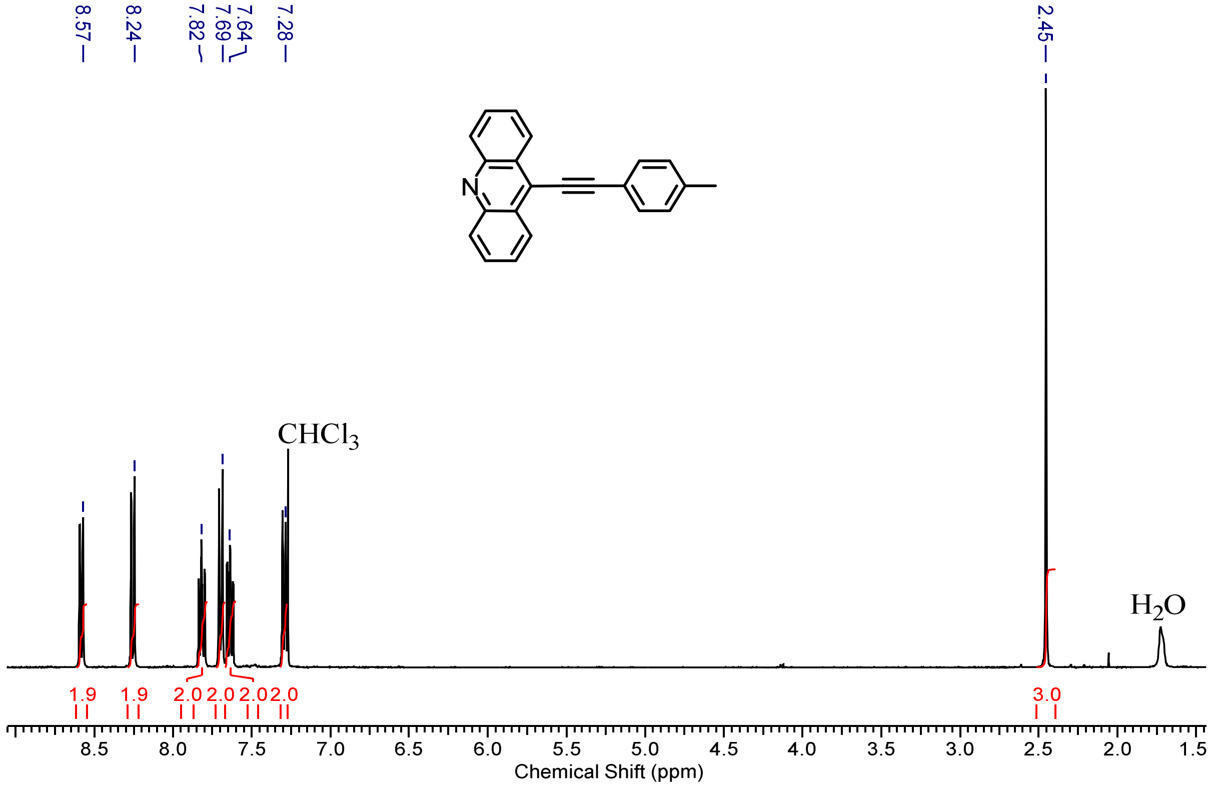

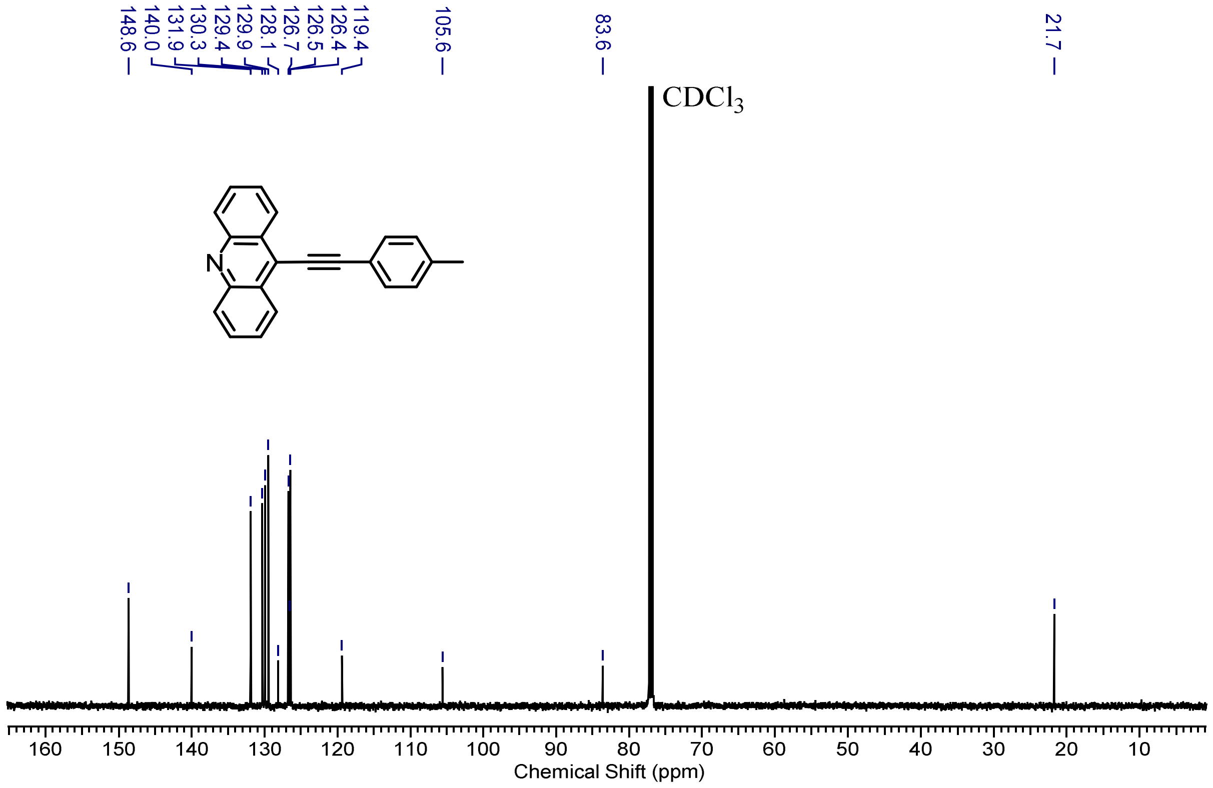

^{1}H and ^{13}C{^{1}H}-NMR spectra of *N*-methyl-9-(p-tolylethynyl)acridinium iodide in d_6-DMSO

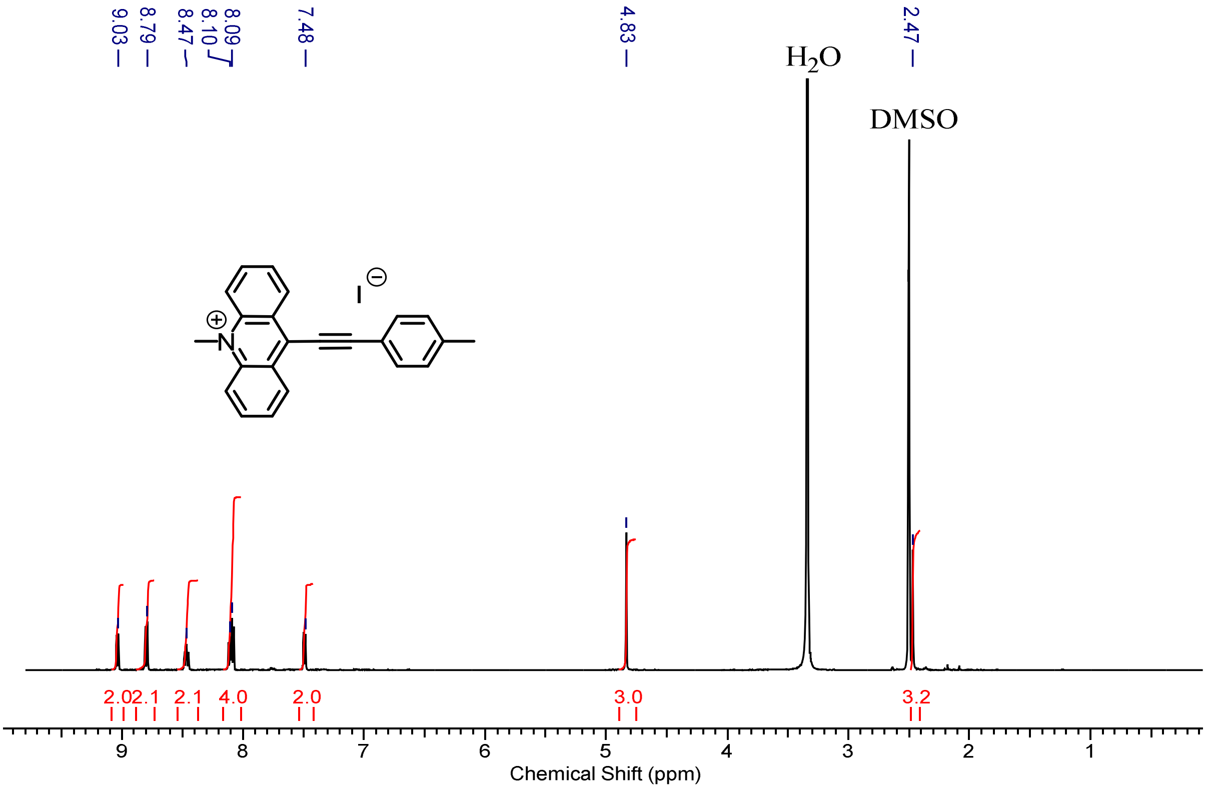

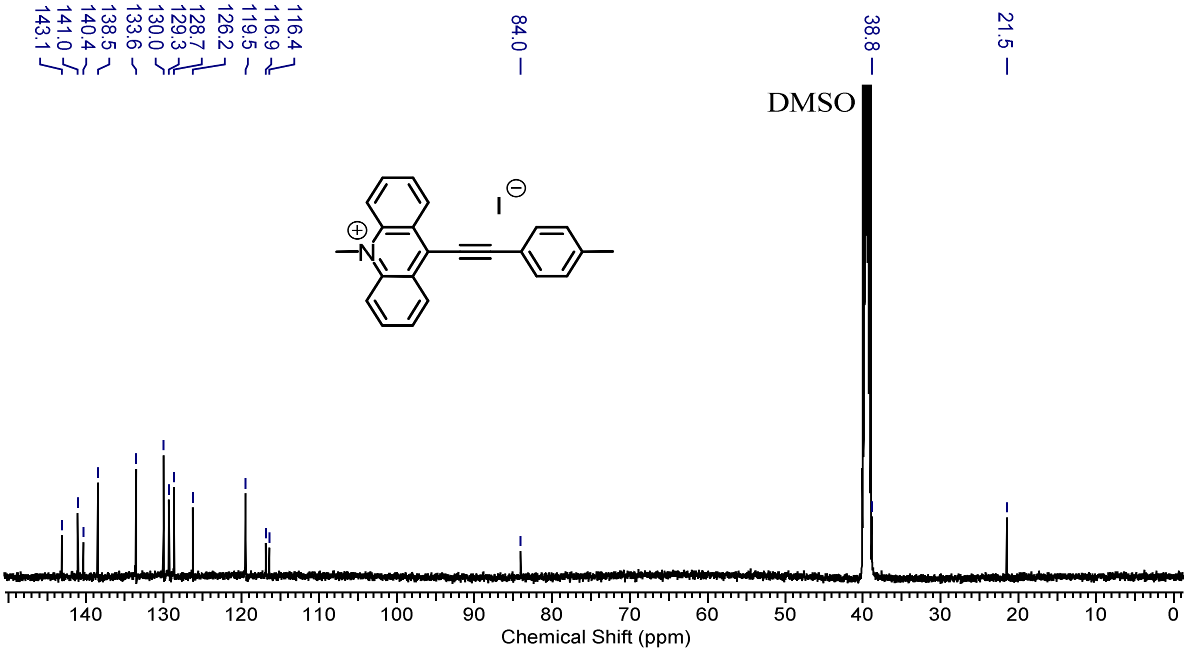

^{1}H and ^{13}C{^{1}H}-NMR spectra of *N*-methyl-9-(p-tolylethynyl)acridane **8** in $CDCl_3$

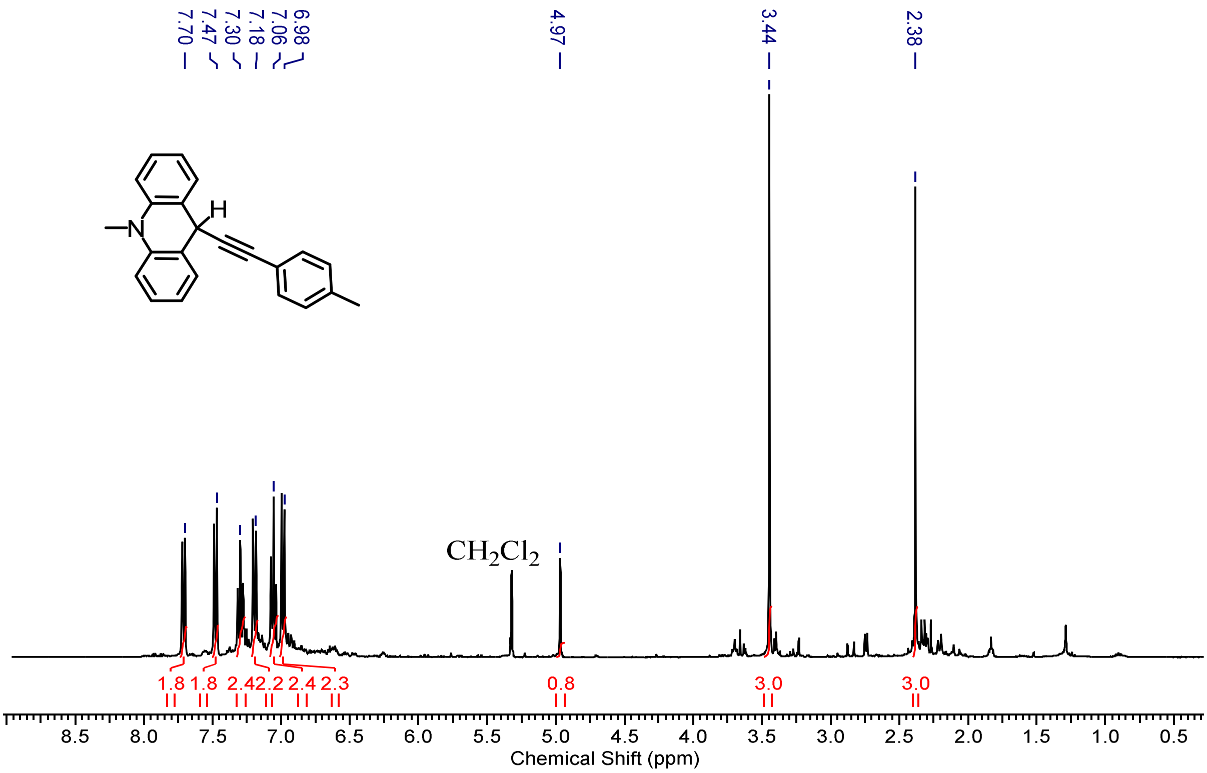

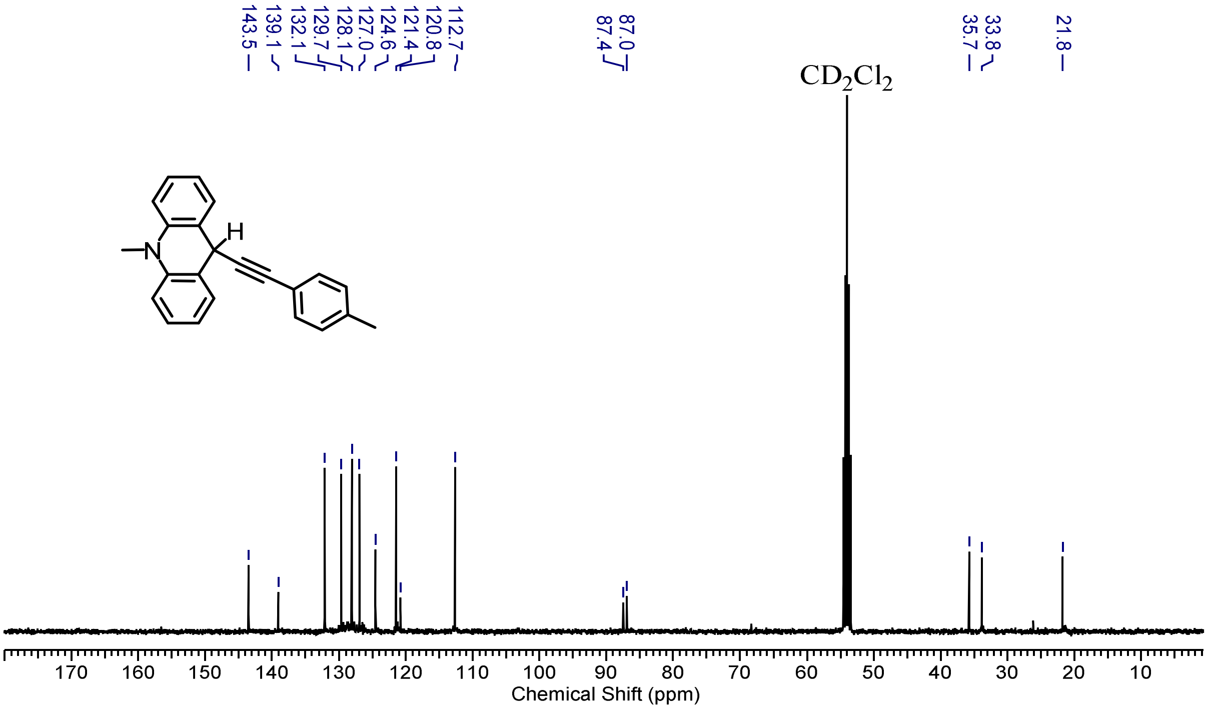

^{1}H and $^{13}C\{^{1}H\}$-NMR spectra of *N*-methyl-acridinium iodide in d_6-DMSO

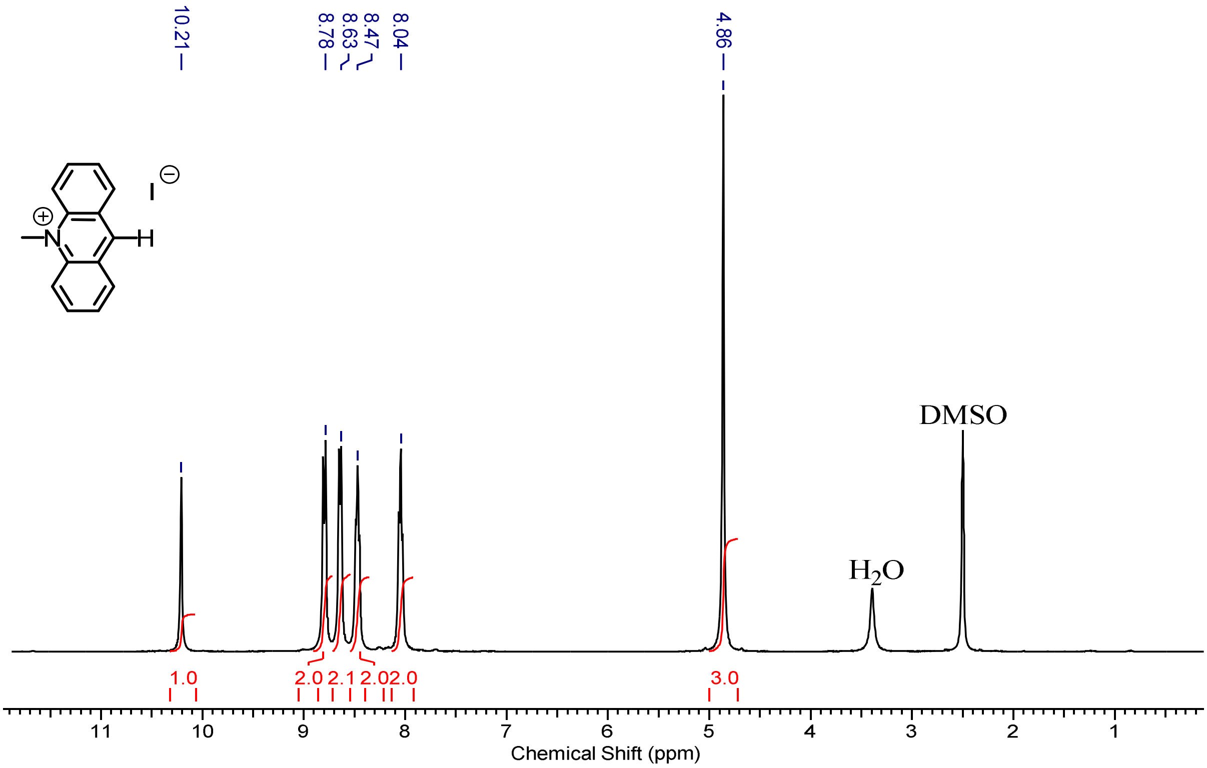

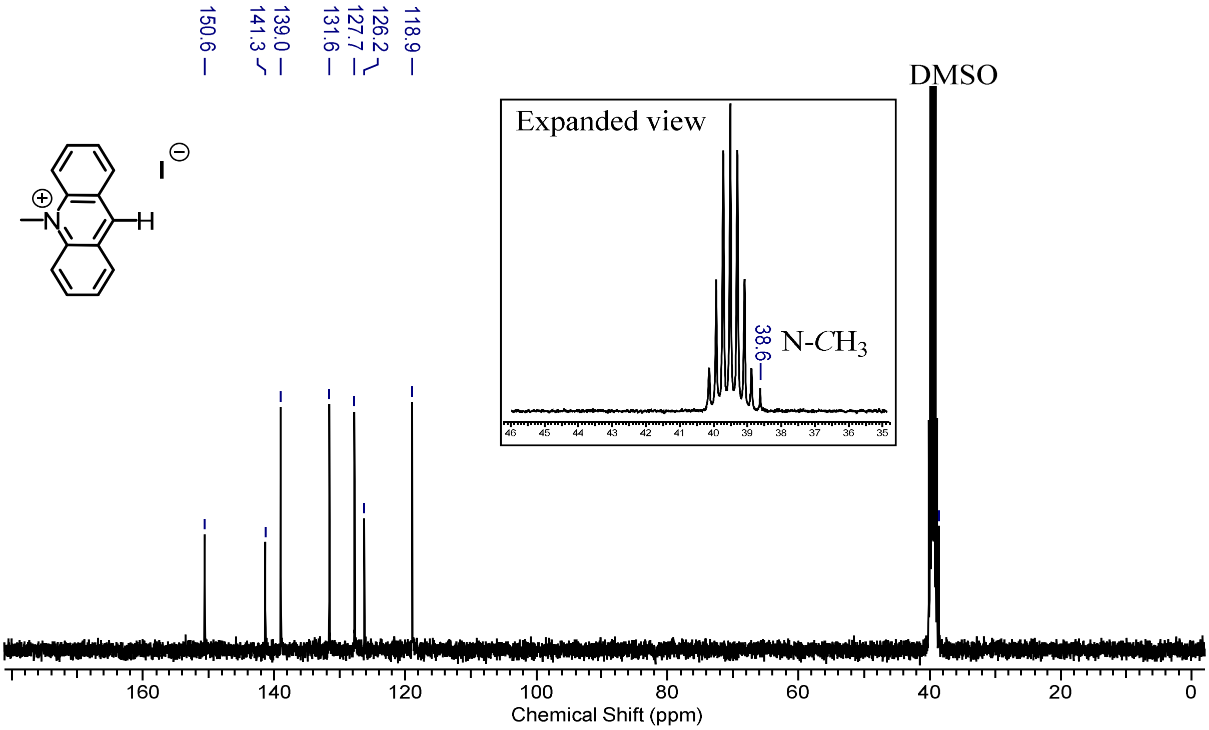

^{1}H and $^{13}C\{^{1}H\}$-NMR spectra of *N*-methyl-acridane **1**-H in $CDCl_3$

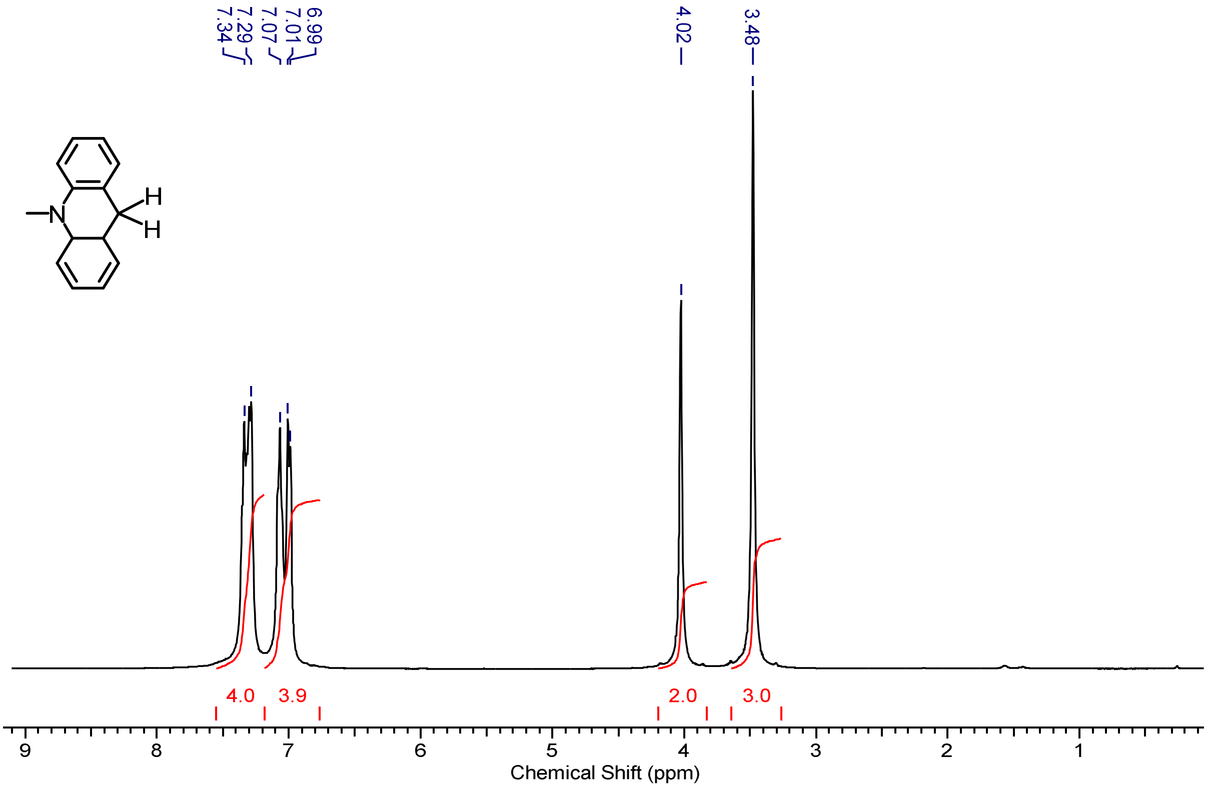

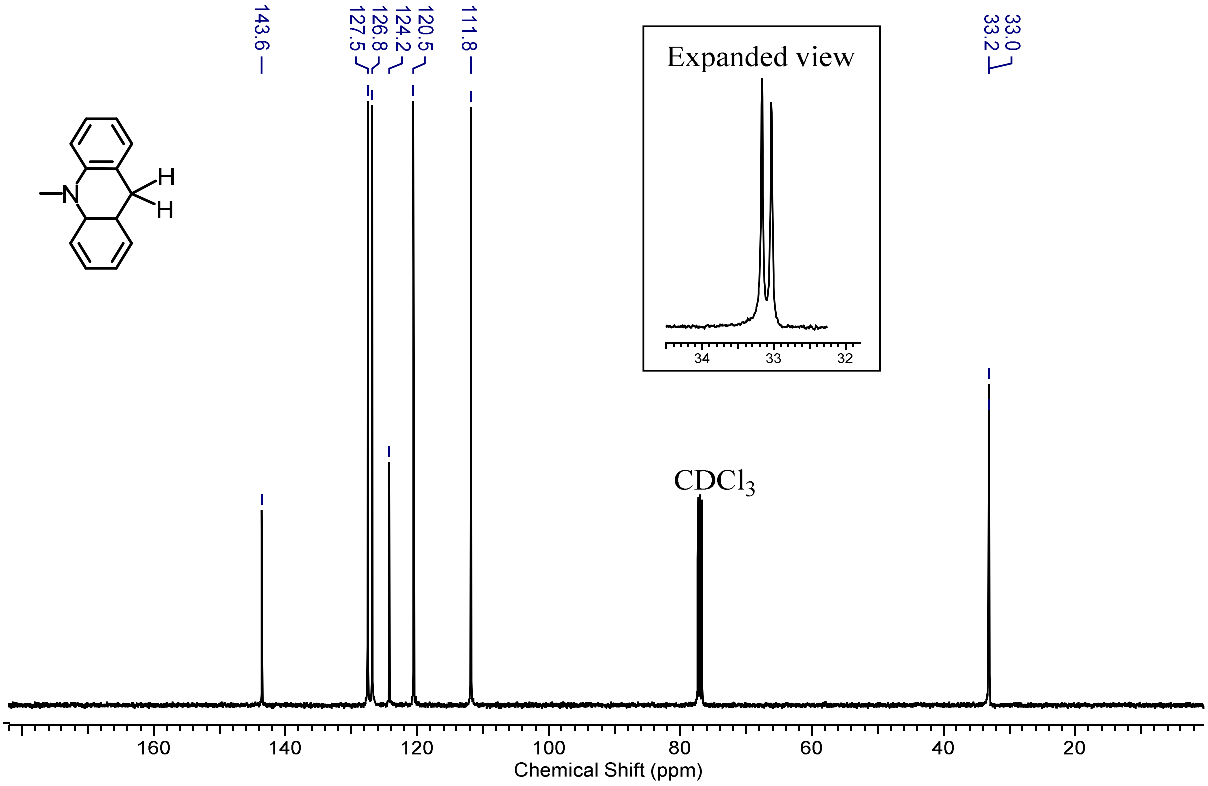

^{1}H-NMR spectrum of 2,4,6-tri-*tert*-butylpyridinium chloride in CH_2Cl_2, with a d_6-DMSO capillary inserted

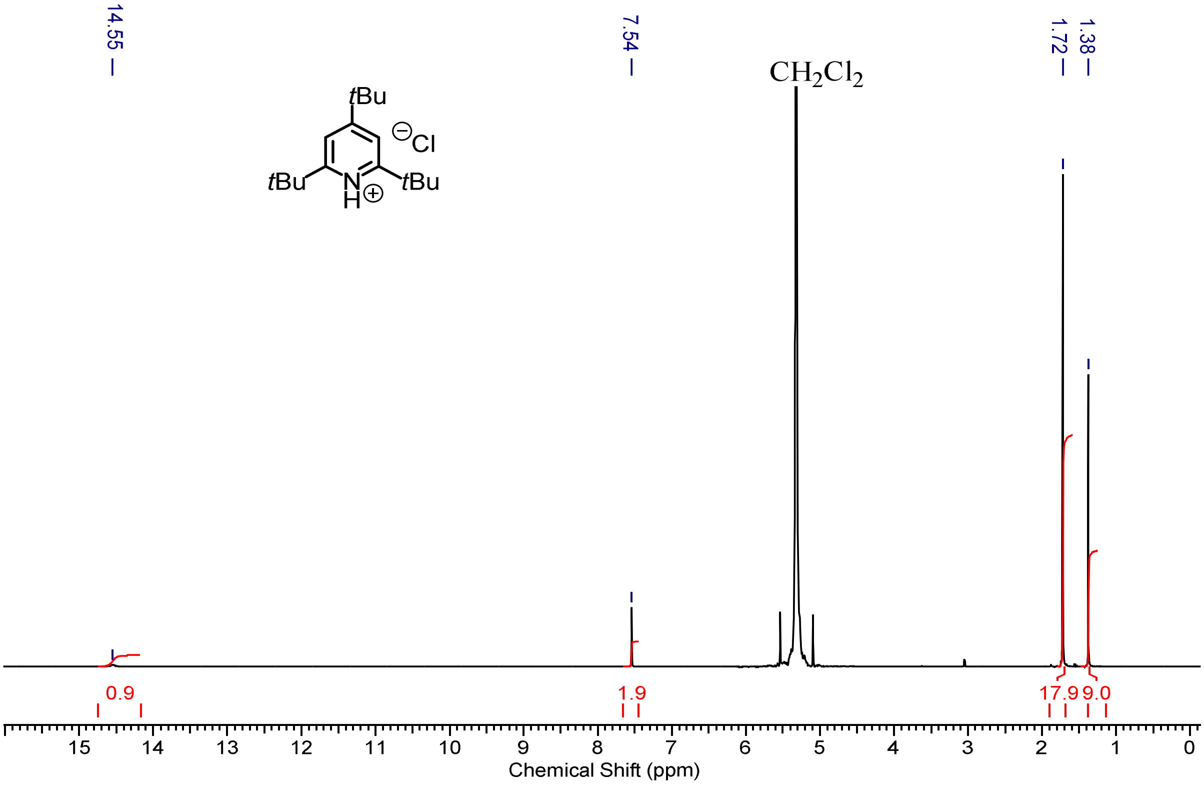

^{1}H and ^{11}B-NMR spectrum of 2,4,6-tri-*tert*-butylpyridinium tetra(3,5-dichlorophenyl)borate in $CDCl_3$

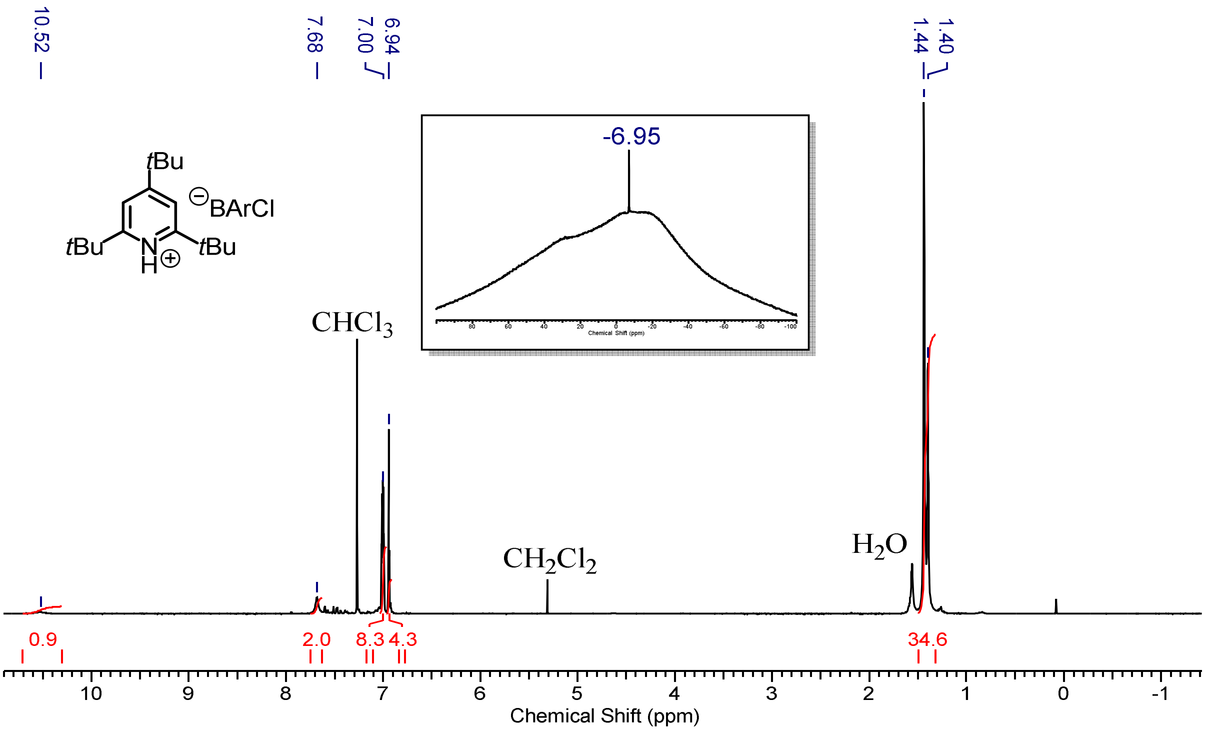

^{1}H and ^{27}Al-NMR spectrum of 2,4,6-tri-*tert*-butylpyridinium tetrachloroaluminate CH_2Cl_2, with a d_6-DMSO capillary inserted

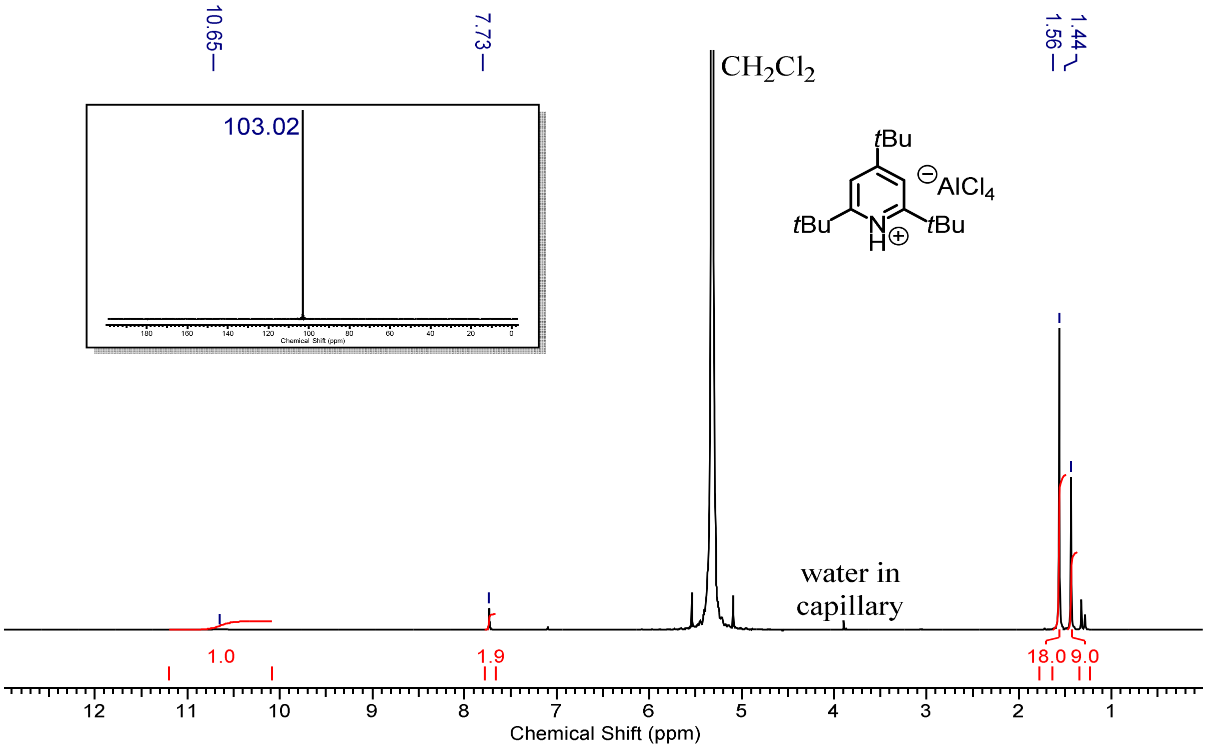

^{1}H-NMR spectrum of 2,6-lutidinium chloride in CH_2Cl_2, with a d_6-DMSO capillary inserted

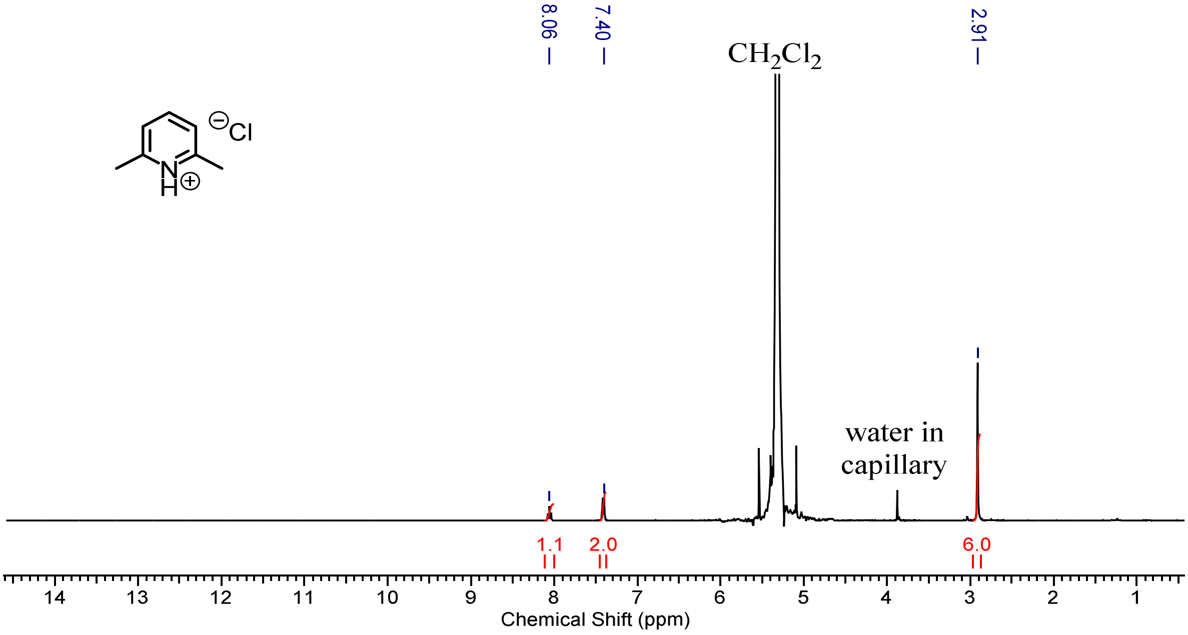

^{1}H and ^{27}Al-NMR spectrum of 2,6-lutidinium tetrachloroaluminate in CH_2Cl_2, with a d_6-DMSO capillary inserted

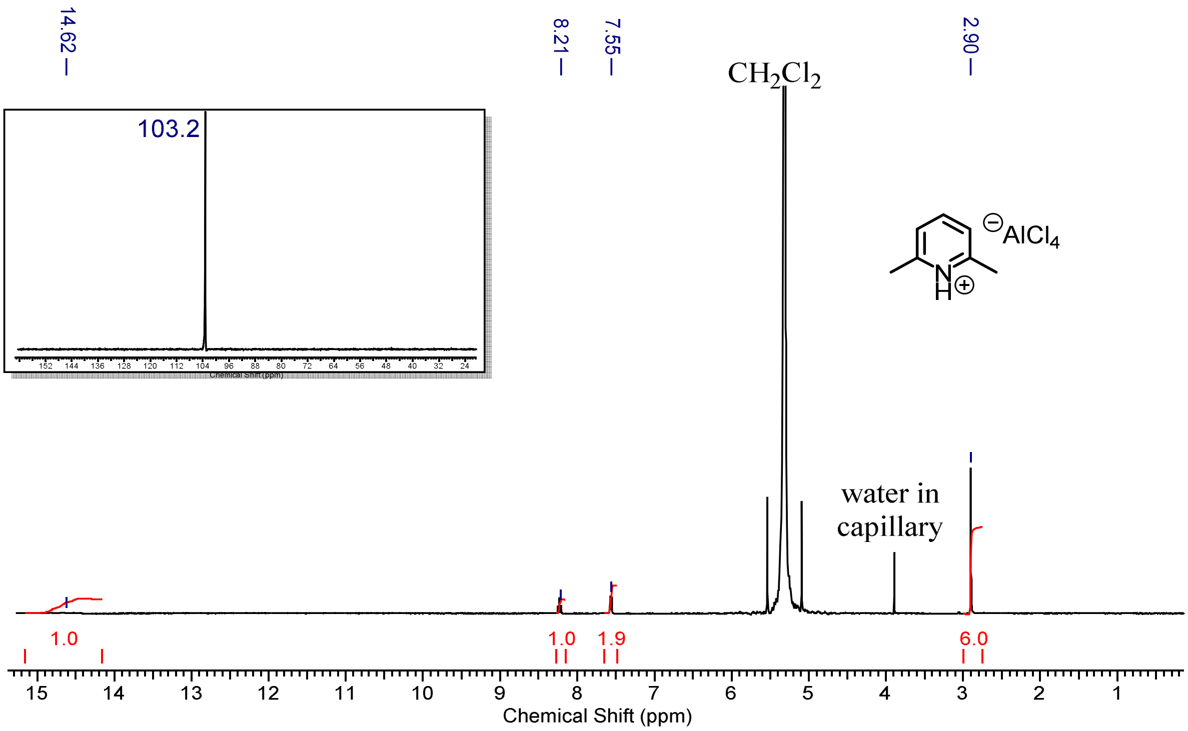

14.62
8.21
7.55
2.90
CH2Cl2
103.2
water in capillary
AlCl4
N
H
1.0
1.0
1.9
6.0
15
14
13
12
11
10
9
8
7
6
5
4
3
2
1
Chemical Shift (ppm)
